# Natural and Synthetic Lactones Possessing Antitumor Activities

**DOI:** 10.3390/ijms22031052

**Published:** 2021-01-21

**Authors:** Younghoon Kim, Sandip Sengupta, Taebo Sim

**Affiliations:** 1 KU-KIST Graduate School of Converging Science and Technology, Korea University, 145 Anam-ro, Seongbuk-gu, Seoul 02841, Korea; YHOON9408@yuhs.ac; 2 Severance Biomedical Science Institute, Graduate School of Medical Science (Brain Korea 21 Project), College of Medicine, Yonsei University, 50 Yonsei-ro, Seodaemun-gu, Seoul 03722, Korea; SENGSANDIP@yuhs.ac

**Keywords:** natural lactones, synthetic lactones, anticancer activities, natural product synthesis, drug discovery

## Abstract

Cancer is one of the leading causes of death globally, accounting for an estimated 8 million deaths each year. As a result, there have been urgent unmet medical needs to discover novel oncology drugs. Natural and synthetic lactones have a broad spectrum of biological uses including anti-tumor, anti-helminthic, anti-microbial, and anti-inflammatory activities. Particularly, several natural and synthetic lactones have emerged as anti-cancer agents over the past decades. In this review, we address natural and synthetic lactones focusing on their anti-tumor activities and synthetic routes. Moreover, we aim to highlight our journey towards chemical modification and biological evaluation of a resorcylic acid lactone, L-783277 (**4**). We anticipate that utilization of the natural and synthetic lactones as novel scaffolds would benefit the process of oncology drug discovery campaigns based on natural products.

## 1. Introduction

Cancer, the second leading cause of death worldwide, has become one of the greatest challenges to global health. The mortality rate that results from cancer is still unacceptably high over the last two decades, responsible for about 8 million deaths per year, and it is predicted to increase to 13 million by 2030 [1,2]. Hence, there is a consistent and urgent need for the discovery of novel and potential chemotherapeutic agents to combat cancer.

Cancer is frequently correlated with disruption in key signaling pathways, which involve extracellular ligands, transmembrane receptors, intracellular signaling protein kinases, and transcription factors, causing aberrant cell proliferation and defective apoptosis induction [3,4]. Recently, the targets and molecular mechanisms of natural product-derived anticancer agents have been extensively investigated [5,6]. Furthermore, the effort has eventually led to the emergence of various natural lactones and their derivatives as potential lead compounds for anticancer therapeutics on account of their potent bioactivities, including cytotoxicity against cancer cells and anti-neoplastic efficacy in in vivo studies [7,8,9].

Lactones are basically chemical entities constituted of cyclic carboxylic esters. Natural and synthetic lactones exhibit a broad spectrum of biological activities such as anti-helminthic, anti-microbial, anti-inflammatory, and anti-tumor activities. Radicicol (**1**), for instance, is a macrocyclic resorcylic acid lactone and its first isolation dates back to 1953 [10]. Initial biological activity of resorcylic acid lactones, however, did not draw much attention from the researchers until kinase inhibition by *cis*-enone-containing resorcylic acid lactones is reported in the late 1990s [11]. 

Starting from a series of resorcylic acid lactones (RALs), several classes of natural lactones including sesquiterpene lactones (SLs), diacylglycerol lactones (DAGLs), and diterpene lactones (DLs) received much attention as potential anticancer agents over the past few decades [12,13,14,15,16]. Nevertheless, the development of those natural products into drug discovery programs is still somewhat limited due to complexity in structures and low pharmacokinetic profile resulted from relatively high lipophilic properties. For this reason, great efforts were made to complete total syntheses of biologically important natural lactones and to establish synthetic approaches to modify complex natural lactone structures [17,18,19]. In the expectation that natural products-based structures would be amply exploited as powerful tools in designing strategies of anticancer drug discovery programs, we herein provide a comprehensive review on the biological activities and chemistry of representative natural and synthetic lactones that are known to exhibit anticancer activities. 

## 2. Biological Activities and Chemistry of Natural and Synthetic Lactones

### 2.1. Reosorcylic Acid Lactones

The resorcylic acid lactones (RALs) are mucotoxins belonging to a family of benzannulated macrolides, which are isolated from various types of fungi. A structural feature of RALs is characterized by a *β*-resorcylic acid scaffold annulated to a 12–14 membered macrolactone [20]. RALs had not been recognized as a significant class of compounds until the late 1990s that molecular targets of a few RALs were revealed. Research on RALs was delighted with the discovery of radicicol (**1**) as an Hsp90 inhibitor in conjunction with the identification of hypothemycin (**2**) and L783277 (**3**) as kinase inhibitors [11,21,22]. Especially, *cis*-enone moiety containing RALs are prone to undergo 1,4-Michael addition with a conserved cysteine residue in the ATP-binding site of the kinases leading to covalent inhibition of the target kinase. The most recognized compounds of this type are hypothemycin (**2**), LL-Z1604-2 (**3**), and L783277 (**4**).

#### 2.1.1. Biological Activities of Radicicol (**1**)

**Figure 1:**
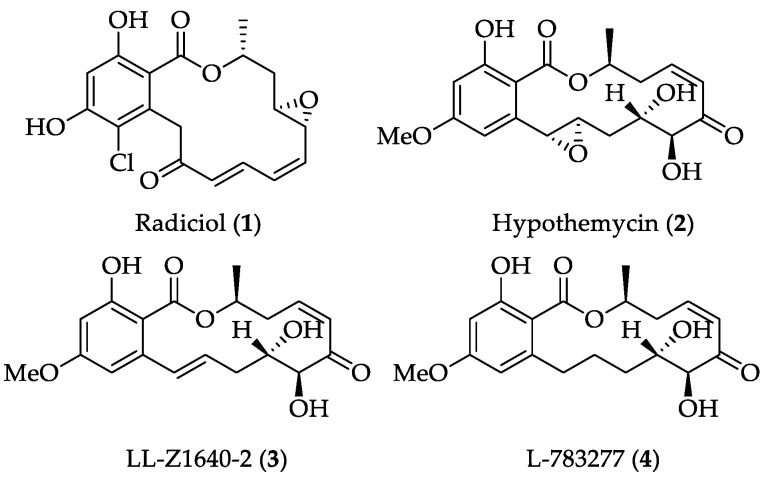
Structures of representative resorcylic acid lactones.

Radicicol (**1**) provided the foundation for research on macrocylic lactones as anticancer agents. In 1953, Radicicol (**1**) was isolated from the fungus *Monosporium bonorden* as the first example of RALs [10]. In 1992, radicicol (**1**) was initially reported to act as an src kinase inhibitor, which triggered research interest in RALs and other classes of macrocyclic lactones [23]. However, radicicol (**1**) was later revealed to selectively inhibit HSP90 function by binding to its N-terminal ATP pocket and the effects of radicicol (**1**) on modulating protein kinase activity turned out to be indirect as many protein kinases require HSP90 for proper folding [24]. Although radicicol (**1**) does not possess direct kinase inhibitory activity, it provided the foundation for research on macrocylic lactones as anticancer agents. Owing to radicicol (**1**), there are currently several examples of well-studied RALs, namely: hypothemycin (**2**), LL-Z1640-2 (**3**), and L-783277 (**4**) that are known to inhibit mitogen-activated protein kinases by the use of a *cis*-enone moiety in an ATP-competitive manner (Figure 1).

#### 2.1.2. Chemistry of Radicicol (**1**)

**Scheme 1:**
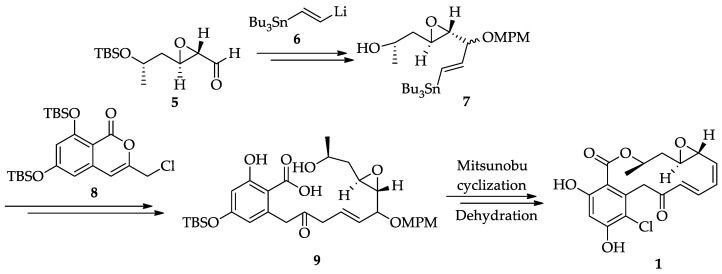
The total synthesis of radicicol (**1**) according to Lett and Lampilas [25].

Affirmation of the structure was achieved by the total synthesis accomplished by Lett and Lampilas in early 1992 and followed by some improvement and modification. Lett’s synthetic protocol utilized a key intramolecular Mitsunobu reaction to construct the 14-member macrocycle (Scheme 1). Cyclization precursor **9** would be synthesized through a palladium-catalyzed coupling reaction of chlorinated isocoumarin **8** and vinyltin substrate **7**. Compound **7** was obtained through the addition of lithium vinyltin **6** to aldehyde **5** already carrying the epoxide with the desired stereochemistry in place. involved 18 linear steps resulting in a 4.7% overall yield [25].

**Figure 2:**
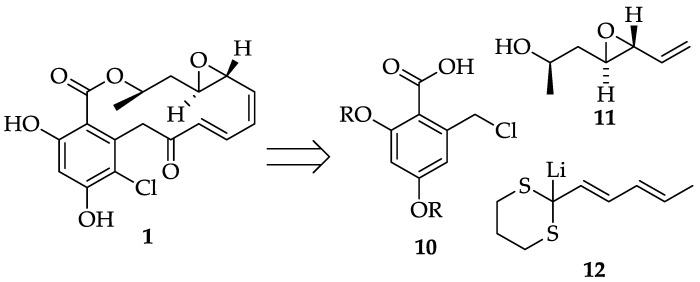
Retrosynthetic analysis according to Danishefsky et al. [26].

In 2000, the Danishefsky group did concise asymmetric syntheses of radicicol (**1**) and monocillin I [26]. Their synthetic strategy relies on a convergent three-stage assembly of the 14-membered lactone which has, as a key transformation, a novel ring-forming metathesis reaction utilizing a vinyl epoxide (Figure 2).

**Scheme 2:**
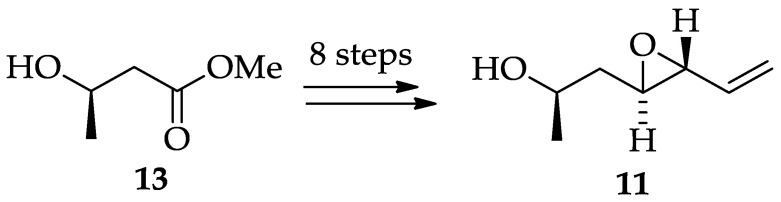
Synthesis of intermediate **11** [26].

The synthesis commenced with construction of the chiral homochiral allylic alcohol **11** from methyl (*R*)-3-hydroxybutyric acid (**13**) in 8 steps in 49% yield (Scheme 2). 

**Scheme 3:**
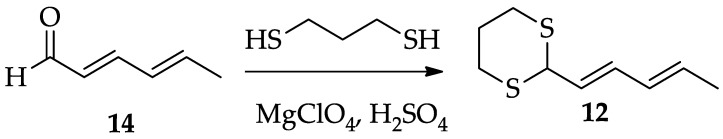
Synthesis of intermediate **12** [26].

The allylic dithiane **12** was prepared in one step from commercially available 2,4-hexadienal **14** with 64% yield (Scheme 3). 

**Scheme 4:**
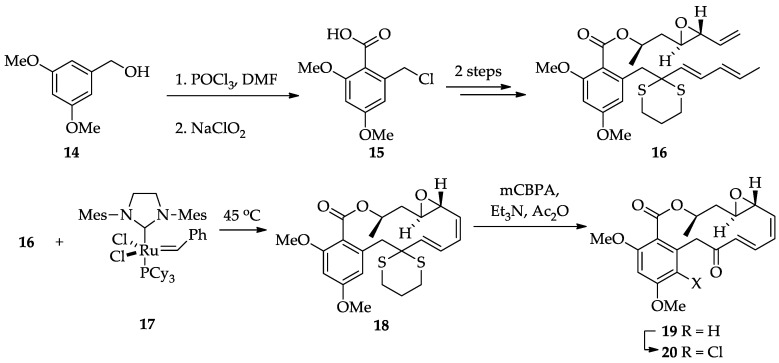
Synthesis of radicicol dimethyl ether (**20**) [26].

Formylation followed by oxidation and conversion of the alcohol to chloride was effected with 3,5-dimethoxybenzyl alcohol **14** to give the desired acid **15** (Scheme 4). Intermediate **16** was synthesized in 2 steps from **15** with 48% yield. Ruthenium-based olefin metathesis catalyst was used to get the desired 14-membered lactone **18** stereospecifically in 55% yield. Oxidation and crude monosulfoxide was exposed to the action of Ac_2_O, Et_3_N, and H_2_O to give the desired ketone dimethyl monocillin I (**19**, 70%). Regiospecific chlorination of the aromatic ring then produced radicicol dimethyl ether (**20**).

**Scheme 5:**
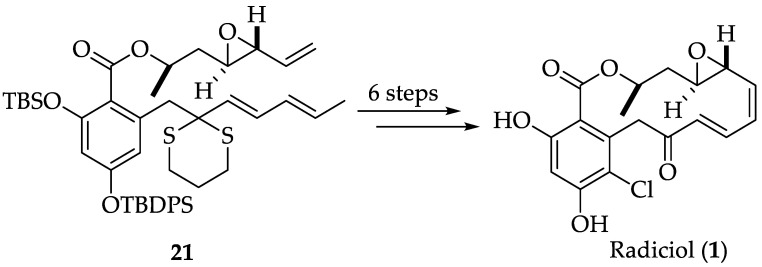
Synthesis of radicicol (**1**) according to Danishefsky et al. [27].

However, they were able to reform the natural products via the similar strategy used before except the modification of protection groups in the aromatic ring **21** (Scheme 5). They obtained the final radicicol (**1**) by treatment with base, in 7.5% yield over 14 steps (longest linear sequence) [27]. 

**Scheme 6:**
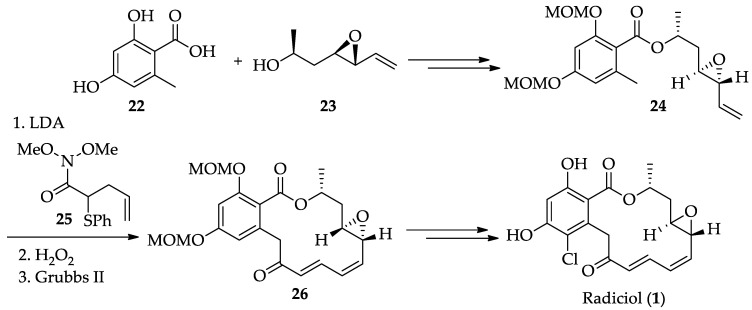
Synthesis of radicicol (**1**) according to Winssinger et al. [28].

Winssinger’s total synthesis of radicicol (**1**) was reported in 2005 [28]. 2-hydroxy toluic acid **22** under Mitsunobu condition with **23** and protection gave **24** (Scheme 6). Reaction of toluate **24** using two equivalents of LDA followed by addition of Weinreb amide **25** afforded cyclization precursor, which after elimination and Grubbs reaction produced **26** with 64% yield in 3 steps. Radicicol (**1**) was obtained from intermediate **26** in 3 steps with 43% yield.

**Scheme 7:**
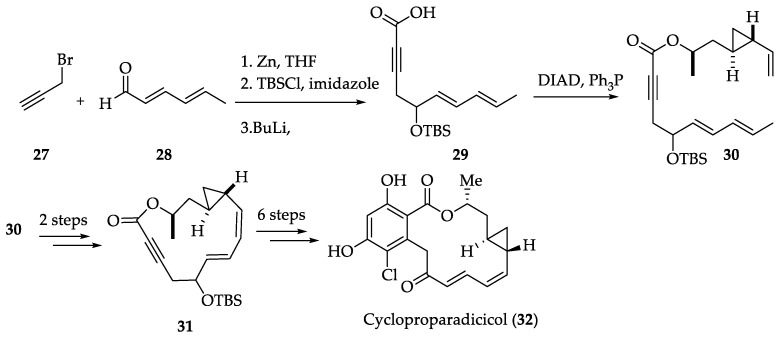
Synthesis of cycloproparadicicol (**32**) [29].

Later the Danishefsky group decided to “edit” the epoxide and replace it with a cyclopropyl group in a highly convergent and efficient three-stage protocol for the syntheses of radicicol (**1**) and cycloproparadicicol (**32**) [29]. The Hsp90 inhibition activity dropped somewhat from the natural product (IC_50_ = 20–23 nM for radicicol (**1**) vs. IC_50_ = 160 nM for cycloproparadicicol (**32**)), yet cycloproparadicicol (**32**) was still very potent. As with the epoxide, inversion of the cyclopropane stereochemistry diminishes the activity (Scheme 7). 

The central element of their plan was the building of an “ynolide” intermediate and its advancement to the benzomacrolide by a Diels–Alder cycloaddition. Reformatsky-like condensation of propargyl bromide **27** with **28**, followed by protection and subsequent reaction of the lithium alkynide ion, gave them the acid intermediate **29**, which under Mitsonobu condition, furnished ester **30**. Cyclopropane-containing cobalt complex of **30** cyclized to give **31** in 39% yield, as a 2:1 mixture of two diastereomers and finally after 6 steps provided them cycloproparadicicol (**32**).

**Figure 3:**
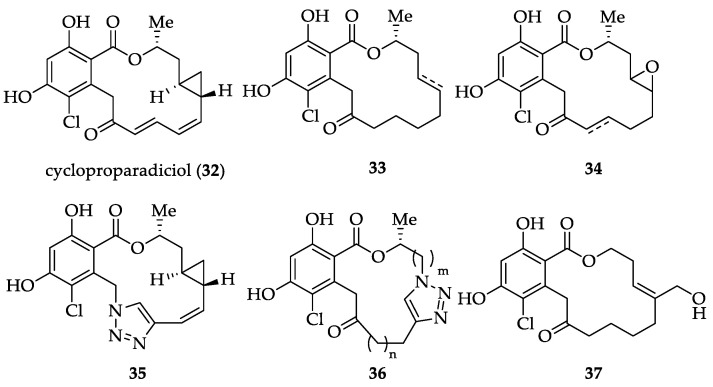
Radicicol (**1**) analogs as Hsp90 inhibitors [30].

Unfortunately, radicicol (**1**) exhibited no in vivo activity, which might be due to the highly sensitive functionality present in the molecule, including an epoxide and a conjugated dienone, both of which are readily metabolized. As a result, significant efforts have focused on synthesizing radicicol (**1**) analogs, which retain the potency, but with improved metabolic stability (Figure 3).

**Figure 4:**
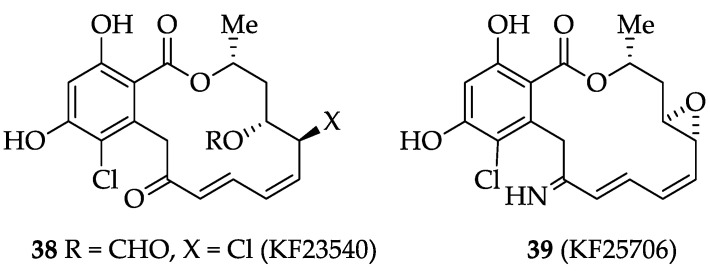
Halohydrin and oxime derivatives of radicicol (**1**) [31].

A series of halohydrin and oxime derivatives of radicicol (**1**) were prepared and evaluated for their antitumor activities in vitro and in vivo (Figure 3) [31]. Although halohydrin derivative **38** was inactive, oxime derivatives showed in vivo antitumor activities, with hydroxime **39** being the most active. KF25706 (**39**) was further evaluated for its antitumor activity against various tumor models by iv (intravenous) injections (Figure 4).

**Scheme 8:**
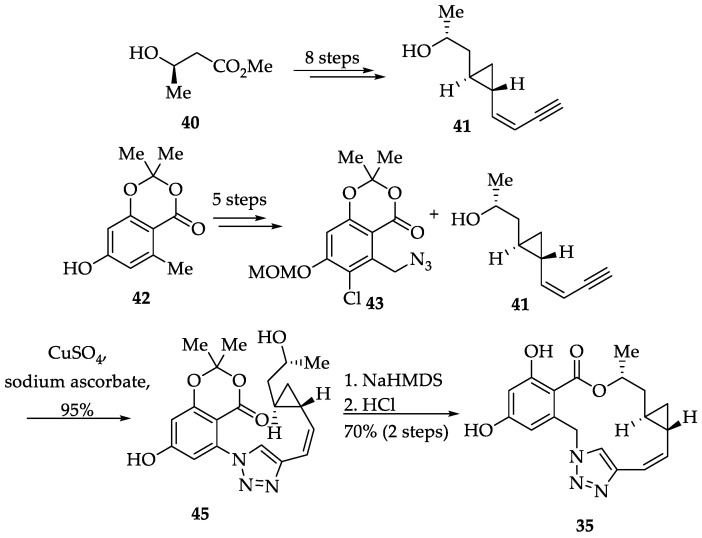
Synthesis of triazole derivative (**35**) of radicicol (**1**) [32].

Triazole derivative (**35**) was synthesized by a copper-mediated cycloaddition between benzyl azide **43** and alkyne **41**, prepared from **40** in 8 steps, gave triazole **45** in near quantitative yield. Base-promoted formation of the macrocycle and subsequent deprotection afforded the RAL triazole **35** in an efficient and highly convergent synthetic route (Scheme 8). Despite the additional H-bonding potential, a decrease in Hsp90 inhibition activity was observed (IC_50_ = 400 nM for **35** vs. 20–23 nM for radicicol (**1**)), although the compound displayed significant in vivo activity. In a similar manner, the Danishefsky group incorporated a triazole ring into RAL analogs, whilst retaining the carbonyl at the 9-position. None of the triazole-containing analogs displayed significant activity [32].

A synthesis of resorcylic acid macrolactam analogs of the natural product radicicol (**1**) is described in which the key steps are the acylation and ring opening of a homophthalic anhydride to give an isocoumarin, followed by a ring-closing metathesis to form the macrocycle. Synthesis of macrolactam analogs of radicicol reported by Moody group in 2014 [33]. 

**Scheme 9:**
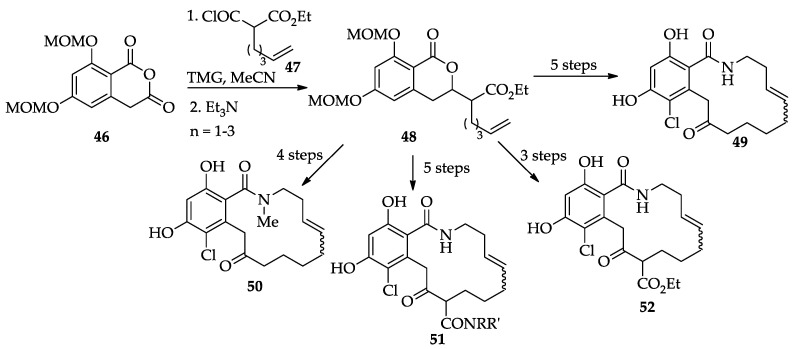
Synthesis of macrolactam analog (**49**–**52**) [33].

Addition of the malonate-derived acid chloride **47** to anhydride **46** gave the acylated intermediate, which spontaneously underwent a cyclization/retrocyclization, with the loss of CO_2_, giving the isocoumarin **48** in 75% yield (Scheme 9). This was readily converted into both the NH and NMe macrolactams **49** and **50**, respectively, and allowed for the incorporation of amide and ester functional groups at C10 (**51** and **52**, respectively), giving additional H-bonding potential.

The macrolactams were indeed more metabolically stable (43% metabolism for macrolactam **50** vs. 84% for radicicol (**1**) after 15 min with human liver microsomes and NADH/NADPH) and, significantly, were also often superior to the corresponding other natural macrolactones in terms of activity [34].

#### 2.1.3. Biological Activities of Hypothemycin (**2**) and LL-Z1640-2 (**3**)

Hypothemycin (**2**) is a *cis*-enone containing natural macrocylic resorcylic acid lactone, which is an antifungal metabolite isolated from *Hypomyces trichothecoides*. A comprehensive study conducted by groups of Tanaka and Sonoda reported that treatment of hypothemycin (**2**) on v-KRAS-transformed NIH3T3 (DT cells) dramatically decreased the amount of cyclin D1 protein [35,36]. The groups also suggested cyclin D1 as a key molecule involved in RAS downstream signaling and hypothemycin (**2**) might act as a RAS-signaling pathway inhibitor by facilitating ubiquitinating process of cyclin D1 in DT cells. Another study of hypothemycin (**2**) on its kinase specificity revealed that most kinases inhibited by hypothemycin (**2**) contained a conserved cysteine residue at kinase active site, which is corresponding to the cysteine 166 of ERK1/2 and they were inhibited in a time-dependent manner suggesting covalent inhibition of the kinases through the formation of a Michael adduct [37]. Particularly, hypothemycin (**2**) displayed low nanomolar K_i_ values against five kinases (MEK1, MEK2, FLT1, and KDR) and high nanomolar K_i_ values on TRKB. These findings furnished explanations of the behavior of hypothemycin (**2**) as a RAS-signaling pathway inhibitor [36]. Moreover, it is also of significance that hypothemycin (**2**) could not inhibit GSK3*β*, which possesses a conserved cysteine [37].

LL-Z1640-2 (**3**), also known as C292 and 5-(*Z*)-7-oxozeaenol), is a radicicol-related anti-protozoan isolated from an unidentified fungi source. LL-Z1640-2 (**3**) can selectively inhibit transforming growth factor (TGF)-*β*-activated kinase 1 (TAK1) with high potency (TAK1, IC_50_ = 8.1 nM) [38]. TAK1 belongs to the upstream of mitogen-activated protein kinases (MAPK) called MAPKK-K family. TAK1 is mainly involved in the activation of JNK and P38 MAPK by activating the following MKKKs: MKK3, MKK4, MKK6, and MKK7. LL-Z1640-2 (**3**) strongly inhibited JNK/p38 pathway, however, it did not display any effect on ERK kinase activation induced by epidermal growth factor (EGF) in Hela cells. This provided evidence that LL-Z1640-2 (**3**) does not directly block JNK/p38 pathway, but rather acts at the upstream of the MAPKK level. The JNK/P38 pathways are important in biological responses such as inflammation, apoptosis, and cell differentiation and their aberration might bring about diseases related to inflammatory disease, neurodegenerative disorder, and cancer progression [39]. Therefore, LL-Z1640-2 (**3**) is regarded as a potential therapeutic agent to treat such diseases and can also be used as a great toolbox for signal transduction research [40].

#### 2.1.4. Chemistry of Hypothemycin (**2**) and LL-Z1640-2 (**3**)

**Scheme 10:**
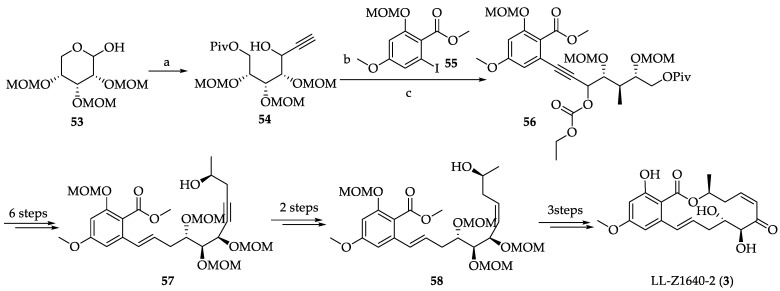
Synthesis of LL-Z1640-2 by Tatsuta et al. [41]. Reagents and conditions: (a) TMS-acetylene, *n*-BuLi, BF_3_·Et_2_O, THF, −78 °C, rt; (b) Pd(OAc)_2_, CuI, Ph_3_P, Et_3_N, 2 h; (c) ClC(O)OEt, pyridine, 0 °C, 1 h.

The first total synthesis of LL-Z1640-2 (**3**) was reported by Tatsuta and co-workers in 2001 [41]. Their synthesis commencing from the addition of *tris*-MOM-protected D-ribose **53** and lithiated TMS-acetylene to propargylic alcohol **54** followed by protection, which was then undergone a Sonagashira reaction to aromatic iodo **55** to get **56** and after 6 steps generate propargylic alcohol **57** (Scheme 10). Target structure produced by a Mukaiyama macrolactonization reaction of a protected seco acid **58** in 3% overall yield for the longest linear sequence of 19 steps (from D-ribose).

**Figure 5:**
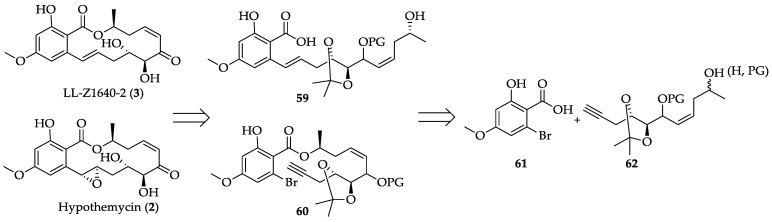
Retrosynthesis of hypothemycin (**2**) and LL-Z1640-2 (**3**) according to Selles and Lett [42].

An alternative approach to the synthesis of LL-Z1640-2 (**3**) and hypothemycin (**2**) has been reported by Selles and Lett [42]. Here, the macrocyclic framework of hypothemycin (**2**) and LL-Z1640-2 (**3**) would be constructed either through Mitsunobu-based macrolactonization from seco acid **59** or, alternatively, through a Suzuki-type macrocyclization of ester **60**. Both **59** and **60** would be derived from o-bromo benzoic acid **61** and intermediate **62** (Figure 5). The synthesis developed by Selles and Lett provided LL-Z1640-2 (**3**) and hypothemycin (**2**) in overall yields of 1% and 0.17%, respectively, in 26 steps for the longest linear sequence. 

**Scheme 11:**
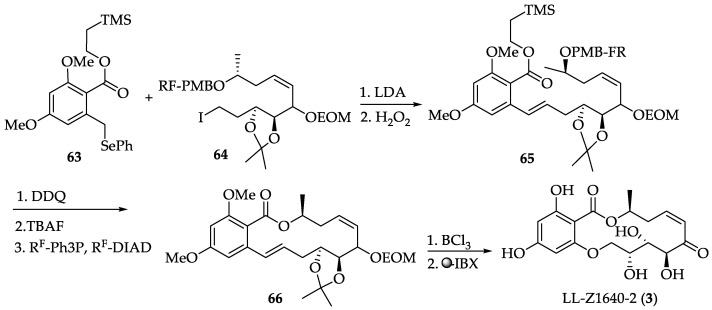
Concise synthesis of LL-Z1640-2 (**3**) by Wissinger et al. [43].

In 2007, the Winssinger group developed a concise synthesis of LL-Z1640-2 (**3**) and related resorcylic acid lactones using a combination of fluorous tag-isolation and polymer-bound reagents, thus making the chemistry amenable to high throughput synthesis [43]. In the synthetic scheme their advanced intermediate **64**, was alkylated in excellent yield with aromatic fragments **63** previously deprotonated by LDA (Scheme 11). The selenide was then oxidized and eliminated to afford compounds **65** and purification done by using simply loaded on fluorous-silica columns. The PMB group was removed by DDQ, while the TMSE ester was removed with TBAF. The hydroxyacids thus obtained were engaged in Mitsunobu macrocyclisations using fluorous-tagged triphenyl phosphine and diazo dicaboxylate, thus yielding the macrocycles **66** after a fluorous solid-phase extraction. Finally, deprotection and oxidation yielded a natural product, LL-Z1640-2 (**3**) in good yield [43].

**Scheme 12:**
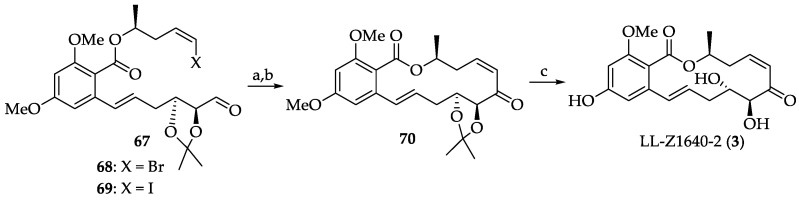
Total Synthesis of LL-Z1640-2 (**3**) by Thomas et al. [44]. Reagents and conditions: (a) CrCl_2_, NiCl_2_ (cat.), DMF; (b) Dess–Martin periodinane, CH_2_Cl_2_, rt; (c) BCl_3_, CH_2_Cl_2_.

Total synthesis of LL-Z1640-2 (**3**) utilizing a late-stage intramolecular Nozaki–Hiyama–Kishi reaction was reported by Thomas et al. in 2010 [44]. Their route depended on an aryl selenide nucleophile for a precedented sequence of alkylation, oxidation, and elimination to generate the requisite trans-olefin scaffold (Scheme 12). Subsequent Mitsunobu esterification would provide access to an advanced intermediate **68** and **69** poised for macrocyclization with the intramolecular Nozaki–Hiyama–Kishi (NHK) reaction. Finally, oxidation and deprotection completed natural product LL-Z1640-2 (**3**) synthesis. 

**Scheme 13:**
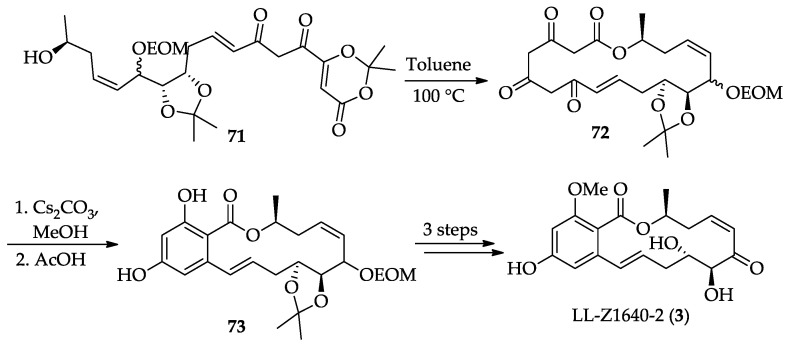
Total synthesis of LL-Z1640-2 (**3**) by Barret et al. [45].

In 2010, Barrett group reported total synthesis of LL-Z1640-2 (**3**) via consecutive macrocyclization and transannular aromatization [45]. According to their synthetic protocol thermolysis of diketo-dioxinone **71** resulted in generation of ketene intermediate, which was efficiently trapped intramolecularly by the alcohol to provide the 18-membered macrocyclic lactone **72** (Scheme 13). Regioselective methylation, followed by deprotection and oxidation gave LLZ1640-2 (**3**) in 51% yield over three steps. 15-step biomimetic total synthesis of the TAK-kinase inhibitor LL-Z1640-2 (**3**) from commercially available starting materials was reported.

**Scheme 14:**
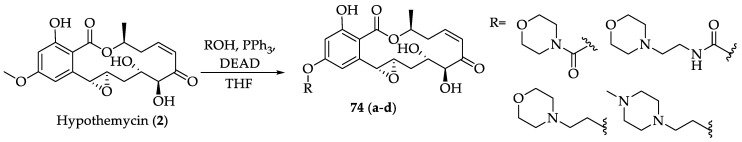
The synthesis of semi-synthetic derivatives of hypothemycin (**2**) [46].

In 2007, a number of semi-synthetic derivatives of this natural product hypothemycin (**2**) and their biological assessment has been reported [46]. Interestingly, derivatives **74a**–**d** showed similar antiproliferative activity as hypothemycin (**2**) in most cases investigated, which indicates that substituents on C4-O do not interfere with target protein binding and may thus be used to modulate the physico-chemical properties of hypothemycin (**2**) without impairing the desired biological activity (Scheme 14). 

**Scheme 15:**
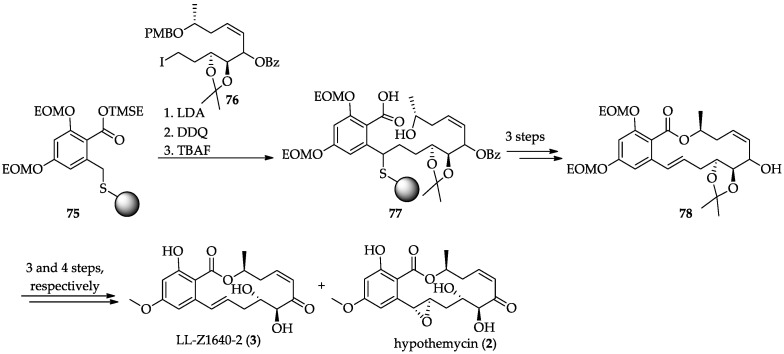
Divergent synthesis of hypothemycin (**2**) and LL-Z1640-2 (**3**) [47].

Herein, divergent syntheses of resorcylic acid lactones were reported, presenting an effort on accessing macrocycles bearing an alkene, or epoxide at the benzylic position from a common benzylic sulfide intermediate to access both LL-Z1640-2 (**3**) and hypothemycin (**2**) [47]. Starting from resin-bound intermediate **75**, the toluate position was alkylated with iodide **76** to obtain polymer-bound intermediate **77** (Scheme 15). Mitsunobu cyclisation followed by the release of sulfur resin under oxidative condition gave **78** which was oxidized to the desired *cis*-enone under slightly more forceful conditions by using DMP in CH_2_Cl_2_ under reflux and led to final compounds both natural products LL-Z1640-2 (**3**) and hypothemycin (**2**).

**Scheme 16:**
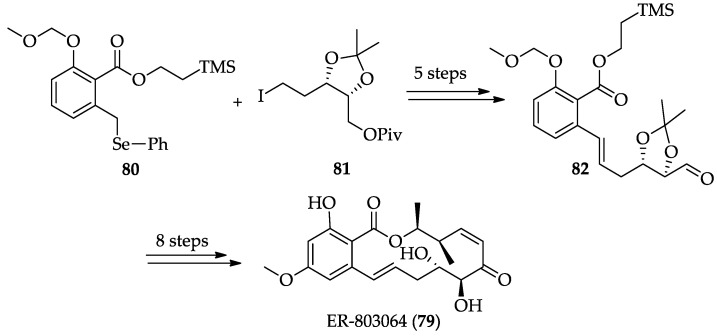
Synthesis of ER-803064 (**79**) [48].

In 2009, a serum and plasma-stable resorcylic acid analog of ER-803064 (**79**) was discovered [48]. The addition of the (*S*)-Me group at C4 has resulted in a dramatic improvement in metabolic stability with retention of bioactivity (Scheme 16). Aromatic ester **80** and aliphatic iodo **81** were coupled and further steps were employed to get aldehyde **82** in 5 steps which after 8 synthetic steps gave final ER-803064 (**79**).

**Scheme 17:**
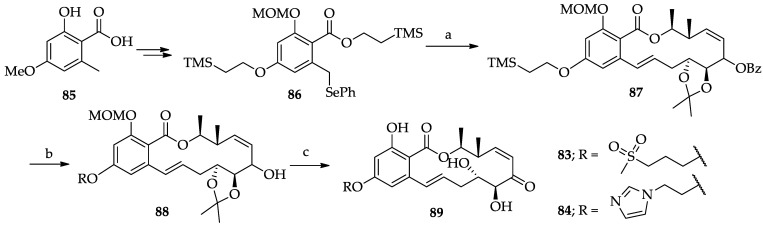
Synthesis of LL-Z1650-2 (**3**) analogs (**83** and **84**) [49]. Reagents and conditions: (a). (i). LiHMDS, acyclic iodide, (ii). *m*CPBA, Et_3_N, (iii). TBAF, (iv). 2-Cl-1-Me pyridinium iodide; (b). (i). TBAF, (ii). Mitsunobu reaction, (iii). NaOH; (c). (i). PCC/Swern reaction, (ii). HCl.

In 2010, the Wang group reported a potent in vitro lead compound, **83** and **84**, fully synthetic analogs of natural product LL-Z1640-2 (**3**). The analog **84** maintained the metabolic stability, regained full in vitro potency similar to natural product, and had significant improvement in in vivo potency (Scheme 17). Their synthesis started from commercially available methyl orsellinate (**85**) and after 5 steps afforded aromatic selenide intermediate **86** which was then coupled with acyclic iodide in the presence of LiHMDS followed by *m*CPBA oxidation, TBAF deprotection, and lactone cyclization under the influence of Mukaiyama’s reagent to provide lactone **87**. Deprotection followed by C14 substitutions was done through Mitsunobu reaction and benzoyl group was then deprotected under basic conditions to produce allylic alcohol **11**. Finally, oxidation followed by deprotection led to final analog **83** and **84** [49].

**Scheme 18:**
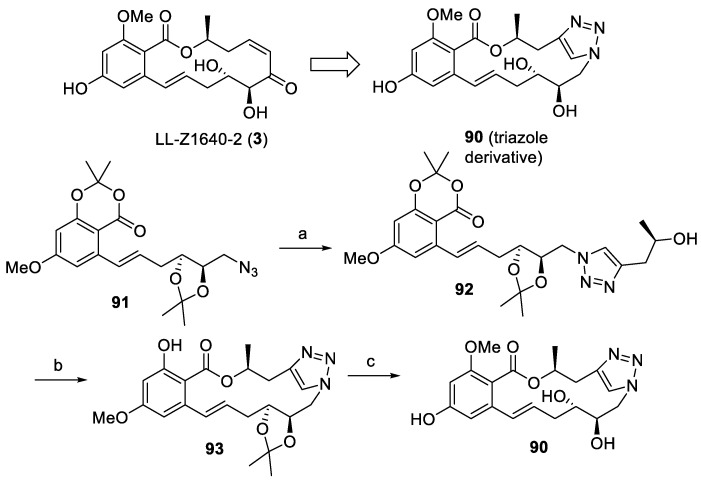
Synthesis of triazole derivative (**90**) of LL-Z1650-2 (**3**) [50]. Reagents and conditions: (a). (i). CuSO_4_·H_2_O, *t*-BuOH/H_2_O, sodium ascorbate; (b). NaH, THF; (c). 1 N HCl, MeOH/THF.

In 2014, synthesis and biological studies of a triazole analogue (**90**) of LL-Z1640-2 (**3**) were reported by the Chen group in 6 linear steps in 18% overall yield [50]. The triazole analog (**90**) showed good activity (IC_50_ = 7.2 μM) against MNK2 kinase, which is an emerging target for cancer chemotherapy (Scheme 18). Azido intermediate **91** which was synthesized from 2-deoxy d-ribose acetonide and phloroglucinol carboxylic acid treated with excess (*S*)-pent-4-yn-ol, under the standard CuSO_4_/sodium ascorbate conditions, led to a smooth click reaction to afford the desired triazole **92** followed by successful macrocyclization and intramolecular transesterification with NaH. Final deprotection of the acetonide **93** concluded a concise synthesis of the triazole analog **90**.

**Scheme 19:**
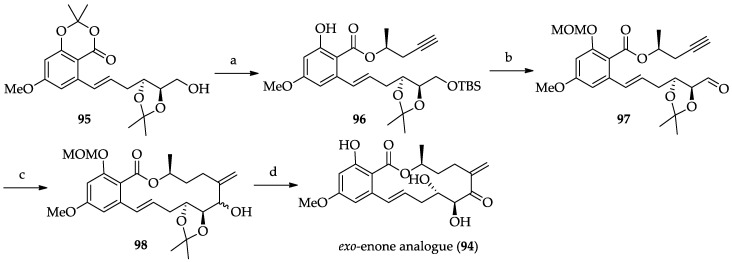
Synthesis of *exo*-enone analog (**94**) of LL-Z1640-2 (**3**) [51]. Reagents and conditions: (a). (i). TBSCl, imidazole, (ii). NaH, THF; (b). (i). MOMCl, NaH, (ii). TBAF, THF, (iii). DMP, DCM; (c). NiCl_2_, CrCl_2_, PPh_3_, H_2_O, DMF; (d). (i). DMP, DCM, (ii). HCl, MeOH.

In 2016, an efficient synthesis of an *exo*-enone analog (**94**) of LL-Z1640-2 and evaluation of its protein kinase inhibitory activities were reported by the Chen Group [51]. The synthesis has been achieved using a Ni-catalyzed regioselective reductive coupling macrocyclization of an alkyne–aldehyde as a key step (Scheme 19). The synthesis of **94** commenced with the reported intermediate alkene **95** which after protection and transesterification in the presence of NaH gave intermediate **96** in nearly quantitative yield. Alkyne–aldehyde **97** was readily synthesized in 3 steps from **96**. As per reported condition **97** under moderate dilution (0.013 M), gratifyingly provided the desired macrocyclization product **98** in 57% isolated yield. This reaction proceeds via an alkenylnickel species formed by a regioselective hydronickelation of the alkyne. Finally, oxidation and deprotection furnished the required *exo*-enone analog **94**.

#### 2.1.5. Biological Activities of L-783277 (**4**)

L-783277 (**4**) is a naturally occurring 14-membered resorcylic acid lactone isolated from *Helvella acetababulum*, a species of fungus in the family Helvellaceae. L-783277 (**4**) is especially known for its highly potent inhibitory activity against MEK (IC_50_ of 4 nM). The unexceptional binding affinity of L783277 (**4**) to kinases is mainly due to the presence of the electrophilic *cis*-enone moiety that forms Michael adducts with conserved cysteine residue residing near the DFG motif of the kinases. As a good comparison, a C7′-C8′ *trans*-enone analog of L-783277 (**4**), exhibited 75-fold decreased activity against MEK (IC_50_ of 300 nM). Moreover, L-783277 (**4**) was reported to possess potent inhibitory activities against several kinases including VEGFR2/3, FLT1/3/4, MEK1/2, KDR, and PDGFRα but with low kinome selectivity [47,52].

We investigated the effects of a structural nature of L-783277 (**4**) on kinase inhibitory activities in three subsequent researches [52,53,54]. In an effort to expand our MEK kinase program and to conduct a detailed SAR study with L-783277 (**4**) derivatives, we developed an efficient synthetic route toward L-783277 (**4**) [54]. With this effective synthetic approach distinctive from those reported in precedent literature at the time, we were able to obtain tens of milligrams of L-783277 (**4**) by simple purification method using flash silica chromatography [47,55]. 

**Figure 6:**
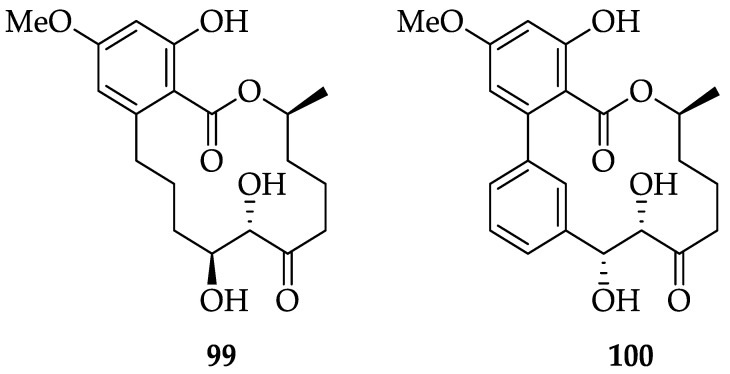
Structures of L-783277 (**4**) derivatives (**99** and **100**).

We noticed an observation that hypothemycin (**2**) could not inhibit GSK3β possessing a conserved cysteine while it could inhibit kinases without conserved cysteines (Src, TrkA, and Trk) [37]. Accordingly, we further investigated the structure of L-783277 (**4**), based on our postulation that the reversible binding of RALs might also play a key role in determining the inhibitory activities and overall selectivity toward kinases. To test the hypothesis, we synthesized a reversible version of L-783277 (**4**), **99**, in which the alkene moiety of the *cis*-enone of L-783277 (**4**) was saturated and a number of analogs derived from **99** (Figure 6). Kinome-wide selectivity profiling and biochemical assays were conducted with **99** and results showed that **99** inhibits activin A-like kinase 1 (ALK1) more potently than its parent compound, L-783277 (**4**), does and it displayed a better kinase selectivity against a panel of 342 kinases. ALK1 is a serine threonine kinase expressed predominantly in endothelial cells and it functions as a receptor for bone morphologic protein 9 (BMP9). Binding of the ligand to ALK1 activates Smad4 by phosphorylating Smad1/5/8, which upregulates transcription of various target genes involved in angiogenesis [56,57]. Due to the significance of ALK1 in regulation of tumor angiogenesis, it has been regarded as a potential therapeutic target for the development of anticancer agents [58]. Although dorsomorphin and LDN193189 showed inhibitory activities against BMP type I receptor kinase and could inhibit BMP-related Smad and non-Smad signaling, inhibitory activities displayed by the two were so broad that it also inhibited other signaling pathways, inducing adverse events brought by off-target effects. In this study, we reported that **99** is a potent and selective ALK1 inhibitor, which acts by selectively blocking BMP9-induced ALK1 signaling in C2C12 cells [52]. It is also highly noteworthy that ALK1 is one of the kinases that has no conserved cysteine residue equivalent to Cys168 of ERK2. Therefore, the selectivity of **99** is achieved by its inability to form a Michael adduct with the most kinases. Moreover, greater conformational flexibility of **99** enables it to adopt superior binding mode, which is the reason that the **99** shows higher activity relative to L-783277 (**4**) against ALK1 (IC_50_ ALK1 = 62 nM and 126 nM for **99** and L-783277 (**4**), respectively). 

Our experience in discovering the first reversible version of L-783277 (**4**) as a potent and selective ALK1 inhibitor encouraged us to focus on further development of L-783277 (**4**) analogs with increased kinome-wide selectivity. We previously observed that L-783277 (**4**) strongly inhibited VEGFR3/2 and FLT3 but it displayed poor kinome-wide selectivity. The tyrosine kinase vascular endothelial growth factor receptor3 (VEGFR3) and its homolog, VEGFR2, take part in tumor lymphangiogenesis [59] and angiogenesis [60], respectively. Because inhibiting both angiogenesis and lymphangiogenesis is claimed to be a reasonable strategy to treat cancer [61,62], we aimed to discover novel L-783277 (**4**) derivatives that selectively inhibit VEGFR2/3 and FLT3 with high potency. Our strategy implemented in order to achieve this goal was to rigidify the structure of 14-membered lactone scaffold of L-783277 (**4**), anticipating that structural rigidity could potentially enhance kinome-wide selectivity of L-783277 (**4**). We synthesized 11 novel L-783277 (**4**) derivatives containing a phenyl ring incorporated in the 14-membered chiral resorcylic acid lactone ring system to assess this hypothesis. Among these derivatives, **100** exhibited the highest potencies against VEGFR3, VEGFR2, and FLT3 and excellent kinome-wide selectivity [54]. Moreover, results of corneal assay conducted with **100** demonstrated effective suppression of both lymphangiogenesis and angiogenesis, indicating that **100** is a potential and unique template to be used in the development of therapeutically active and selective compound, targeting both lymphangiogenesis and angiogenesis [54].

Most natural macrocyclic lactones exert physiological effects by covalently binding to various biological targets. This, however, is often regarded as a major reason behind poor selectivity among substrates and undesired dose-dependent toxicities. Our findings obtained during the course of structural modification and biological evaluation of L-783277 (**4**) indicate that simple transformation made on a macrocyclic lactone can dramatically increase substrate specificity and potency against a certain protein of interest. It is also of significance that exploration of such an effort was firstly demonstrated by our group [52,54]. Furthermore, we anticipate that our works on L-783277 (**4**) and its derivatives (**99** and **100**) provide a promising basis for further developments of natural and synthetic lactones, along with other natural products, as potential anticancer agents.

#### 2.1.6. Chemistry of L-783277 (**4**)

**Scheme 20:**
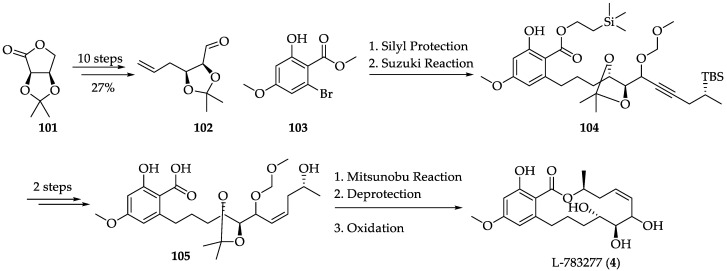
First total synthesis of L-783277 (**4**) by Altmann et al. [55].

The first synthesis of L-783277 (**4**) was achieved by Altmann et al. in 2008, through the successful exploration of Suzuki coupling and a late-stage macrolactonization reaction [55]. Their synthesis commenced from isopropylidene-d-erythrono-1,4-lactone **101** as a chiral starting material to intermediate **102** in 10 steps with 27% yield (Scheme 20). TMS-ethyl ester was obtained from the corresponding methyl ester **103** via ester cleavage with TMSOK under microwave conditions followed by Suzuki coupling with **102** gave **104** in 60% yield. Hydrogenation of **104** under Lindlar condition and deprotection gave the seco acids **105** in good yield. Cyclization under Mitsunobu conditions followed by deprotection and oxidation with polymer-bound IBX led to a mixture of L-783277 (**4**) and a second mono-oxidized product whose exact structure was uncertain, separated by flash chromatography.

**Scheme 21:**
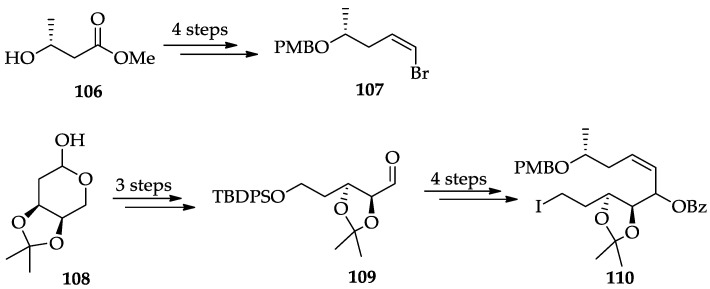
Synthesis of intermediates of L783277 (**4**) by Winssinger et al. [47].

In 2009, the Winssinger Group reported the synthesis of L-783277 (**4**) together with the synthesis of LL-Z1640-2 (**3**), and hypothemycin (**2**) as a part of their divergent synthetic approach towards resorcylic acid lactones [47]. According to their synthetic route, vinyl bromide **107** could be obtained in 4 steps from the less expensive methyl 3-hydroxybutyrate **106** (Scheme 21). Another commercially available protected deoxyribose **108** was converted to protected aldehyde **109** in 3 steps which was further converted to intermediate **110** in 4 synthetic steps with 72% yield. 

**Scheme 22:**
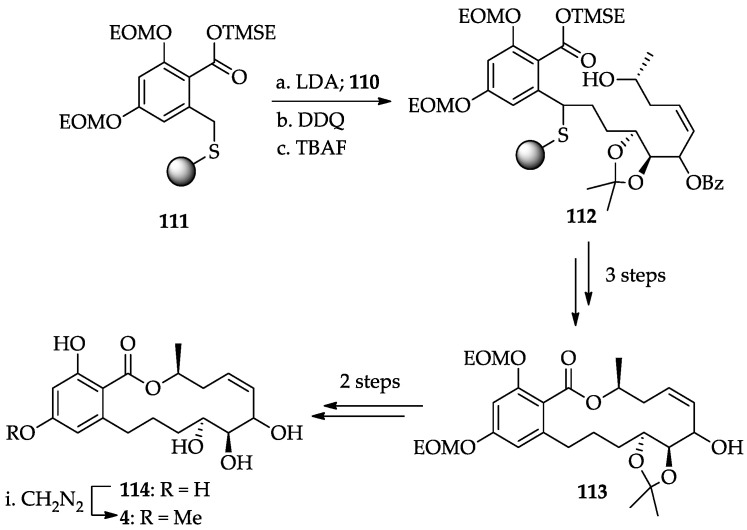
Synthesis of L783277 (**4**) by Winssinger et al. [47].

Toluate position of resin-bound **111** was alkylated with iodide **110** to obtain polymer-bound intermediate **112** followed by removal of the PMB and the TMSE. Macrolactonization under Mitsunobu conditions followed by reductive conditions to get macrocycle **113** after benzoate hydrolysis (Scheme 22). The natural products L-783277 (**4**) could be obtained by straightforward diazomethane treatment of compounds **114** (>90% conversion based on LC/MS, 63–74% isolated yield after HPLC purification). It should be noted that their final product isolated by HPLC were typically contaminated with small amounts (>5%) of a side product that is tentatively ascribed to the *trans*-enone isomer.

**Scheme 23:**
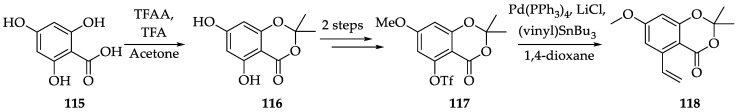
Synthesis of fragment **118** by Sim et al. [53].

In 2010, our group successfully accomplished efficient and enantioselective total synthesis of L-783277 (**4**), based on convergent assembly of one aromatic unit and two chiral building blocks with efficient orthogonal protection-deprotection strategy [53]. Three key steps composed of olefin cross metathesis, addition of acetylene derivative to aldehyde, and Yamaguchi macrolactonization were subsequently employed to construct the framework of L-783277 (**4**) (Scheme 23). The fragment **118** was prepared from commercially available 2,4,6-trihydroxybenzoic acid **115** in 4 steps with 23% yield. Trihydroxybenzoic acid **115** was treated with TFA and TFAA in acetone to afford the corresponding acetonide **116** on the basis of modified Danishefsky’s method which after two-step protection delivered **117** and finally Stille coupling reaction to furnish styrene **118** was obtained. 

**Scheme 24:**
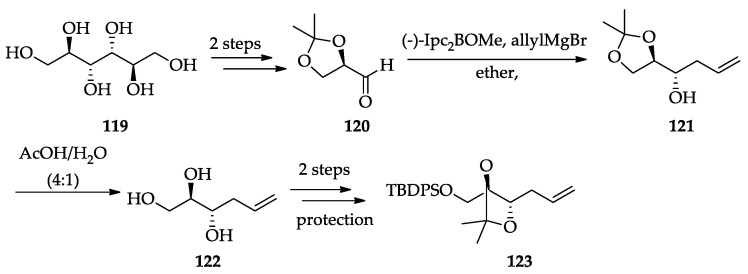
Synthesis of fragment **123** by Sim et al. [53].

D-(*R*)-glyceraldehyde acetonide **120** was prepared from D-mannitol **119** in two steps according to the literature method [63]. The asymmetric Brown allylation of aldehyde **120** using (−)-Ipc_2_BOMe yielded the desired allylic alcohol **121** in high diastereoselectivity (92:8) (Scheme 24). The deprotection of the acetonide group generated **122** and protection of TBDPS and acetonide then produced fragment **123** in 38% yield from glyceraldehyde.

**Scheme 25:**
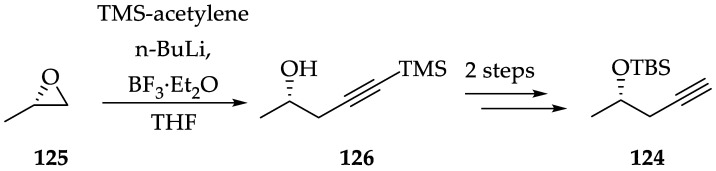
Synthesis of fragment **124** by Sim et al. [53].

The preparation of the third alkyne fragment **124**, commenced with commercially available (*S*)-(−)-propylene oxide **125**, which underwent ready conversion into secondary alcohol **126** upon treatment with lithium trimethylsilylacetylene (Scheme 25). Protection and deprotection gave us alkyne fragment **124** with a 93% yield in 2 steps. 

**Scheme 26:**
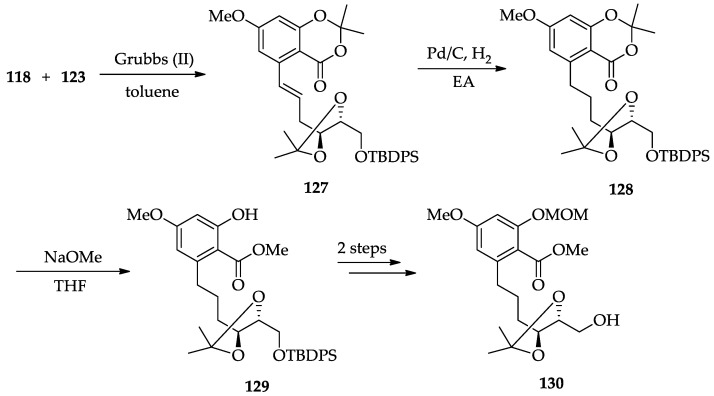
Synthesis of fragment **130** by Sim et al. [53].

Olefin cross metathesis strategy between fragments **118** and **123** using Grubbs 2nd generation catalyst furnished the styrene **127** (*cis*:*trans* = 3:7) which hydrogenated to get **128** (Scheme 26). The transesterification of the compound was conducted with NaOMe to provide methyl ester **129**, which after protection and deprotection generated primary alcohol **130** with 17% yield in 5 steps. 

**Scheme 27:**
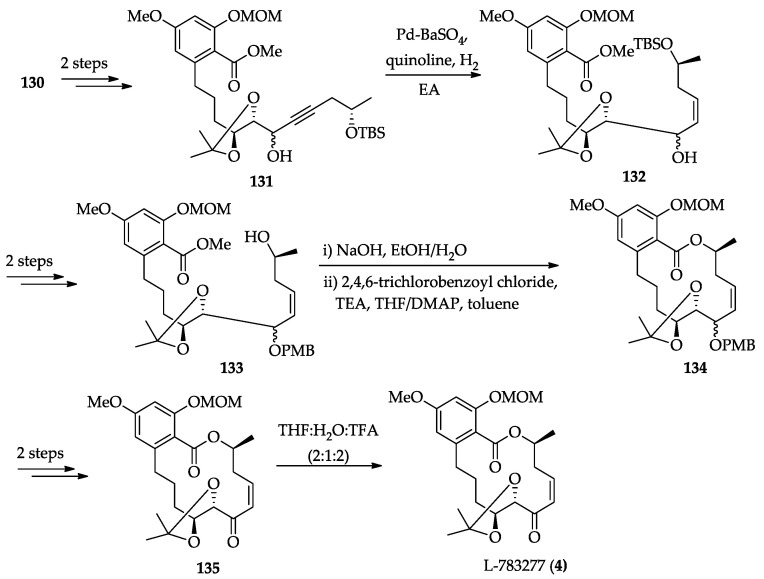
Synthesis of L-783277 (**4**) by Sim et al. [53].

The primary alcohol **130** was smoothly oxidized to the corresponding aldehyde and attacked by lithium acetylide to afford allylic alcohol **131** in 42% yield over two steps (Scheme 27). Hydrogenation reaction using Lindlar catalyst to give the corresponding *cis*-olefin **132**, which after protection and deprotection generate **133** with 86% yield in 3 steps. Saponification followed by cyclization under Mukaiyama condition generated **134** in moderate yield. Finally, deprotection followed by oxidation and global deprotection furnished natural product L-783277 (**4**) with 63% yield in 3 steps.

**Scheme 28:**
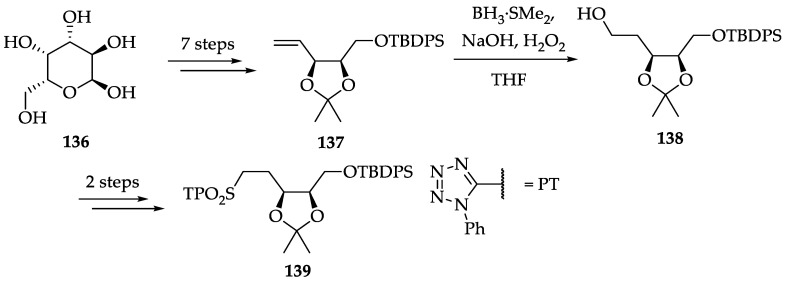
Synthesis of fragment **139** by Nanda et al. [64].

Recently, The Nanda Group reported an asymmetric total synthesis of L-783277 (**4**) through intramolecular base-mediated macrolactonization reaction [64]. The synthesis was initiated with D-galactose **136**, which after 7 synthetic steps gave **137** in 40% yield and further reduction generate **138** in 80% yield (Scheme 28). Their desired sulfone **139** for Julia precursor can be obtained from **138** in 2 steps with 85% yield. 

**Scheme 29:**
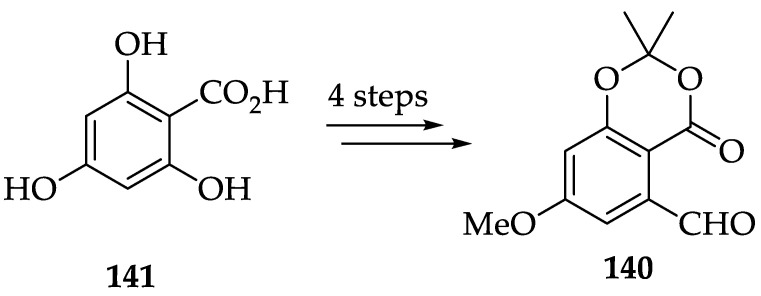
Synthesis of fragment **140** by Nanda et al. [64].

The aromatic fragment **140** can be prepared in 4 steps starting from 2,4,6-trihydroxy benzoic acid **141** as starting material (Scheme 29). 

**Scheme 30:**
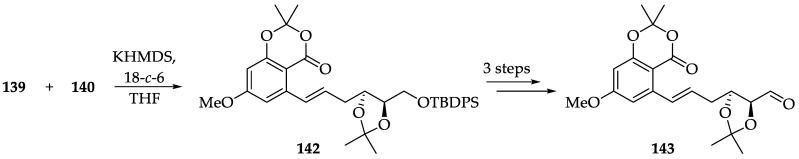
Synthesis of intermediate **143** by Nanda et al. [64].

Sulfone **139** and aromatic aldehyde **140** was then subjected to JK olefination to furnish the ‘*E*’-olefin **142** in 78% yield which after 3 steps gave aldehyde intermediate **143** with 73% yield (Scheme 30). 

**Scheme 31:**
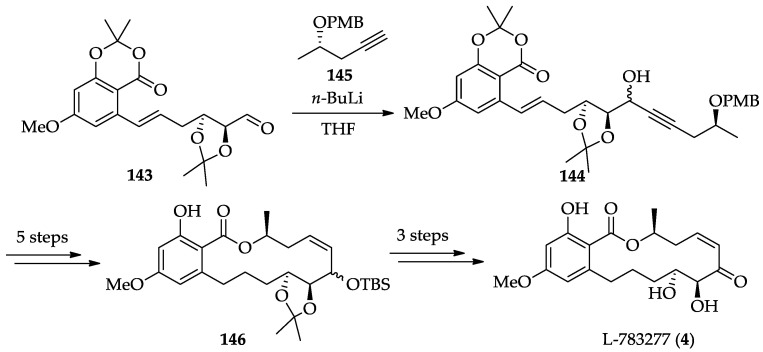
Complete synthesis of L-783277 (**4**) by Nanda et al. [64].

Alkyne **144** which was obtained through nucleophilic addition reaction of **143** with alkyne **145**, after 5 steps gave **146** with 45% yield in 5 steps. Deprotection and oxidation then furnished natural product L-783277 (**4**) with 47% yield in 3 steps (Scheme 31).

**Figure 7:**
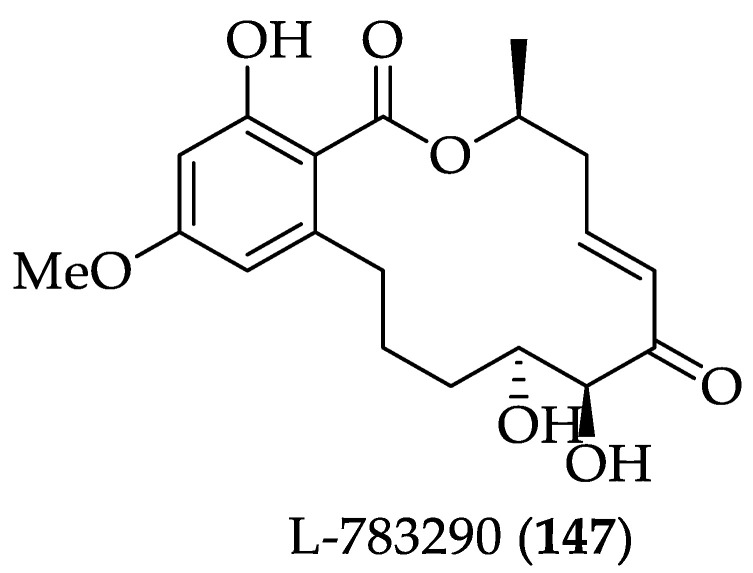
Structure of L-783290 (**147**).

A *trans*-isomer of L-783277 (**4**) was also isolated but RALs bearing the *cis*-enone moiety such as hypothemycin (**2**), LL-Z1640-2 (**3**), and L-783277 (**4**) are more potent than the *trans*-enone L-783290 (**147**) (Figure 7). Banwell’s group reported the first synthesis of L-783290 (**147**, the *E*-counterpart of L-783277 (**4**), Figure 6) through the exploitation of Heck coupling and intramolecular Weinreb ketone synthesis followed by other synthetic manipulation [65].

**Scheme 32:**
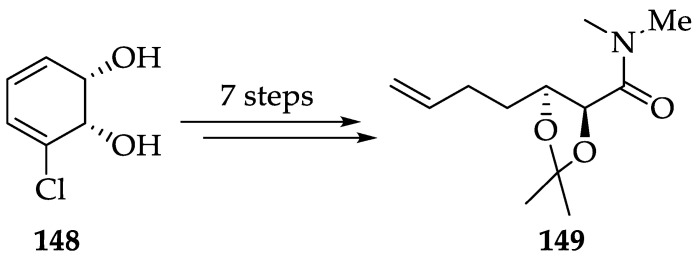
Synthesis of fragment **149** by Banwell et al. [65].

A chemoenzymatic pathway was employed where they used enantiomerically pure and commercially available *cis*-1,2-dihydrocatechol **148** derived from the whole-cell biotransformation of chlorobenzene to get Weinreb amide fragment **149** in 7 steps (Scheme 32). 

**Scheme 33:**
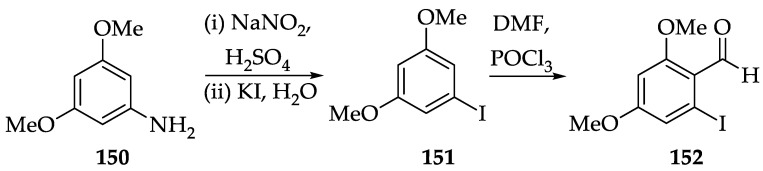
Synthesis of Fragment **152** by Banwell et al. [65].

The reaction of commercially available dimethoxyaniline **150** to a Sandmeyer reaction followed by Vilsmeier–Haack conditions afforded benzaldehyde **152** in 2 steps with 44% yield (Scheme 33). 

**Scheme 34:**
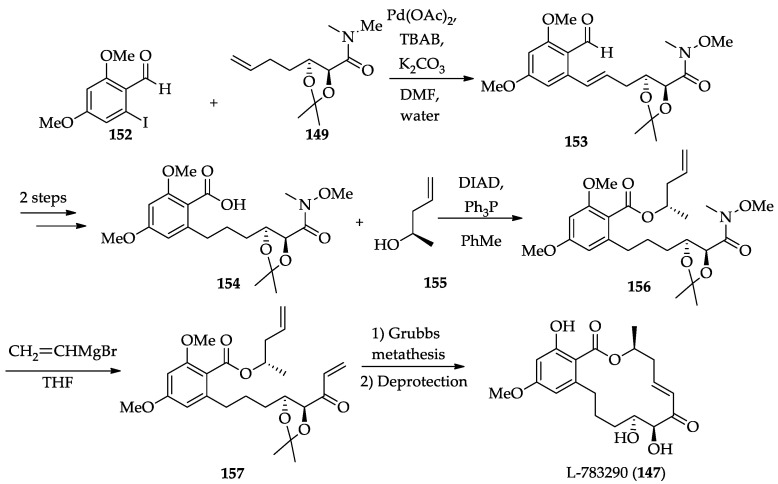
Synthesis of L-783290 (**147**) by Banwell et al. [65].

The Heck coupling reaction of aryl iodide **152** and terminal alkene **149** gave **153**, which after 2 steps produced **154** for a Mitsunobu reaction with **155** to give ester/amide **156**, which was treated with Grignard reagent to get **157**. Finally, Grubbs metathesis and deprotection furnished target macrolide **147** in 10% yield (Scheme 34).

5′-Deoxy analog (**158**) of L-783277 (**4**) was synthesized stereoselectively in 2011 by Altmann group [66]. This analog retains almost the full kinase inhibitory potential of natural L-783277 (**4**), with low nanomolar IC_50_ values against the most sensitive kinases, and it exhibits essentially the same selectivity profile (within the panel of 39 kinases investigated). In contrast, removal of both the 4′- and the 5′-hydroxyl groups leads to a more significant reduction in kinase inhibitory activity.

**Scheme 35:**
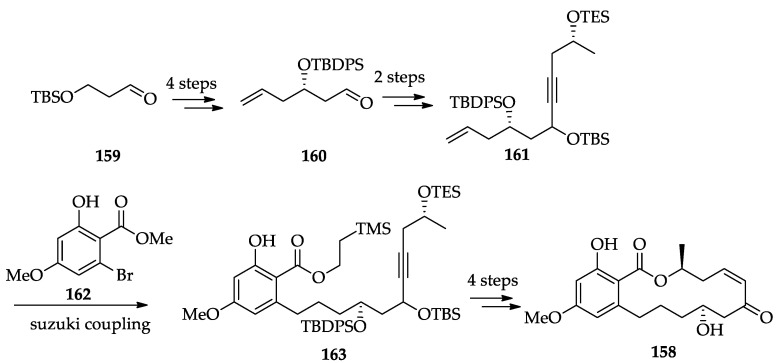
Synthesis of 5′-deoxy analogue (**158**) by Altmann et al. [66].

Intermediate **160** was prepared from TBS-protected 3-hydroxy propanal **159** in a high-yielding four-step sequence (69% overall yield) involving Keck allylation (Scheme 35). After some synthetic steps, intermediate **160** was converted to **161**, which was coupled with aromatic fragment **162** by Suzuki coupling to get **163**. Mitsunobu cyclization, protection, deprotection steps were used to furnished 5′-deoxy analog (**158**) of L-783277 (**4**) in 4 steps. 

**Scheme 36:**
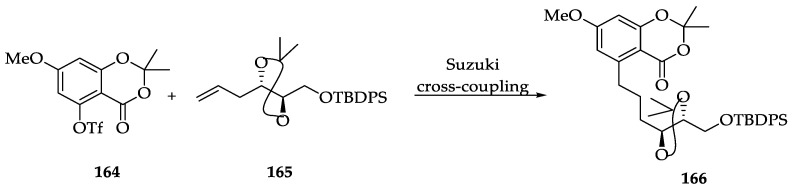
Synthesis of key intermediate **166** by Sim et al. [52].

Our group synthesized a saturated analog (**99**) of L-783277 (**4**) [52]. Our synthetic route comprises three key steps involving a Suzuki cross-coupling reaction between an aromatic triflate and olefin, the addition of an acetylene to an aldehyde, and Mitsunobu macrolactonization (Scheme 36). Triflate **164** and olefin **165**, prepared from commercially available 2,4,6-trihydroxybenzoic acid and 1,2,5,6-bis-*O*-(1-methylethylidene)-d-mannitol, respectively (structures not shown), would be joined by the use of a Suzuki cross-coupling reaction to produce the key intermediate **166**. 

**Scheme 37:**
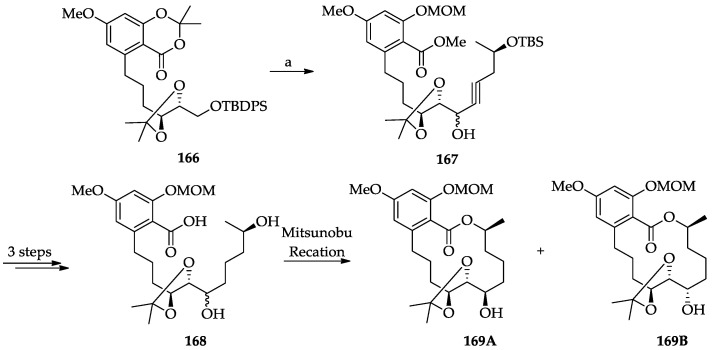
Synthesis of intermediates **169A** and **169B** by Sim et al. [52].

The propargylic alcohol **167** was prepared from **166** using the same sequence of reactions described in our previous report [53]. Lindlar hydrogenation, deprotection, and saponification generated seco acid **168**, which was cyclized under Mitsunobu condition to deliver a diastereomeric mixture of **169** (Scheme 37).

**Scheme 38:**
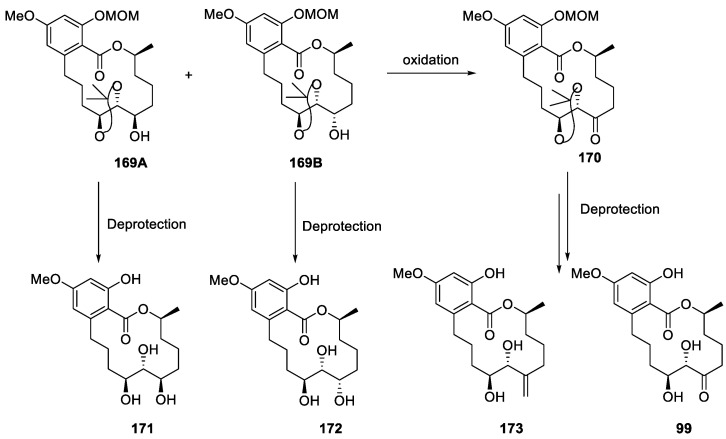
Synthesis of analogs (**99, 172**–**173**) from intermediates **169A** and **169B** by Sim et al. [52].

Oxidation of a mixture **169** gave pure keto derivative which after final deprotection furnished target lactone **99** (Scheme 38). It is noteworthy to mention that advanced intermediates **169** and **170** in the route for synthesis of **99** were also used for preparation of the trihydroxy analogs of L-783277 (**4**), **171**, **172,** and external olefin containing analog **173** (Scheme 38).

Next, we synthesized phenyl ring incorporated derivatives of L-783277 (**4**) in expectation that these derivatives would improve kinome-wide selectivity of the parent compound **4** [54]. The key reactions employed in the synthetic sequences are Suzuki coupling, Sharpless asymmetric dihydroxylation, alkyne addition to an aldehyde, Lindlar reduction, and Mitsunobu cyclization. 

**Scheme 39:**
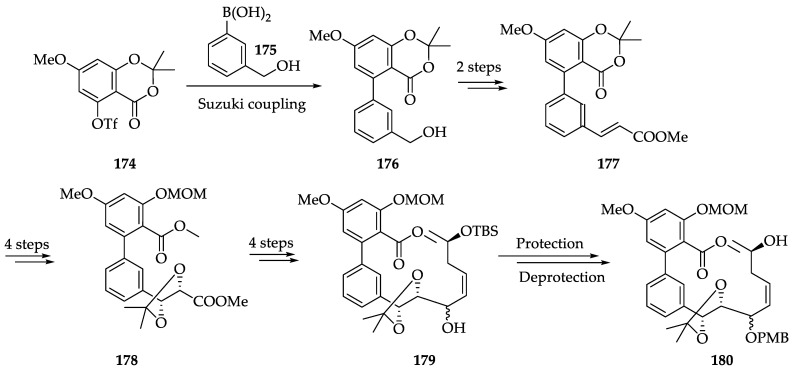
Synthesis of the cyclization precursor **180** by Sim et al. [54].

Our route began with Suzuki coupling between triflate **174** and boronic acid **175** to produce biphenyl derivative **176** firstly (Scheme 39). Oxidation followed by Wittig olefination formed *trans* form of **177**. Sharpless asymmetric dihydroxylation followed by protection and *trans*-esterification produced **178** in 4 steps which after 4 synthetic steps produced **179**. PMB-ether protection followed by TBS-ether deprotection then generated **180** in 2 steps, the important cyclization precursor. 

**Scheme 40:**
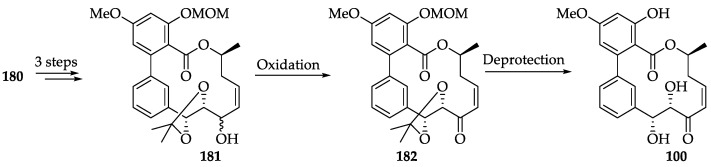
Synthesis of rigidified analogue of L-783277 (**100**) by Sim et al. [54].

Subsequently, **180** underwent saponification followed by cyclization under Mitsunobu condition and deprotection to get **181** in 3 synthetic steps with good yield (Scheme 40). Oxidation of **181** formed the corresponding ketones **182** as a single stereoisomer, which was finally deprotected to form target **100**. Other derivatives also we synthesized by using a similar synthetic strategy to expand the scope of the SAR study.

### 2.2. Sesquiterpene Lactones

**Figure 8:**
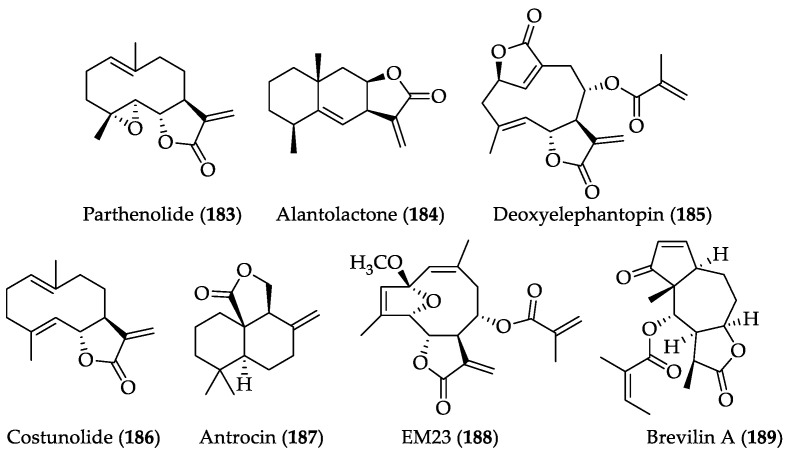
Structures of representative sesquiterpene lactones.

Sesquiterpene lactones (SLs) are a sub-class of sesquiterpenoids, which are natural products synthesized in several organs of plants such as leaves, shoots, and roots [67,68]. In addition, SLs comprise a large and diverse group of secondary metabolites isolated from diverse plant families [69,70] with the greatest number occurring in members of Asteraceae [69,70,71]. Extracts of SLs, like other natural products, have been broadly used in folk medical practices for fever, headache, rheumatoid arthritis, and menstrual irregularities [72]. SLs are usually characterized by the presence of *α*-methylene-*γ*-lactone group (*α*M*γ*L), which mainly contributes to the underlying biological activity of SLs through irreversibly forming a Michael adduct with nucleophilic amino acid residues in proteins, causing alterations in protein activity [73]. This is regarded as the primary mechanism of cytotoxicity induced by SLs (Figure 8).

#### 2.2.1. Biological Activities of Parthenolide (**183**)

Parthenolide (**183**) is a naturally occurring sesquiterpene lactone derived from Tanacetum parthenium, also known as feverfew [74]. It is regarded as a recognized candidate for drug development due to notable anti-cancer and anti-inflammatory properties. Parthenolide (**183**) contains an *α*-methylene-*γ*-butyrolactone group and an epoxide group, which participate in interactions with protein targets to exert multiple therapeutic effects [75]. To date, parthenolide (**183**) has been reported to vitiate cancer pathogenicity in various types of cancer including myeloma, colorectal, liver prostate, pancreatic, thyroid, and breast cancers [76,77,78,79]. Especially, parthenolide (**183**) showed effects on suppression of MDA-MB-231 breast cancer cells and reduction in tumor size in a mouse xenograft model, when it was used in combination with docetaxel [80]. Interested by cytotoxicitiy of parthenolide (**183**) across a wide range of human cancers and its high tolerability in humans [81], several researches focused on the identification of molecular targets of parthenolide (**8**). Kwok et al., in 2001, reported that one of the primary targets that induces the anti-cancer activity is IκB kinase *β* (IKK-*β*) wherein both IKK-*β* and nuclear factor *κ*B (NF-κB) signaling are impaired due to modified cysteine 179 [82]. In addition, more findings indicate that parthenolide affects additional cell signaling pathways such as induction of oxidative stress and apoptosis, focal adhesion kinase 1 (FAK1) signaling, mitogen-activated protein kinase signaling, and mitochondrial function [82,83,84,85,86]. Recently, Berdan et al. reported that parthenolide (**183**) shows inhibitory activity on FAK1 signaling pathway by targeting cysteine 427 of FAK1 [87].

**Figure 9:**
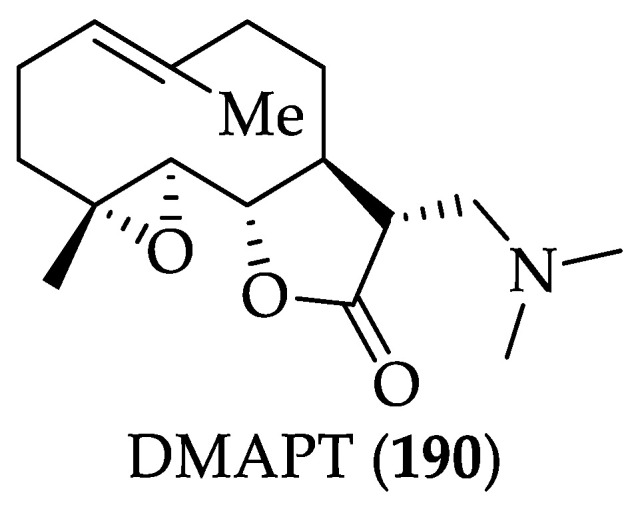
Structure of dimethylamino parthenolide, DMAPT (**190**).

However, parthenolide (**183**) is quite lipophilic in nature and, accordingly, poor bioavailability caused by low solubility has risen as a critical issue. Therefore, a dimethylamino group containing derivative of parthenolide, DMAPT (**190**) (Figure 9), was developed to overcome poor solubility of parthenolide (**183**) without losing the original anti-tumor activity [88]. The hydrophilic parthenolide analog, (**190**), did not only increase pharmacokinetic properties compared to the parent compound, but it was also able to selectively eliminate acute myeloid leukemia (AML) stem cells, which led to the initiation of a clinical trial for its use in hematologic cancer [89].

#### 2.2.2. Chemistry of Parthenolide (**183**)

**Scheme 41:**
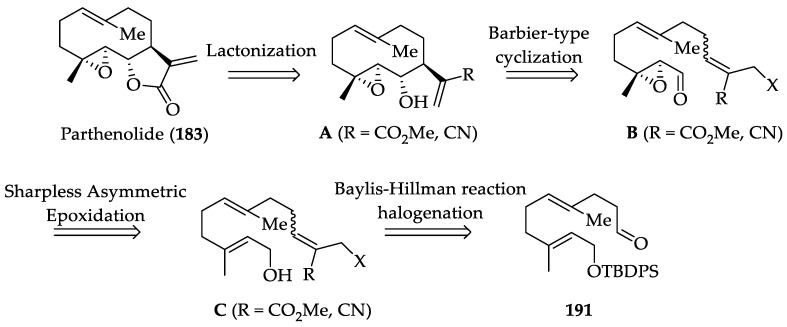
Retrosynthesis of parthenolide (**183**) according to Long et al. [90].

The first total synthesis of parthenolide (**183**) and its analogs was reported by Long and co-workers in 2014 [90]. In their initial retro-synthetic analysis of parthenolide (**183**) (Scheme 41), it was anticipated that parthenolide (**183**) could be obtained by the known compound **191** through three key intermediates. The crucial reaction step conceived by Long et al. for the target molecule included intramolecular Barbier-type cyclization of **B** to form **A** [91], which then undergoes lactonization to generate parthenolide (**183**).

**Scheme 42:**
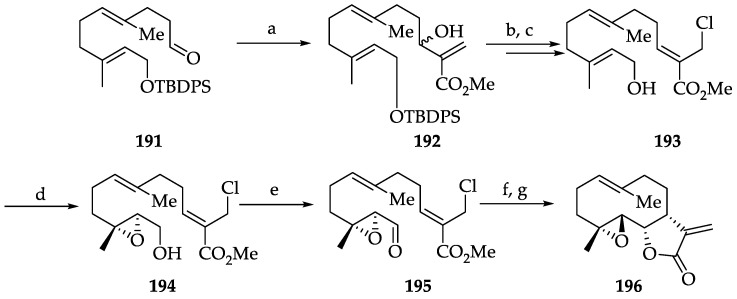
Synthesis of intermediate **196** by Long et al. [90]. Reagents and conditions: (a) methyl acrylate, DABCO, RT, 77%; (b) CCl_4_, *n*-Bu_3_P, 83%; (c) HF-pyridine, THF, 91%; (d) 4 Å MS, Ti(O*i*Pr)_4_ (0.1 equiv), (–)-DIPT (0.12 equiv), TBHP (1.5 equiv), CH_2_Cl_2_, −40 °C to −18 °C, 93%, ee = 92%; (e) Dess–Martin periodinane, NaHCO_3_, CH_2_Cl_2_, 92%; (f) CrCl_2_, DMF; (g) DBU, CH_2_Cl_2_, 41% over 2 steps.

The primary attempt of the group to synthesize parthenolide (**183**) is summarized in Scheme 42. The synthesis was initiated with known compound **191**, which was obtained from farnesol in three steps [92]. **191** was treated with methyl acrylate and 1,4-diazabicyclo[2.2.2]octane (DABCO) to produce substituted acrylate **192**. This was followed by chlorination with simultaneous double bond isomerization to provide only (*Z*)-**193** and TBDPS was deprotected by using HF-pyridine. It should be noted that TDBPDS deprotection of **193** did not proceed under standard TBAF or TFA conditions [90]. The primary alcohol of **193** underwent Sharpless asymmetric oxidation (SAE) reaction to yield compound **194**, which was subsequently oxidized to give corresponding aldehyde **195**. Authors have investigated the cyclization of **195** by means of reductive Barbier-type coupling conditions to form the desired 10-membered ring. Under CrCl_2_ in degassed DMF, amongst other conditions, such as Zn^0^, In^0^, and SmI_2_, only gave out the cyclized product **196**. Unfortunately, the lactone **196** was revealed to be the C-7 epimer of parthenolide (**183**) after an analysis of X-ray co-crystal data [90].

**Scheme 43:**
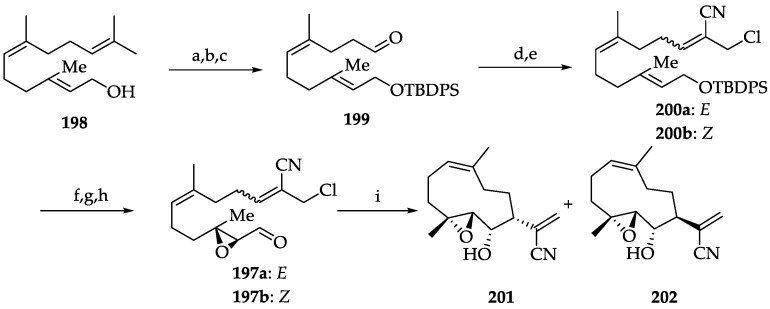
Synthesis of *trans*-intermediate **202** by Long et al. Reagents and conditions: (a) TBDPSCl, imidazole; (b) NBS, THF/H_2_O, then K_2_CO_3_, MeOH; (c) H_5_IO_6_, NaIO_4_, 63% over 3 steps; (d) acrylonitrile, DABCO, RT; (e) CCl_4_, *n*-Bu_3_P, 73% over 2 steps, 27a/b = 3:1; (f) HF-Pyridine, THF; (g) 4 Å MS, Ti(OiPr)_4_ (0.1 equiv), (–)-DIPT (0.12 equiv), TBHP (1.5 equiv), CH_2_Cl_2_, −40 °C to −18 °C; (h) Dess–Martin periodinane, NaHCO_3_, CH_2_Cl_2_, for 28a 3 steps, 81%, ee = 97%, for 28b 3 steps, 76%, ee = 95%; (i) NaI, acetone (b) CrCl_2_, THF, rt [90].

Long et al. have designed and synthesized cyclization precursors **197a** and **197b** via 8 steps, based on their speculation that the configuration of the two new stereogenic centers might be highly affected by the geometric configuration of 1,10-double bond (Scheme 43) [90]. From nerol, compound **198** was obtained in 5 steps [93,94]. After protecting alcohol of **198** with TBDPS, C10-C11 double bond was cleaved to afford aldehyde **199**, which underwent Baylis–Hillman reaction, then chlorination to produce **200a** and **200b** in 3:1 ratio. Lastly, **200a** and **200b** were used to obtain the cyclization precursors **197a** and **197b**. A reaction condition of an umpolung allylation optimized by Baran et al. was utilized in the transformation of **197a** to 6,7-*cis* **201** and the desired 6,7-*trans* **202** in a ratio of 2.8:1 in 16% yield [95]. Surprisingly, the ratio of 6,7-*trans* **202** reaction yield was much improved when the relatively low reactive allylic chloride was converted into allylic iodide prior to Barbier reaction using CrCl_2_ in THF. This two-steps procedure produced an increased ratio of 6,7-*trans* **202** (1:1) and a moderate yield (52%) [90].

**Scheme 44:**
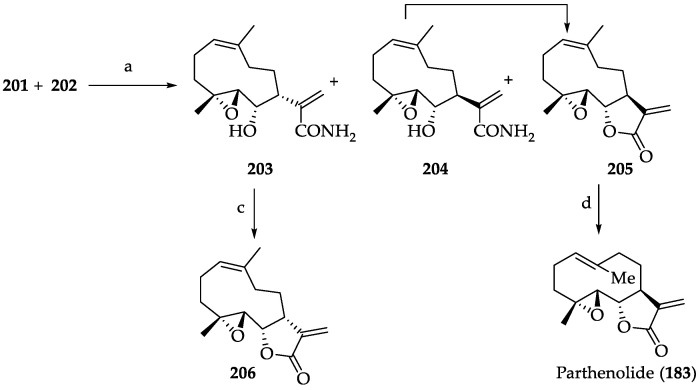
Synthesis parthenolide (**183**) and its analog (**206**). Reagents and conditions: (a) K_2_CO_3_, H_2_O_2_, DMSO/THF, 86%; (b) DBU, CH_2_Cl_2_, rt, 92%; (c) DBU, benzene, reflux, 91%; (d) *hv* (254 nm), benzene, conversion: 58%, yield: 77% based on recovered starting material [90].

Scheme 44 provided the target compound parthenolide (**183**). Hydrolysis of **201** and **202** by using basic hydrogen peroxide produced compounds **203**, **204**, and **205** (Scheme 44). Lactone **206** was obtained by refluxing compound **203** in benzene with DBU. Moreover, another side product **204** could be converted to the desired intermediate **205** upon stirring with DBU in CH_2_Cl_2_. The target molecule **183** was successfully obtained upon irradiating **205** with UV light (254 nM) in 58% conversion and 77% yield [90].

Overall, Long et al. have demonstrated the first effective synthetic route, which can be used to synthesize parthenolide (**183**) and its analogs. Moreover, insights acquired from this investigation on synthetic routes of parthenolide (**183**) may provide general strategies to synthesize *trans*-germacronolide analogs with high medical relevance.

**Scheme 45:**
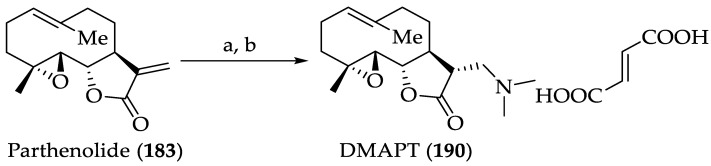
Synthesis of dimethylamino-parthenolide analog (**190**). Reagents and conditions: (a) dimethylamine, MeOH, rt; (b) Fumaric acid, diethyl ether [88].

In an effort to overcome poor water solubility of parthenolide (**183**) without compromising its anti-leukemic activity, Neelakantan and coworkers carried out the synthesis and structure-activity relationship studies of a series of aminoparthenolide analogs derivied from various aliphatic primary and secondary amines [88]. It is of significance that *R*-configuration at the newly formed C-11 chiral center was conserved after the Michael-type addition of parthenolide (**183**) with aliphatic amines (Scheme 45). Authors have concluded that the protonation of the enolate formed during the reaction, which occurs from the *exo* face of the molecule, causes the conservation of *R*-conformation at C-11 in the product. During the course of investigation, dimethylamino analog (**190**) was identified as the representative compound exhibiting significant anti-leukemic activity in the AML assay [88]. However, aminoparthenolide analogs showed low metabolic and chemical stability. Especially, it was prone to the cytochrome P450 (CYP450)-catalyzed oxidation, which significantly decreases the bioactivity of the compound [96]. Therefore, additional research to reduce the effect of CYP450-catalyzed oxidation was necessitated.

**Figure 10:**
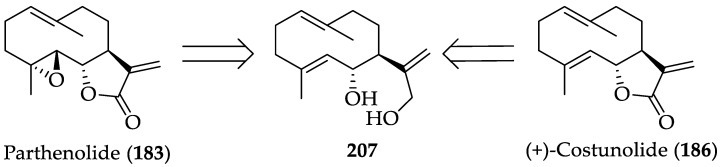
Preparation of parthenolide (**183**) and costunolide (**186**) from **207** [97].

In addition, Yang et al. reported the asymmetric total synthesis of germacrane ring **207**, inspired by the biosynthetic route of sesquiterpene lactones (Figure 10) [97]. A more detailed description on the synthetic route used to afford the germacrane ring **207** will be provided in the chemistry of costunolide (**186**) section (see Section 2.2.8). The key intermediate **207** can be served as a great starting point to prepare the natural lactones parthenlide (**183**) and costunolide (**186**).

**Scheme 46:**
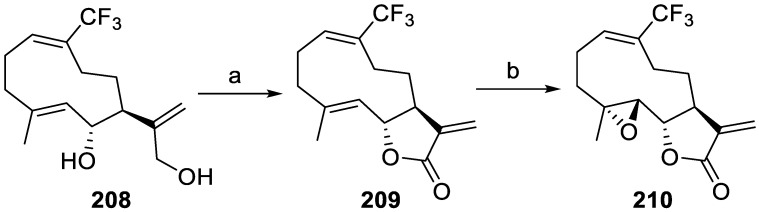
Synthesis of trifluoromethylated analogs (**209** and **210**) from **208.** Reagents and conditions: (a) MnO_2_, CH_2_Cl_2_, r.t., 8 h, 25% over 3 steps; (b) *m*-CPBA, CH_2_Cl_2_, 25 °C, 3 h, 91%. [97].

Moreover, trifluoromethylated germacrane skeleton **208** can be used to synthesize analogs of the aforementioned lactones, such as trifluoromethylated analogs (**209** and **210**) (Scheme 46). Based on literature findings [98,99,100], authors expected that replacing 14-methyl group with an electron-withdrawing group such as a trifluoromethyl moiety would protect against oxidation by CYP450 oxidases. Both analogues showed a comparable anti-cancer activity to their natural product counterparts, parthenolide (**183**) and costunolide (**186**), respectively. Moreover, **210** was less acid-labile than parthenolide (**183**) and exhibited metabolic stability equivalent to that of parthenolide (**183**) [97].

#### 2.2.3. Biological Activities of Alantolactone (**184**)

Alantolactone (**184**) is a potent anticancer compound for various tumor cells, which is mainly obtained from the medical herb *Inula helenium* [101,102,103]. However, the underlying mechanism of anti-cancer activity of alantolactone (**184**) is still not clearly determined. Liu et al. investigated antiangiogenic effect alantolactone and its molecular mechanism. Alantolactone (**184**) exhibits an IC_50_ value of 40 μM against MDA-MB-231 cells and 14.2 μM against human umbilical vein endothelial cells (HUVEC) cells. This indicates selective nature of alantollactone (**184**) on active endothelial cells. VEGFR2 phosphorylation plays a key role in tumor angiogenesis and authors demonstrated alantolactone (**184**) suppresses VEGFR2 phosphorylation and its downstream signals, including PLC*γ*1, FAK, Src, and AKT [104]. Hence, alantolactone (**184**) can work as a VEGFR2 inhibitor and exhibit antiangiogenic activity. Xing Kang et al. suggested in their study of alantolactone (**184**) on HepG2 (description) that ROS-AKT pathway might participate in apoptosis induced by alantolactone (**184**) [105]. In addition, alantolactone (**184**) can penetrate the blood brain barrier (BBB) and act as a therapeutic agent for CNS neoplasm. Alantolactone (**184**) induces apoptosis in glioblastoma cells by inhibiting NF*k*B/COX-2 signaling pathway. Expression of COX-2 in glioblastoma cells was suppressed mainly due to inhibition of IKKβ by binding of alantolactone (**184**) to the ATP-site [106].

#### 2.2.4. Chemistry of Alantolactone (**184**)

The stereoselective total synthesis of racemic alantolactone (**184**) was reported in 1965 by Marshall et al. [107]. To the best of our knowledge, there were no other reports illustrating the enhanced synthetic approaches to alantolactone (**184**), but a number of groups have reported semi-synthetic derivatives of the natural product and their biological evaluation. Probably, this is because there are abundant available sources of alantolactone in nature, including *Inula helenium* L., *Inula japonica*, *Aucklandia lappa*, *Inula racemosa*, and so on, where it can be isolated at ease by means of different chromatographic techniques [108,109,110,111].

**Scheme 47:**
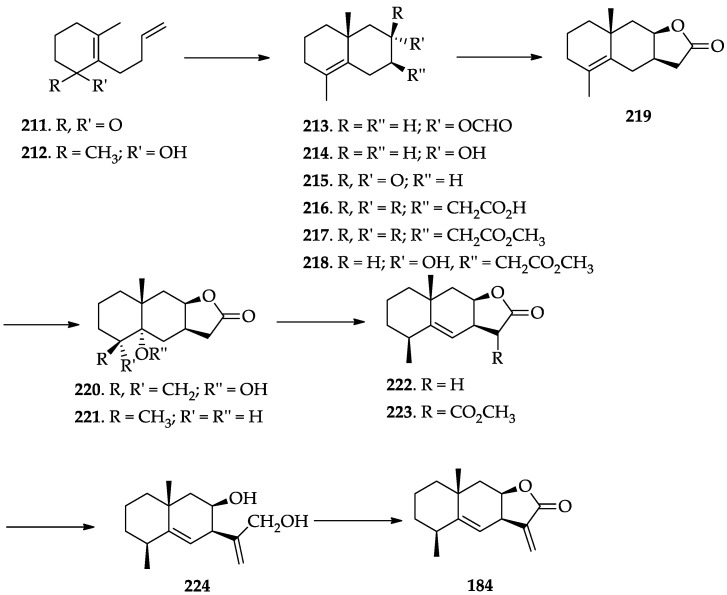
Total synthesis of alantolactone (**184**) according to Marshall et al. [107].

Three key reaction steps categorized by Marshall and coworkers are as follows: first, the construction of the carboxylic scaffold by cationic olefin cyclization (**212** to **213**), secondly, modification of the carbon framework to afford **222**, and lastly, formation of *α*-methylene-*γ*-butyrolactone (**222** to **184**). The actual synthesis of Alantolactone (**184**) is summarized in Scheme 47, commencing with unsaturated ketone **211** afforded from 4-bromobutene in two steps [107]. Ketone **211** then was reacted with ethereal methylithium to give alcohol **212**, which was cyclized to generate formate **213** according to a procedure previously developed by Johnson and co-workers for analogous compounds [112]. Alcohol **214** produced from saponification of the resulting formate **213** was oxidized with chromic acid reagent giving octalone **215** [113]. Upon treatment of ethyl bromacetate to **215** via Stork enamine procedure and saponification of the crude mixture, keto acid **216** was afforded in 53% overall yield [107]. It should be noted that steric effects around C-9 rendered the alkylation difficult and thus formed Δ^7^-isomer more predominantly than Δ^7^-isomer, which eventually led to the formation of C-alkylation product **216** in high yield [114]. Keto acid **216** underwent esterification to furnish, after reduction with methanolic potassium borohydride, unsaturated lactone **219**. Photo-oxygenation of **219** as stated in the procedure by Nickon and Bagli gave hydroperoxide **220** [115], which was directly reduced to alcohol **221** due to stability issue [107]. The key intermediate **222** was obtained by unsaturation of the alcohol **221** upon treatment with thionyl chloride in pyridine according to the procedure described by Benesova et al. [116]. Lactone **222** was converted to lactone ester **223** via carbomethoxylation with sodium hydride in dimethyl carbonate. **223** was then reduced without further purification to yield diol **224**. Finally, diol **224** was subjected to oxidation with manganese dioxide in benzene to afford racemic alantolactone (**184**). The stereoselective total synthesis of racemic alantolactone (**184**) elucidated by Marshall and co-workers provided an approach to related sesquiterpene lactones [107].

**Scheme 48:**
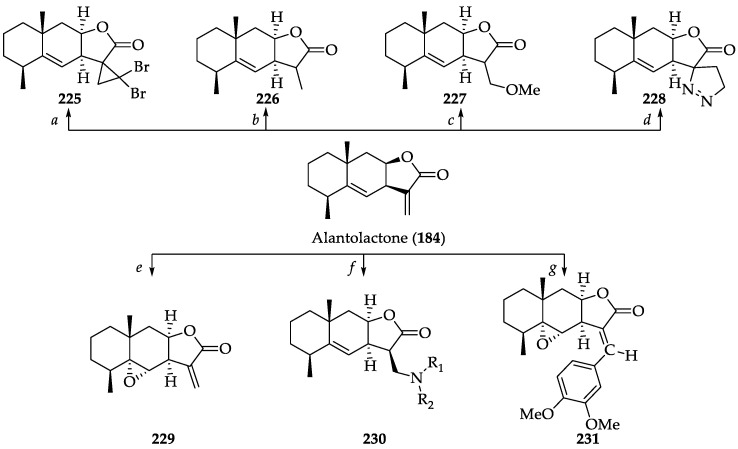
Synthetic transformations of alantolatone (**184**). Reagents and conditions: (a) CHBr_3_, 50% aq. KOH, TEBAC, 5 h; (b) NaBH_4_, MeOH, 25 min; (c) Mg, dry MeOH, 3 h; (d) TEA, diazomethane, overnight; (e) CF_3_CO_3_H, Na_2_CO_3_, DCM, 0 °C; (f); (g) Pd(OAc)_2_/(2-MeC_6_H_4_)_3_, trimethylamine, DMF [117,119,120].

Kaur and co-workers have recently reported a number of semi-synthetic derivatives (**225**–**228**) of the natural product and their biological evaluation [117]. Alantolactone (**184**), upon reaction with diazomethane, afforded pyrazoline derivative **228** as a major product (Scheme 48). Alantolactone (**184**) underwent epoxidation reaction with trifluoroacetic acid in dichloromethane as a solvent to generate compound **229** [118]. Moreover, alantolactone (**184**), like other *α*-methylene-*γ*-lactone moiety containing sesquiterpene lactones, was readily reacted with various amines to yield amine adduct **230** in a stereoselective manner [119]. Shul’ts et al. investigated the pharmacological properties caused by an arylidene fragment in the lactone ring [120]. Thus, aromatic substituents at position C-13 was introduced via Heck reaction with aryl iodides. Compound **231** having (*E*)-configuration was afforded by arylation with iodoarenes in the presence of Pd(OAc)_2_/(2-MeC_6_H_4_)_3_ and trimethylamine in DMF. The configuration of the C(11)=C(13) double bond of **231** was determined by analysis of ^13^C NMR spectrum.

#### 2.2.5. Biological Activities of Deoxyelephantopin (**185**)

Deoxyelephantophin (DET) (**185**) is the major sesquiterpene lactone component present in extracts of medicinal herb *Elephantopus scaber*, which has long been used as a remedy for various types of diseases such as hepatitis, arthritis, asthma, and cancer [121,122]. Recent studies showed that DET exhibits a broad spectrum of antitumor activities including nasopharyngeal [123], cervical [124], colorectal [125], lung [126], skin [127], and particularly in breast cancer [128]. Characteristic structural feature of DET is a functionalized germacranolide skeleton, which is a 10-membered ring with a trans-fused *α*-methylene-*γ*-lactone. It is widely accepted that an *α*, *β*-unsaturated ketones, and an *α*-methylene-*γ*-lactone group of this natural product attribute to a variety of biological effects by acting as a Michael acceptor for nucleophilic amino residues like a cysteine [129].

Although inhibitory activities of DET (**185**) on NK-κB pathways and partial agonistic inhibition of PPAR*γ* were previously described [130,131], covalent targets of DET (**185**) were not clearly assessed. Lagoutte et al. sought to find out direct cellular targets of DET (**185**) with the use of a proteome-wide identification technique. During the investigation, they were able to identify several cellular targets of DET (**185**) (RBP4, GSTA2, SLC26A3, LIPA, and ANXA1) and reported DET (**185**) as the first example of a small molecule that modulates a pharmacologically important nuclear receptor, PPAR*γ* [132,133], activity by engaging a zinc finger through Michael addition [134].

**Figure 11:**
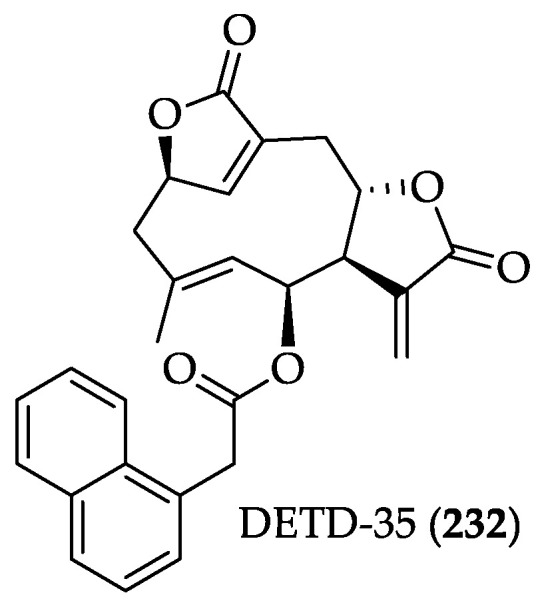
Structure of DETD-35 (**232**).

In addition, Nakagawa-Goto et al. reported the design and synthesis of deoxyelephantophin derivatives (DETDs) and conducted a comprehensive structural-activity relationship study of DETDs on triple-negative breast cancer (TNBC) cell lines. TNBC accounts for around 15% of breast cancers and notorious for having a poor prognosis [135,136]. TNBC, as its name implies, lacks conventional molecular targets usually found in breast cancers namely: estrogen receptor (ER), progesterone receptor (PR), and human epidermal growth receptor2 (HER2). This makes effective treatment for TNBC extremely challenging. Among all derivatives synthesized, DETD-35 (**232**) containing a naphthyl group attached to the ketone by a methylene linker showed the most potent activity and no toxicity towards normal cells in vitro (GI_50_ of DETD-35 (**232**) against MDA-MB-231, a TNBC cell line = 3.5 μM, Figure 11). Moreover, the effects of DET-35 (**232**) on inhibiting cell migration, invasion, and motility of MDA-MB-231 cells were shown to be better than that of the parental compound DET (**185**). Lastly, a combination of DETD-35 (**232**) with a cytotoxic drug, paclitaxel, showed synergistic effects on MDA-MB-231 cells, suggesting that DETD-35 (**232**) could be further developed as a potential compound to complement chemotherapeutic drugs used for TNBC [137].

Besides, in another study carried out by the group, it was demonstrated that DETD-35 (**232**) also shows synergism with other BRAF^V600E^ inhibitors, vemurafenib (PLX4032), to overcome acquired vemurafenib resistant BRAF^V600E^ mutant melanoma in a mouse model [127]. These findings further reinforce the significance of DETD-35 (**232**) as a novel chemical entity for anti-cancer drug discovery campaign.

#### 2.2.6. Chemistry of Deoxyelephantopin (**185**)

**Figure 12:**
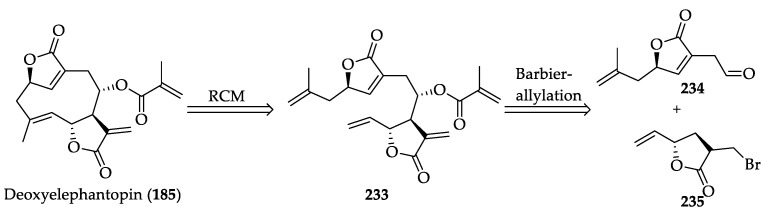
Retrosynthetic analysis of Deoxyelephantopin (**185**) [19].

Deoxyelephantopin has received much interest because of its impressive in vitro and in vivo activities. However, total synthesis of deoxyelephantopin (**185**) has not been reported up-to-date, probably for the same reason as in the case of alantolactone (**184**). Lagoutte et al. profoundly explored the synthesis in regard to deoxyelephantopin (**185**) as part of their endeavor to develop synthetic methodologies to provide analogs of the natural compound, including alkyne tagged probes to be used in target identification [134]. According to the retrosynthetic analysis provided by the group (Figure 12), deoxyelephantopin (**185**) and its analogs were envisioned to be obtained via Barbier-type allylation of fragment **234** and bromolactone **235**, followed by a late-stage ring-closing metathesis (RCM) on substrate **233**. Barbier-type allylation, which serves as the key reaction step in the synthetic route, enables the diastereoselective coupling of the intermediate **235** with diverse aldehydes [19].

**Scheme 49:**
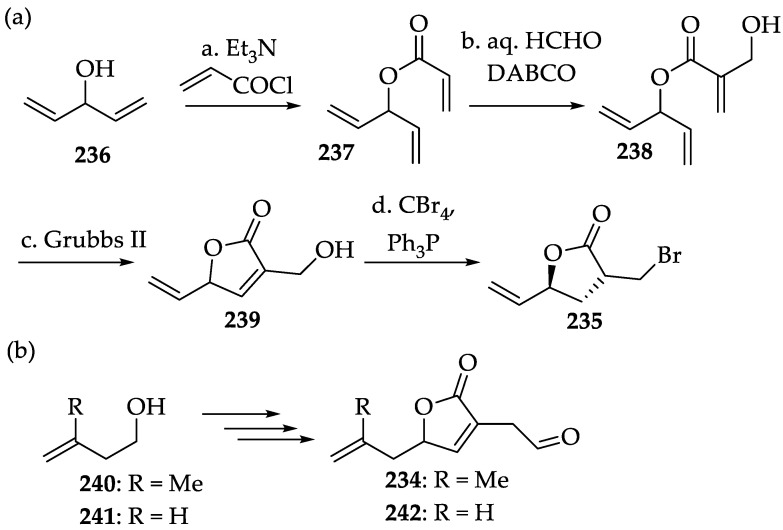
(**a**) Synthesis of bromolactone **235**; (**b**) synthesis of fragment **234** [19].

The synthesis commenced with the esterification of alcohol **236** providing the acrylate **237**, which is subsequently converted to allylic alcohol **238** under Morita-Baylis-Hillman conditions (Scheme 49). RCM of triene **238** mediated by Grubbs II as catalyst generated *endo*-butenolide **239**, which is then converted to key intermediate **235** under Appel conditions. It should be noted that the allylic alcohol of **238** needs to be conserved to accomplish RCM at an appreciable rate [19]. The second fragments (**234**, **242**) required for Barbier-type allylation were prepared in 5 steps from **240** and **241**, respectively.

**Scheme 50:**
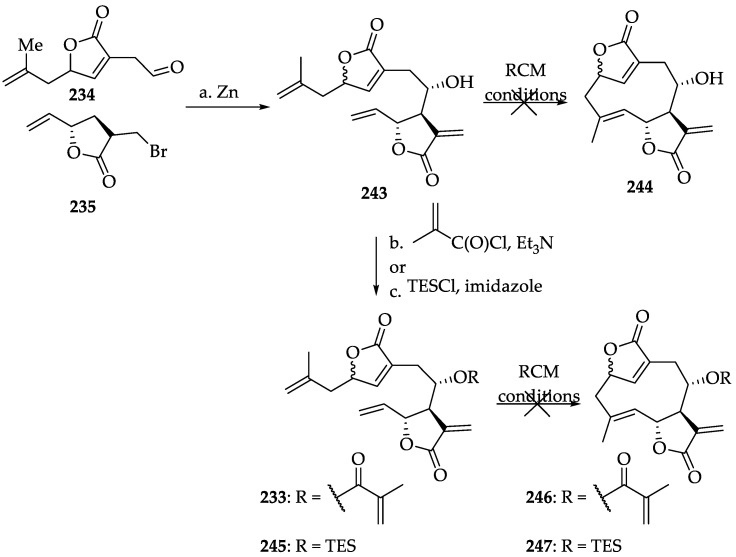
RCM attempts by Lagoutte et al. [19].

As expected, the zinc-mediated Barbier-type allylation of fragments **234** and **235** provided secondary alcohol **243** in moderate yield [138]. However, the resulting alcohol **243** did not undergo RCM to produce the cyclized product (Scheme 50). Authors assumed that presence of free alcohol might interfere with the intermediate ruthenium carbene, hindering formation of the desired RCM product **244**. Therefore, alcohol **243** was esterified to give methacrylate **233**, which, however, also did not provide the product **246** upon submission to various RCM conditions [19]. RCM was not proceeded with the silyated compound **245** as well, suggesting the presence of free alcohol might not be responsible for this failure.

**Scheme 51:**
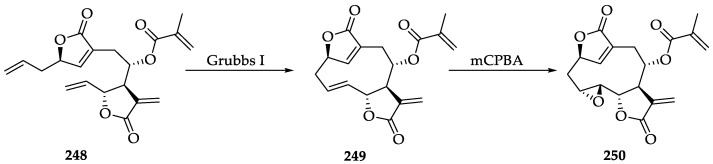
Synthesis of nordeoxyelephantophin (**249**) and norelephantophin (**250**) by Lagoutte et al. [19].

Formation of a medium-sized tri-substituted olefin under RCM conditions is known to be particularly difficult [139,140]. Authors attempted to investigate whether the failure resulted from the presence of the methyl group or the ring tension. For this purpose, RCM precursor **248**, which lacks a methyl group, was used in the reaction (Scheme 51). Upon treatment with Grubbs I in refluxing dichloromethane, **248** was successfully converted to the ring closed product as a single diastereomer, nordeoxyelephantophin **249**. Lastly, **249** was treated with *meta*-chloroperbenzoic acid (*m*CPBA) to generate norelephantopin **250**. Further investigation to cyclize the carbon skeleton of deoxyelephantopin is well elaborated in the reference [19].

**Scheme 52:**
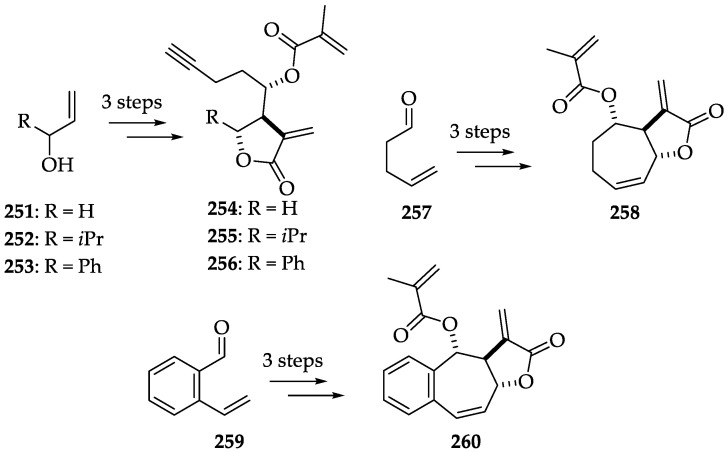
Synthesis of DET-related probes (**254**–**256**) and analogs (**258**, **260**) [134].

In addition, Lagoutte et al. synthesized deoxyelephantopin-related probes **254**–**256** and simplified analogs **258** and **260**, which were utilized to indirectly demonstrate interaction between deoxyelephantopin and PPAR*γ* [134]. Although synthetic access to deoxyelephantopin (**185**) could not be elucidated despite their efforts, preparation of deoxyelephantopin-related analogs and probes, by means of a short and divergent synthetic design, was comprehensively described during the journey towards deoxylelephantopin (Scheme 52).

**Scheme 53:**
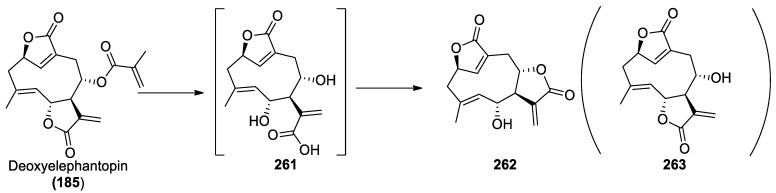
Synthesis of DET-derivatives by Nakagawa-Goto et al. [137].

Nakagawa-Goto et al. reported a number of semi-synthetic derivatives of the natural product and their biological assessment [137]. The synthesis commenced with hydrolysis of deoxyelephantopin (**185**) with NaOH followed by acidic treatment (Scheme 53). According to a previous report on cnicin [141], the group expected C-8 alcohol **263** to be generated. However, C-7-C-8 lactonization of carboxylic acid **261** solely provided less sterically hindered C-6 alcohol **262**.

**Scheme 54:**
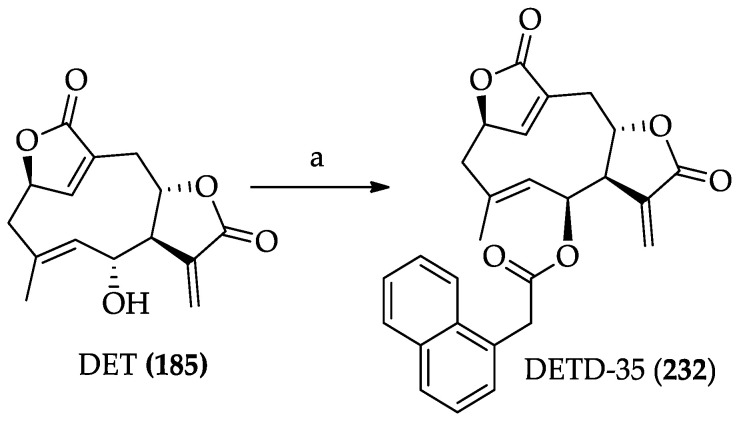
Synthesis of DETD-35 (**232**). Reagents and conditions: (a) RCOOH, EDCI, DMAP, CH2Cl2, rt [137].

Amongst synthesized fifty-eight ester analogs of DET (**185**), an analog with naphthalene acetate, DETD-35 (**232**), was identified as the most potent compound possessing anti-TNBC effects in vitro and in lung metastasis xenograft mouse model (Scheme 54). Moreover, DETD-35 inhibited cell migration, invasion, and motility of MDA-231 cells in a concentration-dependent manner, suggesting that DETD-35 (**232**) might be a potential candidate as a complementary or sensitizing agent to be used in TNBC chemotherapy [137].

#### 2.2.7. Biological Activities of Costunolide (**186**)

The sesquiterpene lactone, costunolide (**186**), which belongs to a member of the germacranolide subclass, was reported to be first isolated from roots of *Saussrea lappa* Clarke, also known as costus [142]. Costunolide (**186**) can be found in extracts of a number of medicinal plants including *Vladimiria souliei* Ling, *Alucklandia lappa* Decne, and *Laurus nobilis* L. as well [143]. Furthermore, costunolide (**186**) is served as a common biosynthetic precursor to three main types of sesquiterpene lactones such as germacranolide, eudesmanolide, and guianolide [144,145].

Costunolide (**186**), like many other naturally occurring lactones, exhibits a variety of biological effects, including anti-cancer, anti-inflammation, anti-oxidant, anti-ulcer, and so on [146,147,148,149]. Anti-cancer effects of costunolide (**186**) have been extensively explored in recent researches [150,151,152]. Costunolide (**186**) is reported to modulate cyclin-dependent kinases (CDKs) and block G2/M phase of the cell cycle to exert anti-proliferative effects on various tumor cells [153,154]. To date, researches indicate that costunolide (**186**) induces apoptosis in three different apoptotic pathways, which eventually influences one another: (a) Mitochondria-mediated (intrinsic) pathway [153,154,155], (b) Death receptor-mediated pathway [156], and (c) Endoplasmic reticulum stress pathway [157,158].

According to studies conducted by Jeong et al., costunolide (**186**) reduced vascular endothelial growth factor (VEGF)-induced proliferation [159]. VEGF is an angiogenic molecule involved in formation of new blood vessels, which is particularly important for survival of cancer cells. Costunolide (**186**) showed inhibitory effects on VEGF-stimulated neovascularization in mouse corneal mouse pocket analysis [159]. Moreover, it was reported in another study that costunolide (**186**) was able to reduce VEGF receptor1 (VEGFR1) and VEGFR2 expression at both mRNA and protein levels, suggesting the potential anti-angiogenic activities of costunolide (**186**) [160].

Migration of cancer cells into surrounding tissues and distant sites in the body to develop tumors in new locations is called metastasis, which is the leading reason for the mortality of many patients with cancer [161]. Lymphatic metastasis, of other various forms of cancer metastasis, is known to be an important determining factor in cancer staging and therapy. Jeong et al. suggested, in part of their investigation, that costunolide (**186**) might provide clinical benefits as an anti-lymphoproliferative agent during tumor metastasis, based on the observed inhibitory activities of the compound on proliferation and capillary formation of temperature-sensitive mouse lymphoid endothelial (TL-LE) cells [162].

**Figure 13:**
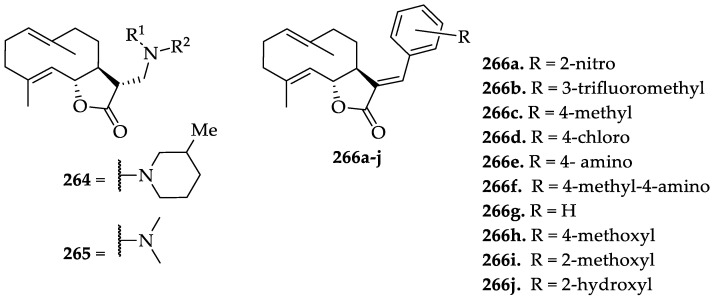
Structures of Costunolide Derivatives.

In an effort to improve cytotoxic effects of costunolide (**186**) on cancer cells, Srivastava, et al. synthesized a number of 13-amino costunolide derivatives by means of Michael-type addition between costunolide (**186**) and various amines [163]. Among the derivatives, 3-methyl piperidine derivative (**264**) possessed 2-fold better cytotoxicity (GI_50_ = 3.3 μM) than that of costunolide (**186**) (GI_50_ = 7.8 μM) on SW-620 colon cancer cells (Figure 13). Two derivatives of hydroxyl piperidine series (structures not shown) also showed also increased cytotoxicity against MIAPaCa2, K-562, and PA-1 cell lines. Moreover, it is noteworthy that the replacement at position-13 with an acyclic *N*,*N*-dimethyl group (**265**) increased cytotoxicity in all cancer cell lines except for A-549 and SW-620. Authors suggested that the amino substituents at position-13 of cosutnolide (**186**) might play an important role in eliciting cytotoxicity [163].

More recent study on the structure and activity relationship of costunolide (**186**) was conducted by Vadaparthi, et al. [164]. The group was able to synthesize a series of analogs by utilizing Heck reaction conditions with various aryl iodides and cytotoxic activities of the compounds were examined against a panel of human cancer cell lines, including cervical cancer (HeLa), breast cancer (MCF-7), lung cancer (A-549), melanoma (B-16), and prostate cancer (DU-145) cell lines in vitro. Amongst derivatives, compound **266c** and **266j** displayed potent activity against Hela, DU-145, and MCF-7 cell lines [164]. Further investigation to establish concrete effects brought by incorporating aromatic and hetero-aromatic rings on costunolide (**186**) scaffold would be indispensable.

Taken all together, costunolide (**186**) is a potential anti-cancer agent with cytotoxic, anti-angiogenic, and anti-lymphoproliferative activities. Therefore, structural modifications of costunolide (**186**) might provide more opportunities in developing new natural lactone-based cancer therapeutics.

#### 2.2.8. Chemistry of Costunolide (**186**)

**Scheme 55:**
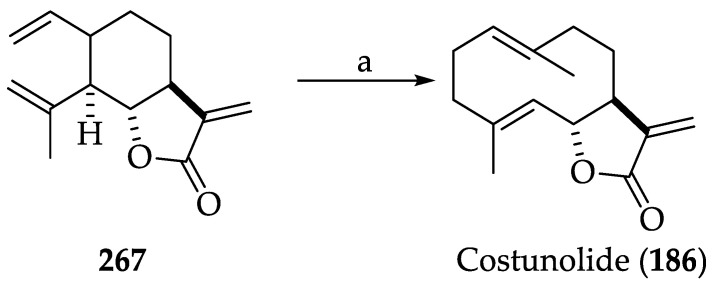
Synthesis of costunolide (**186**) from dehydrosaussurea (**267**). Reagents and conditions: (a) 210 °C [165].

The first total synthesis of (+)-costunolide (**186**) via the Cope rearrangement of synthetic dehydrosaussurea lactone (**267**) was reported by Grieco et al. in 1976 (Scheme 55) [165].

**Scheme 56:**
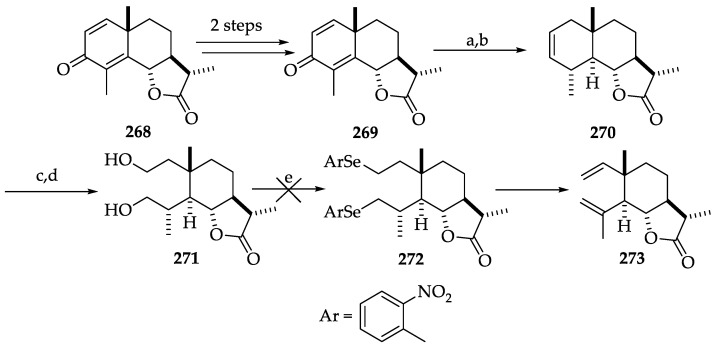
Initial synthetic route for (+)-costunolide (**186**) According to Grieco and Nishizawa. Reagents and conditions: a, TsNHNH_2_, PhH, BF_3_·Et_2_O; b, LDA, THF, −78 → 0 °C, 65%; c, O_3_, CH_2_Cl_2_-MeOH (1:1), −78 °C; d, NaBH_3_, −78 → 25 °C, 91%; e, NO_2_C_6_H_4_SeCN, PBu_3_, THF [165].

The synthesis began with the keto-lactone **269**, which was obtained from santonin **268** in two steps (Scheme 56). Keto-lactone **269** was treated with tosylhydrazine to provide the corresponding hydrazone, which was subsequently treated with lithium diisopropylamide to afford olefin **270**. The resulting olefin underwent ozonolysis followed by treatment with sodium borohydride to yield diol **271**. Authors expected *bis*-*o*-nitrophenyl selenide **272** would be directly obtained from the diol **271**, which would, after oxidation, result in the formation of saussurea lactone **273**. However, this reaction with **271** only provided a side product illustrated in ref. [165].

**Scheme 57:**
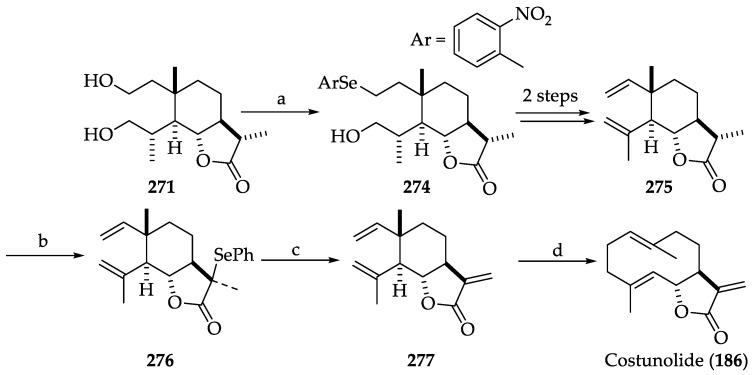
Revised synthetic route for (+)-costunolide according to Grieco and Nishizawa. Reagents and Conditions: a, NO_2_C_6_H_4_SeCN, Bu_3_P, THF-Py (1:1); b, LDA, (PhSe)_2_, HMPA, THF, −78 °C to 20 °C; c, 30% H_2_O_2_ in THF; d, 210 °C [165].

Gratifyingly, diol **271**, upon treatment with *o*-nitrophenyl selenocyanate, tributylphosphine in tetrahydrofuran-pyridine (1:1), provided mono-selenide **274** exclusively (Scheme 57). However, no bis-selenide could be isolated, in spite of their attempt to convert **274** to bis-selenide **272** by previously described reaction condition. In two subsequent reactions, saussurea lactone **275** was afforded from **274**. Selenylation of the lactone **275** followed by treatment with 30% hydrogen peroxide in tetrahydrofuran provided key intermediate dehydrosaussurea lactone **277**, which, upon thermolysis, provided a 20% yield of costunolide (**186**) [165].

**Scheme 58:**
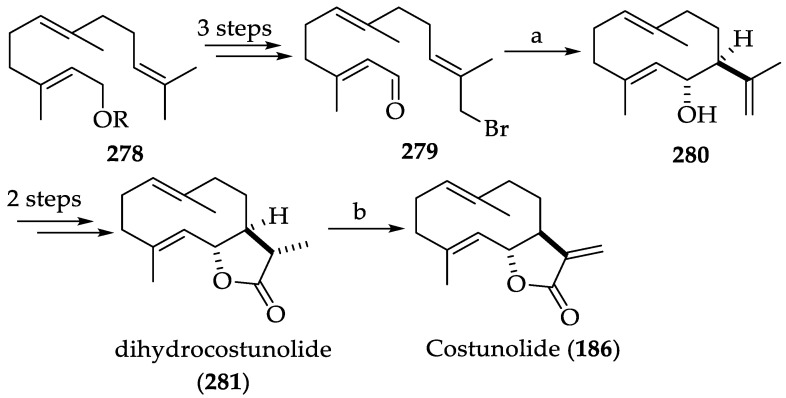
Synthesis of costunolide (**186**) according to Kitagawa et al. Reagents and conditions: (a). CrCl_3_-LiAlH_4_ (2:1), DMF, 42%; (b). (i). TBDMSCl, (ii). 9-BBN, (iii). H_2_O_2_/^−^OH, 72%; (b). (i). LDA, (ii). (PhSe)_2_, (iii). H_2_O_2_, 49% [166].

The second synthesis of costunolide (**186**) was reported by Kitagawa et al. [166]. The synthesis also commenced with a readily available terpenoid, *E*,*E*-farnesol (**278**), which was converted to *ω*-bromo-farnesal (**279**) in three steps (Scheme 58). The key reaction step involved in the synthesis is the intramolecular C-C bond formation of *ω*-bromo-farnesal (**279**) to generate a racemic germacrane-skeletone (**280**), which was mediated by a low valent chromium reagent. Dihydrocostunolide (**281**) obtained in 2 steps from germacrane-skeletone (**280**), was then treated with lithium diisopropylamide (LDA), diphenyl selenide, and hydrogen peroxide to afford costunolide (**186**) as a racemic mixture.

**Scheme 59:**
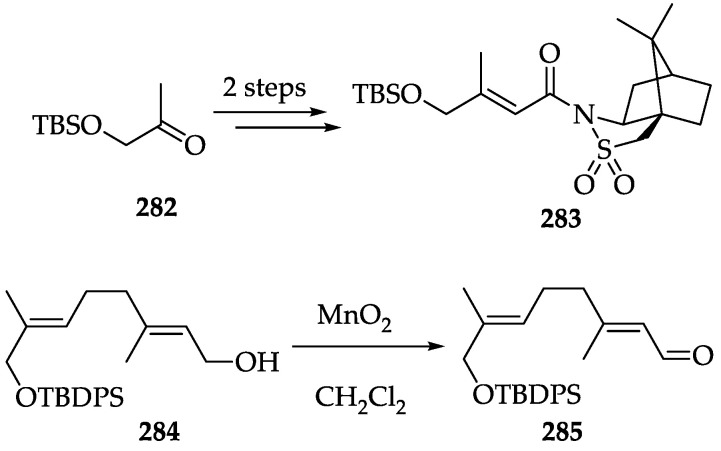
Synthesis of fragments **283** and **285** by Yang et al. [97].

More recently, the highly stereo-controlled total synthesis of costunolide (**186**) by utilizing a synthetic germacrane skeletone has been reported [97]. Yang et al., inspired by the biosynthetic route of sesquiterpene lactones, utilized the 6,7-*trans*-germacrane ring system (**207**) as the key intermediate [167]. An intramolecular *α*-alkylation was employed as the crucial step involved in the synthesis of **207**. The synthesis began with known compound **282**, which underwent a Horner–Wadsworth–Emmons (HWE) reaction with diethylphophonoacetic acid to generate the sulfonate **283** in 58% yield over 2 steps (Scheme 59). Subsequently, another fragment, aldehyde **285**, was prepared by oxidation of the alcohol **284** in 88% yield.

**Scheme 60:**
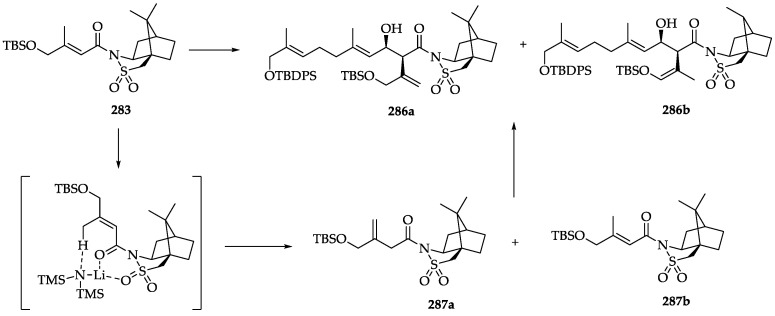
Synthesis of intermediate **286** [97].

Since the aldol reaction between **283** and **285** unfortunately resulted in poor regioselectivity, with expected **286a** obtained as a minor product, an alternative approach was necessitated (Scheme 60). The approach selected by the authors presumably proceeded through a chelated transition state to generate **286a** as the major product over 2 steps.

**Scheme 61:**
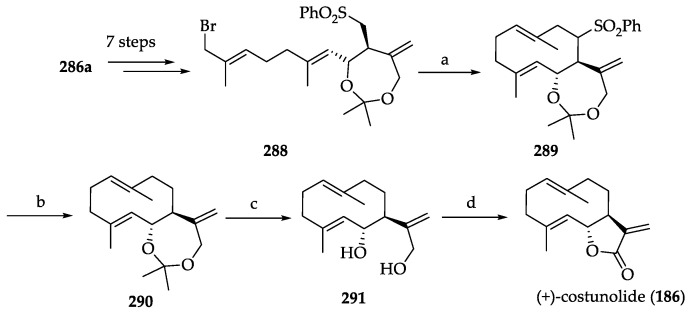
Synthesis of (+)-costunolide (**186**) from the key intermediate **291**. Reagents and conditions: (a) KHMDS, THF, 0 °C, 25 h, 85%; (b) Mg, MeOH, rt, 16 h, 74%; (c) PPTS, MeOH, rt, 20 min, 78%; (d) MnO_2_, CH_2_Cl_2_, rt, 48 h, 82% [97].

Next, the cyclization precursor **288** was obtained over 7 steps. In order to achieve the intramolecular cyclization of **288**, various bases were explored: NaHMDS, LiHMDS, and KHMDS, among which 4 equivalent of KHMDS provided the cyclized product **289** with the best yield (84%) (Scheme 61) [97]. The sulfone group of **289** was removed with treatment of Mg/MeOH to afford **290** in 73% yield, which was then treated with pyridinium *p*-toleunesulfonate (PPTS) in MeOH to give the key intermediate **291** in 78% yield. Lastly, oxidation of **291** with MnO_2_ in CH_2_Cl_2_ produced (+)-costunolide (**186**) in 82% yield.

**Scheme 62:**
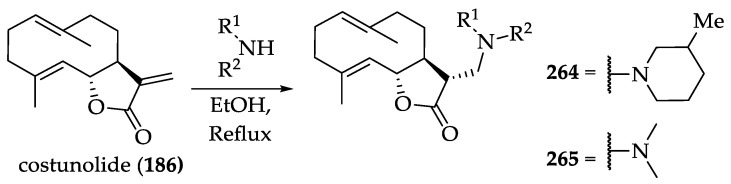
Synthesis of 13-amino costunolide derivatives **264** and **265** [163].

Srivastava et al. synthesized and evaluated 13-amino costunolide derivatives. Simple Michael-type reaction with secondary amines were used to synthesize the derivatives (Scheme 62). The stereochemistry at position-11 has been assumed, based on the following literature finding in the reference [168]. With **264** and **265** as the most potent compounds, authors indicated that *N*,*N*-dimethyl substituation at position-13 of costunolide (**186**) plays a significant role in improving the cytotoxicity of the parent compound (**186**) [163].

**Scheme 63:**
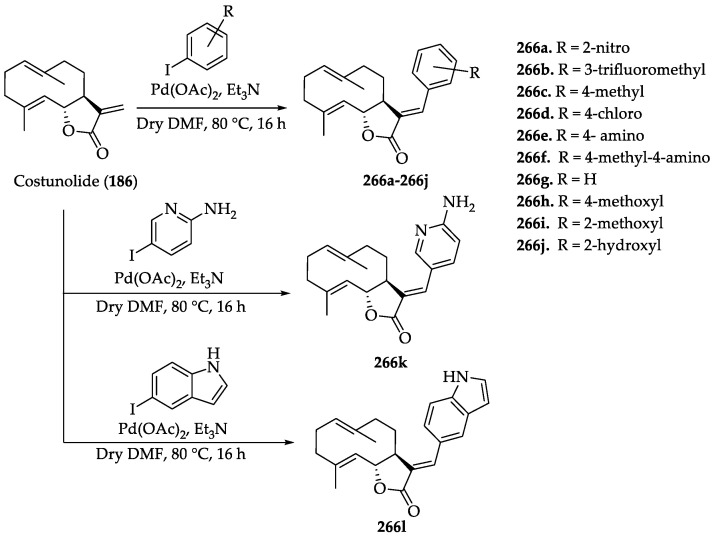
Synthesis of arylated costunolide derivatives (**266a**–**266l**) [164].

Another synthesis of costunolide (**186**) derivatives was reported by Vadaparthi et al. [164]. The α-methylene-γ-lactone moiety was arylated by means of the Pd (II)-catalyzed Heck coupling reaction. In this study, costunolide (**186**) was coupled with various aryl iodides under standard Heck reaction condition (Scheme 63). The reaction seamlessly provided the *E*-olefin products (**266a**–**266l**), exclusively. The C11-C13 olefin was determined to be the *E*-configuration based on the observed diagnostic vinyl proton at C-13, with a range of 7.57–7.97 ppm [164]. Moreover, no structural reorganization or decomposition occurred during the reaction process [164]. Among the derivatives, **266c**, **266d**, and **266j** were highly active against Hela (cervical cancer), DU-145 (prostate cancer), and MCF-7 (breast cancer) cell lines.

Overall, those fruitful researches carried out on the chemistry of costunolide (**186**) provided new insight with regard to the efficient synthesis and structural modification of the natural lactone (**186**).

#### 2.2.9. Biological Activities of Antrocin (**187**)

Antrocin (**187**) is a sesquiterpene lactone mainly present in the extracts of *Antrodia camhorata* (also known as *Antrodia cinnamomea*), a medical mushroom widely used as a dietary supplement for cancer prevention and hepatoprevention in Asia [169]. *Antrodia camhorata* has been traditionally used as a remedy for drug intoxication, abdominal pain, food poisoning, and cancer [170]. Currently, antrocin (**187**) is identified as the compound that contributes to the pharmacological efficacy of *Antrodia camhorata* [169]. Rao et al. explored antrocin (**187**) and its biological mode of action [171]. During the evaluation of antrocin (**187**) on cell proliferation, antrocin exhibited the most potent inhibitory activity against breast cancer line MDA-MB-231 cells with a GI_50_ value of 0.6 μM [171]. Moreover, authors insisted antropin (**187**) might be a novel molecule with efficacy as dual Akt/mTOR inhibitor, based on their observation that the natural lactone inhibits cancer cell proliferation by promoting apoptosis through down-regulation of AKt/mTOR/GSK-3*β*/NF-κB signaling pathways [171].

The group subsequently sought to investigate the capability of antrocin (**187**) to inactivate other survival signaling pathways [172]. In this study, the effect of antrocin (**187**) on a panel of non-small cell lung cancer (NSCLC) cells was examined. H1975 and H441 being two representative cell lines exhibited high sensitivity to antrocin (**187**) (36–78% inhibition, 49–85% inhibition, respectively) [172]. Antrocin (**187**) also inactivated STAT3 and prevented its nuclear localization in H441 cells, indicating its action as a JAK/STAT3 signaling inhibitor. Furthermore, antrocin (**187**) suppressed tumorigenesis in lung cancer mouse xenograft in vivo and enhanced tumor inhibitory response upon combinatorial treatment of antrocin (**187**) and JAK2 inhibitor [172].

More recently, Chen et al. reported antrocin (**187**) as an anti-TNBC agent without apparent systematic toxicity, based on their preliminary toxicological evaluation performed with antrocin (**187**) in a 28-day rat study (at 37.5 mg/kg) [173]. In addition, another study demonstrated that antrocin (**187**) effectively enhances sensitization radio-resistant prostate cancer cells to radiation [174]. Given together, antrocin (**187**) has the capability to be a therapeutic and sensitizing agent for various cancers.

#### 2.2.10. Chemistry of Antrocin (**187**)

**Scheme 64:**
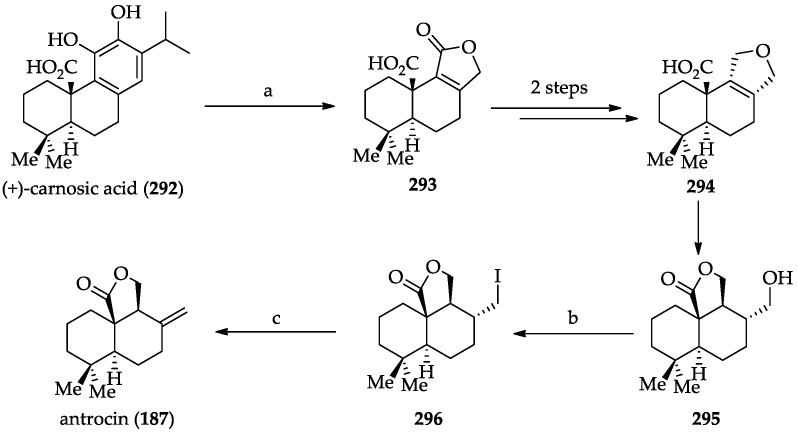
Synthesis of antrocin (**187**) from (+)-carnosic acid (**292**). Reagents and conditions: (a) O_3_, CH_2_Cl_2_/MeOH (3/1), 78 °C, 1.5 h, then NaBH_4_ (6.0 equiv.), 78 °C to rt, 1 h (58%); (b) Ph_3_P (1.3 equiv), I_2_ (1.5 equiv), imidazole (1.5 equiv), THF, 0 °C to rt, 1 h (99%); and (c) DBU (10.0 equiv), toluene, 80 °C, overnight (50%) [175].

A concise and asymmetric synthesis of antrocin (**187**) has been reported by Li et al. [175]. The synthesis began with the naturally abundant carnosic acid as a chiral pool. After subjection of carnosic acid (**292**) to ozonolysis, followed by reduction with NaBH_4_, optically pure lactone **293** was afforded in 58% yield (Scheme 64). Lactone **293** was directly converted to hydroxyl lactone **295** in two steps, which was subsequently treated with I_2_ in the presence of PPh3 and imidazole to produce iodide **296**. Lastly, treatment of **296** with DBU afforded antrocin (**187**) in 50%. The asymmetric synthesis of antrocin (**187**) as achieved by the group with a 16.1% overall yield in five steps [175]

Recently, the total synthesis of natural (−)-antrocin (**187**) and its enantiomer has been reported [176]. Despite remarkable antitumor activities, the difficulties to prepare (−)-antrocin (**187**) triggered researchers to find a synthetic alternative. Since there was no available simple and inexpensive synthetic method for (−)-antrocin (**187**) until recently, authors focused on developing a synthetic approach using inexpensive, readily available starting material 6-methoxy-2-tetralone **297** and simple chemical operations.

**Scheme 65:**
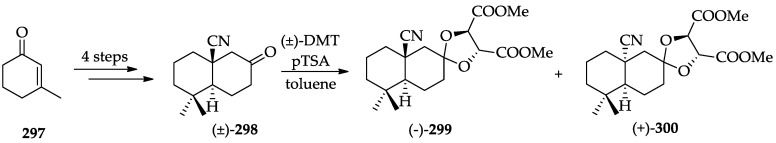
Synthesis of intermediate (−)-**299** [176].

From commercially available starting material **297**, *trans*-cyano bicyclic ketone (±)-**298** was obtained as a major product over 4 steps (Scheme 65). Resolution of the compound (±)-**298** was performed by utilizing (+)-dimethyl tartrate (DMT), which yielded separable 1:1 diasteromic mixture of ketal (−)-**299** and (+)-**300**.

**Scheme 66:**
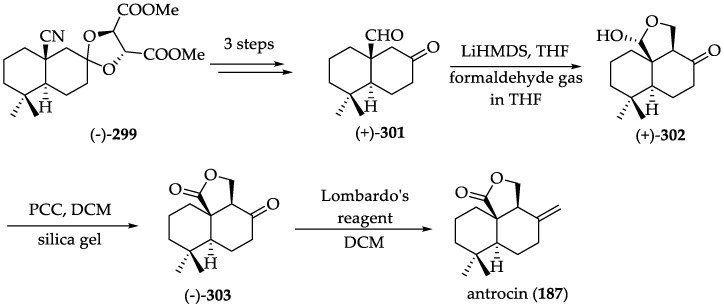
Complete Synthesis of antrocin (**187**) [176].

Ketal (−)-**299** was selected for further reaction to complete the synthesis of antrocin (**187**). Keto aldehyde (+)-**301** was prepared from (−)-**299** over 3 steps (Scheme 66). Next, *α*-hydroxymethylation of (+)-**301** by reacting with lithium enolate with gaseous formaldehyde in THF yielded a single lactol (+)-**302**. The authors proposed that this reaction is controlled kinetically [176]. Lactol (**302**) was then oxidized with silica-supported pyridinium chlorochromate (PCC) to give lactone (−)-**303**. Authors attempted both standard and modified Wittig olefination (Ph_3_PCH_2_ and THF, 0 °C and Ph_3_PCH_2_, toluene, and *t*-BuOH, rt) [177], which ended up with a poor conversion rate (30–30%). Gratifyingly, another attempt with non-basic Lombardo’s reagent (Zn, TiCl_4_, CH_2_Br_2_) led to successful olefination of ketone (−)-**303** to antrocin (**187**) in excellent yield (98%) [178].

#### 2.2.11. Biological Activities of EM23 (**188**) and Brevilin A (**189**)

EM23 (**188**) is a natural sesquiterpene lactone derived from *Elephantopus mollis*. Shao et al. investigated anticancer effects of EM23 (**188**). EM23 (**188**) exhibited growth inhibitory activity against various cancer cell lines, including A549 (lung cancer), MCF-7 (breast cancer), TE-1, EC109, and EC9706 (esophageal cancer), CaSki and SiHa (cervical cancer), and HL-60 and K562 (leukemia) [179]. EM23 (**188**) showed the most potent anti-proliferative activity against CaSki and SiHa with a GI_50_ value of 5.8 and 6.6 μM, respectively. Furthermore, the authors proposed the mechanism of EM23-induced apoptosis. Thioredoxin reductase (TrxR) catalyzes the reduction of thioredoxin (Trx), which participates in various cellular processes [180]. Overexpression of Trx/TrxR is found to be related in the development and progression of cancer [181,182]. EM23 (**188**) inhibits the expression levels of TrX/TrxR to facilitate ROS accumulation, which results in the dissociation of ASK1 from complex with Trx and activation of downstream JNK signaling pathway [179]. Taken together, EM23 (**188**) can be potentially applied as an anticancer agent for human cervical cancer by a structural modification to achieve further enhancement in potency.

**Figure 14:**
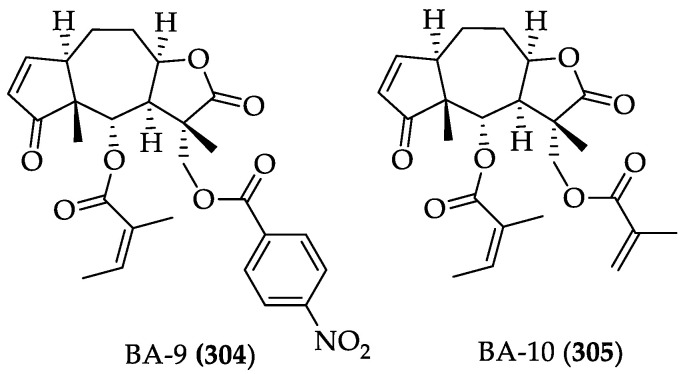
Structure of Brevilin A (**189)** derivatives BA-9 (**304**) and BA-10 (**305**).

Brelivin A (**189**) is a bioactive component mainly present in *Centipeda minima* (L.) A [183]. It has been reported to display anticancer activities. In acute promyelocytic leukemia HL-60 cells, Brevilin A (**189**) induced apoptosis through a mitochondrial/caspase pathway [184]. Moreover, treatment of Brevilin A (**189**) reversed vincristine resistance in multidrug-resistant colorectal cancer cell line HCT-8/VCR by inhibiting the intracellular accumulation of YB-1 and down-regulating MDR1 expression, two important factors closely related to multidrug resistance. This suggests Brevilin A (**189**) as a potential anticancer drug adjuvant to reverse drug resistance in chemotherapy [185]. In addition, Chen et al. reported that Brevilin A (**189**) acts as a STAT3 signaling inhibitor [186]. Persistent STAT3 activity is associated with cancer progression, most of which show aberration of JAKs, Src, or other receptor tyrosine kinases. Brevilin A (**189**) inhibited both constitutively activated-STAT3 driven DU145 (prostate cancer) and MDA-MB-468 (breast cancer) cell lines [186]. It is also noteworthy that Brevilin A (**189**) specifically inhibits JAKs without other signaling proteins such as p65, AKT, GSK-3β, and Src. A number of Brevilin A (**189**) derivatives were synthesized and their anticancer potential was evaluated in a structure-activity relationships study conducted by Lee et al. [187]. During the course of the study, it was found out that the alkene or carbonyl of the enone moiety is crucial in achieving cytotoxicity. Most notably, introducing different substituents to the *α*-position of the *γ*-lactone ring exhibited a significantly increased cytotoxicity in the tested cancer cell lines. In the MDA-MB-231 and A549 cell lines, BA-9 (**304**) and BA-10 (**305**) displayed a GI_50_ value of 4.647 μM and 6.385 μM against MDA-MB-231 and 6.239 μM and 6.392 μM against A549, respectively (Figure 14). These values exhibited by the derivatives are roughly two-fold greater than that of the parent compound Brevilin A (**189**) (GI_50_ in MDA-MB-231 = 7.03 μM, A549 = 10.09 μM), indicating potential of BA-9 (**304**) and BA-10 (**305**) as promising candidates for further development as cancer therapeutics [187].

#### 2.2.12. Chemistry of EM23 (**188**) and Brevilin A (**189**)

**Scheme 67:**
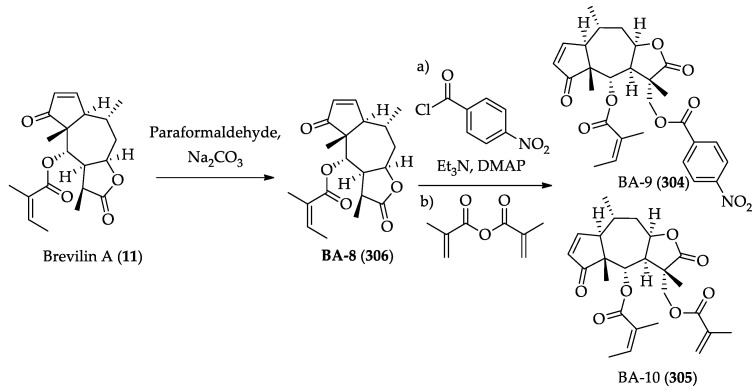
Synthesis of Brevilin A (**11**) derivatives **304** and **305** [187].

To the best of our knowledge, there are no reports elucidating total synthesis of EM23 (**188**) and Brevilin A (**189**) up-to-date. Semi-synthetic derivatives of Brevilin A (**189**) were prepared by Lee et al. [187]. Key intermediate BA-8 (**306**) was obtained via aldol reaction between Brevilin A (**189**) and paraformaldehyde in the presence of sodium carbonate (Scheme 67). Subsequently, C11-hydroxylmethyl of BA-8 (**306**) was actylated with *p*-nitrobezoyl chloride and methacrylic anhydride to yield BA-9 (**304**) and BA-10 (**305**), respectively.

### 2.3. Diacylglycerol Lactones

Diacylglycerol lactones (DAGLs) are synthetic lactones derived from *syn*-1,2-diacylglycerol (DAG), which is a key lipid second messenger that binds to the C1 domain in many regulatory proteins [188]. This lipophilic second messenger plays a key role in signal transduction pathways [14]. DAG especially acts as an endogenous activator by binding to the C1 domain of protein kinase C (PKC). Upon binding, it allosterically activates the enzyme in the presence of phospholipid [189]. The PKC family can be classified into three isoenzyme groups: conventional (*α*, *β*I, *β*II, and *γ*), novel (*δ*, *ε*, *η*, and *θ*), and atypical (*ζ* and *ι*/*λ*) [189]. Only conventional and novel isoenzymes contain C1 regions. PKC isoforms constitute the most prominent family of signaling proteins that control cellular functions such as proliferation, survival, motility, tumorigenesis, and metastasis [189]. Therefore, their significance in pathogenesis has driven much interest in the C1 domain as a therapeutic target [189]. Furthermore, implication of PKCs in a range of cancer in different organs increases its potential as a therapeutic target [190,191,192], which eventually led to a thorough investigation on DAG-based small molecule PKC modulators.

#### 2.3.1. Biological Activities of Diacylglycerol Lactones

**Figure 15:**
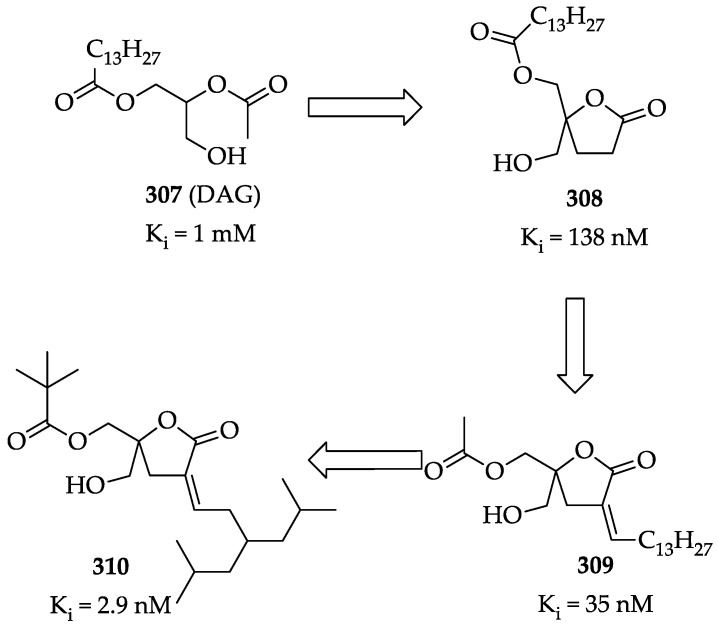
DAG-Lactones as PKC ligands [189].

DAG binds to the C1 domain of PKC isoenzymes containing a cysteine-rich, zinc finger-like motif [193]. However, competence between DAG and phorbol esters might occur because they share the same binding site. The binding affinity of phorbol esters is higher than that of DAG by at least 3 orders of magnitude [14]. Upon binding of the phorbol esters, PKC activation bypasses the normal physiological signal-mediated mechanism, in which responses irrelevant to DAG could be activated [194]. To overcome this challenge, the group of Blumberg and Marquez designed a series of structurally simple cyclic DAG-based molecules to surpass the binding affinity of DAG to PKC [14]. They reasoned that the low binding affinity of DAG is caused by the flexible nature of the glycerol backbone and, therefore, giving it an entropy penalty by restricting the conformation would increase the binding affinity towards PKC. With this rationale, Kang et al. obtained 4,4-disubstituted-*γ*-butyrolactone as an ideal glycerol template [189]. Their extensive SAR studies based on pharmacophore-guided and receptor guided approaches provided notable findings to increase binding affinity from DAG (**307**, K_i_ ≈ 1 μM) through cyclization to the structurally constrained 5-tetradecanoyl DAG-lactone (**308**, K_i_ ≈ 138 nM), shifting of the hydrophobic alkyl chain from the 5-acyl to the 3-alkylidene position (**309**, K_i_ ≈ 30 nM), and incorporating highly branched alkyl chain (**310**, K_i_ ≈ 2.9 nM) (Figure 15).

**Figure 16:**
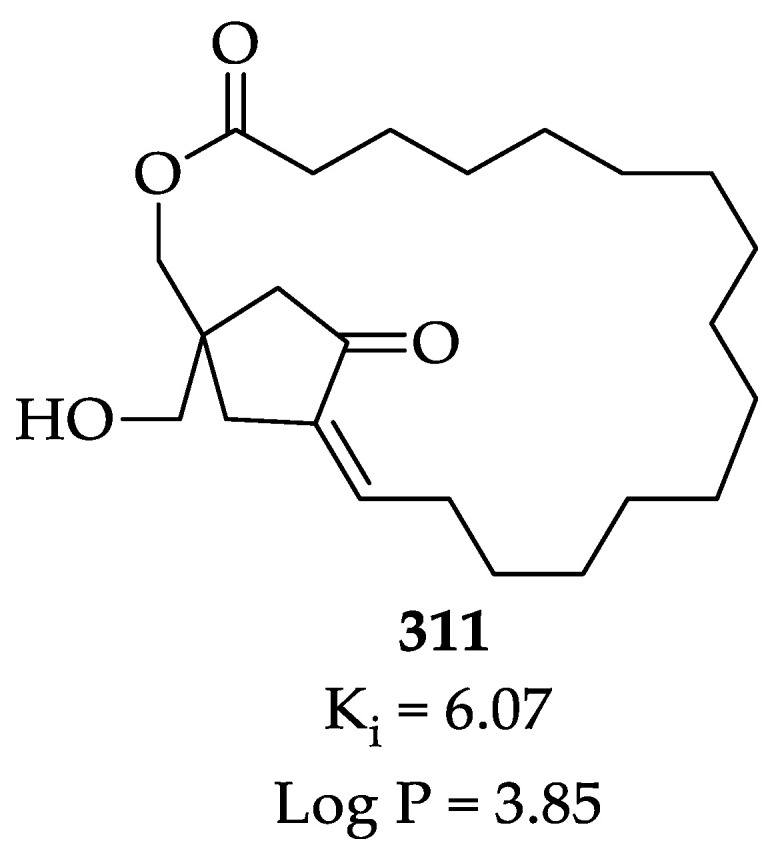
Structure of macrocyclic DAG-bis-lactones **311** [189].

Moreover, the authors envisioned that an additional conformational restriction of the remaining 5-acyl group would achieve further enhancement in binding activity [189]. To this end, macrocyclization strategy to link the two terminal alkyl ends of the 5-acyl and 3-alkylidene groups was used. They expected that this strategy would allow the ester to adopt the preferred *S*-trans configuration for ring sizes greater than ten, as well as rendering the physicochemical properties of the ligand suitable as drug candidates [189]. Among synthesized macrocyclic DAG-bis-lactones evaluated, compound **311** exhibited in K_i_ value of 6.07 nM against PKC_α_ (Figure 16). In addition, the molecular docking study of **311** with PKC_α_ suggests that the macrolactone **311** exclusively binds in the sn-1 binding mode to the C1b domain of the protein [189].

**Figure 17:**
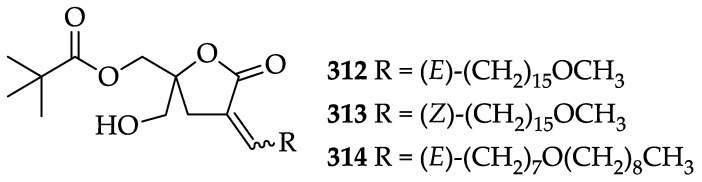
Structure of DAG-lactones with polar 3-alkylidene chain (**312**–**314**) [195].

Kang et al. synthesized and investigated a series of DAG-lactones with polar 3-alkylidene substituents as PKC*α* and antitumor agents (Figure 17). The structure-activity relationships revealed that compounds **312**, **313**, and **314** with an ether side chain have high binding affinities (K_i_ = 3–5 nM) and excellent antitumor effects on colon cancer (Colo205, GI_50_ = 0.120–0.260 μg/mL) and leukemia cancer (K562, GI_50_ = 0.140–0.200 μg/mL) cell lines [195].

**Figure 18:**
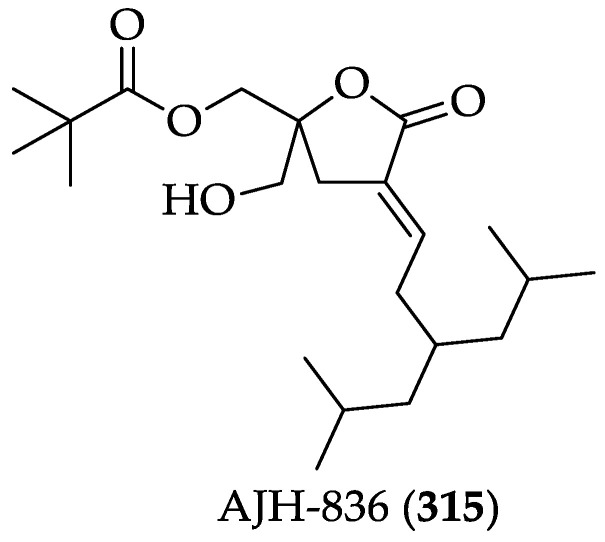
Structure of AJH-836 (**315**).

Individual PKC isotypes show different patterns of tissue distribution, subcellular localization, substrate specificity, and biological functions [196,197]. Consequently, it is an important issue to discriminate PKC isotypes to avoid an undesired effect. Ann et al. focused on protein kinase C epsilon (PKC*ε*), a calcium-independent but phorbol ester/diacylglycerol dependent PKC isotype [198]. PKC*ε*, along with other frequently expressed PKCs (PKC*α* and PKC*δ*), is reported to trigger mitogenic/tumor promoting or conversely anti-mitogenic/tumor suppressor responses [14]. They have identified AJH-836 (**315**) (Figure 18), which has the *sn*-2 carbonyl substituted with a saturated-branched alkyl chain with an *E*-conformation, as a selective ligand for PKC*ε* (K_i_ of PKC*α* = 46 nM, K_i_ of PKC*ε* = 1.43 nM). Moreover, it should be noted that the selectivity was further enhanced in the nuclear membrane conditions, indicating strengthened interactions between the DAG-lactone side chain and the protein-membrane interface [198]. With the help of chemically modified DAG-lactone, AJH-836 (**315**), additional efforts were made to highlight the importance of PKCs in the transcriptional gene regulation in lung cancer cells [199]. Authors have added that the discovery of selective C1 domain ligands may lead to promising therapeutic leads and pharmacological tools for identifying pathophysiological mechanism of diseases [199].

#### 2.3.2. Chemistry of Diacylglycerol Lactones

**Scheme 68:**
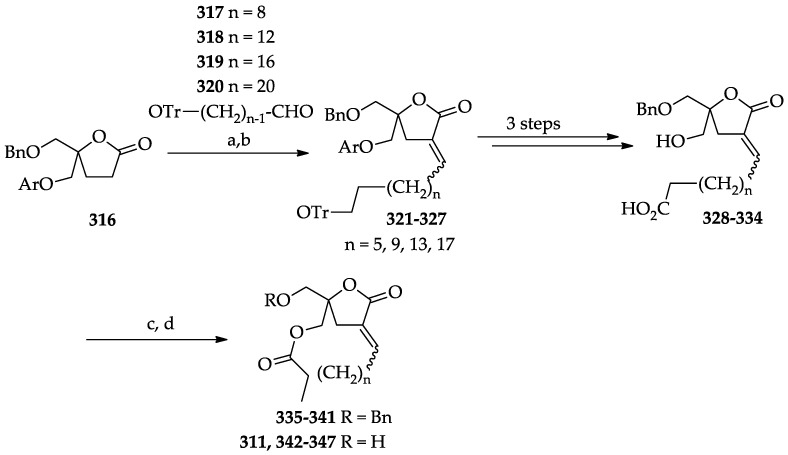
Synthesis of DAG-bis-macrolactones [189]. Reagents and conditions: (a) (i) LiHMDS, THF, −78 °C; (ii) RCHO (13–16); (b) (i) MsCl, NEt_3_, CH_2_Cl_2_; (ii) DBU, 45–52% in 2 steps; (c) DMAP, DMAP/HCl, DCC, CH_2_Cl_2_, 60–70%; (d) BCl_3_, CH_2_Cl_2_, 80–92%.

Synthesis of DAG-bis-macrolactone commenced with condensation reaction of *γ*-lactone with aldehydes **317**–**320** to afford *β*-hydroxylactones, which were converted into 3-alkylidene *γ*-lactone (**321**–**327**) as mixtures of *E*/*Z* isomers (Scheme 68). With the key intermediate **328**–**334** obtained over 3 steps, macrolactonization was utilized according to Keck and Boden’s method to yield 13, 17, 21, and 25-membered macrolactones (**335**–**341**), respectively [200]. Lastly, benzyl group was removed to provide the target macrolactones (**311**, **342**–**347**) [189].

**Scheme 69:**
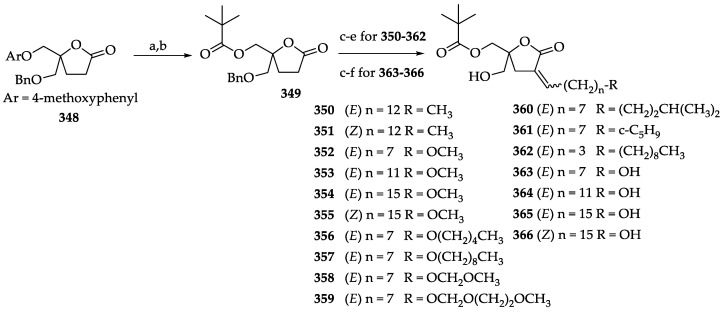
Synthesis of hydroxyl and ether DAG-lactone analogs [195]. Reagents and conditions: (a) CAN, CH_3_CN–H_2_O, 0 °C; (b) (CH_3_)_3_CCOCl, Et_3_N, DMAP, CH_2_Cl_2_; (c) LiHMDS, CH_3_(CH_2_)_12_CHO for **350**–**351**, RO(CH_2_)nCHO for **352**–**362**, TrO(CH_2_)nCHO for **363**–**366**, THF, −78 °C; (d) (i) MsCl, NEt_3_, CH_2_Cl_2_, (ii) DBU; (e) BCl_3_, CH_2_Cl_2_, −78 °C; (f) CF_3_CO_2_H, CH_2_Cl_2_, 0 °C.

Due to high lipophilicity of synthesized DAGLs, it was attempted to reduce the lipophilicity by incorporating more polar side substituents in the 3-alkylidene chain. DAG-lactones with polar 3-alkylidene chains were synthesized by alkylation of the protected 5,5-disubstituted *γ*-lactone with various polar side chains (Scheme 69) [195].

**Scheme 70:**
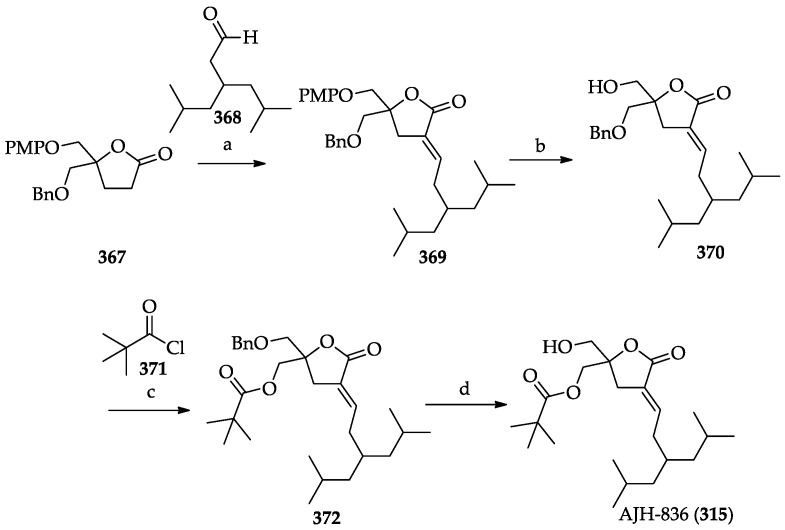
Synthesis of AJH-836 (**315**). Reagents and conditions: (a). (i) LiHMDS, THF, R_2_CHO, −78 °C. (ii) MsCl, CH_2_Cl_2_, DBU; (b) BCl_3_, CH_2_Cl_2_, −78 °C or CAN, CH_3_CN/H_2_O; (c) EDC, DMAP, CH_2_Cl_2_, r.t.; (d) BCl_3_, CH_2_Cl_2_, −78 °C or CAN, CH_3_CN/H_2_O [198].

AJH-836 (**315**), a PKC*ε* selective DAG-lactone, was synthesized by Ann et al. [198]. The synthesis started with *p*-methoxyphenyl (PMP) and benzyl (Bn) protected racemic lactone (Scheme 70). Aldehyde **368** was reacted with the **367** to form olefin **369** via aldol condensation. After removal of PMP protection, the intermediate **370** was reacted with acyl chloride **371**, followed by benzyl deprotection to afford the target molecule **315**.

### 2.4. Diterpene Lactones

Diterpenes are classes of natural products mostly originated from microbes or secondary metabolites of fungal sources. Diterpenes can be further classified into subdivisions according to its structural feature (bicyclic, tricyclic, and tetracyclic diterpenes). This class of compounds attracted researchers due to their potential biological activities including anti-cancer, anti-oxidant, and anti-inflammatory effects [201,202].

#### 2.4.1. Biological Activities of Andrographolide (**373**)

Andrographolide (**373**) is a simple bicyclic labdane diterpene lactone belonging to the isoprenoid family of natural products and is a well-known medicinal plant, which has been widely used in Asia [203]. The lactone is isolated from the stem and leaves of *Andrographis paniculata* (Burm.f.) Nees, also known as King of Bitters. [204]. The characteristic structural features of andrographolide (**373**) include: *α*,*β*-unsaturated *γ*-butyrolactone ring, three hydroxy groups (at C-3, C-14, and C-19), and two olefin bonds (Δ^8(17)^ and Δ^12(13)^). Biological responses elicited by andrographolide (**373**) is mainly due to its ability to form H-bonds with biological substrates by utilizing the hydroxyl group [205]. Andrographolide (**373**) having potent cytotoxic effects against various cancer cells, it has been reported that andrographolide (**373**) exerts the anti-cancer effects by modulating several cancer-related pathways and proteins including JNK-signaling pathway, NF-*κ*B and PI3K signaling pathway, cyclins and cyclin-dependent kinases (CDKs), metalloproteinases (MMPs) and tumor suppressor proteins (p53 and p21) [206].

**Figure 19:**
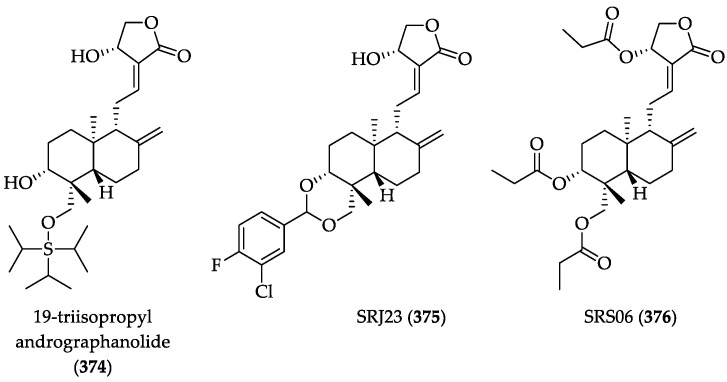
Structure of andrographolide analogs.

Dai et al. demonstrated that andrographolide (**373**) prevents proliferation of human gastric cancer cell line SGC-7901 by blocking cell cycle progression, promoting intrinsic apoptosis, and/or repressing invasive activity [207]. Upon increasing concentrations of andrographolide (**373**) (10, 20, and 40 μg/mL), cell-cycle inhibitory proteins (cyclin B1 and CDC2) and proapoptotic protein (Bax) were upregulated and antiapoptotic protein (Bcl-2) was downregulated [207]. 19-triisopropyl andrographanolide (**374**), an analogue of androgrphanolide (**373**), showed potent cytotoxic activity against gastric cancer cell lines with a GI_50_ value of 6.3 μM and 1.6 μM for MKN-45 and AGS cell lines, respectively (Figure 19). On the other hand, the parent andrographanolide (**373**) is shown to be less potent than the analog (**374**) with the GI_50_ values of >50 μM in MKN-45 and 11.3 μM in AGS cell lines [208].

Andrographanolide (**373**) is also found to suppress tumor proliferation in prostate cancer cells by modulating proimflammatory cytokines (interleukin (IL)-6) and chemokines (CXCl11, CXCR3, and CXCR7) [209]. Moreover, administration of andrographanolide (**373**) to DU145 (prostate cancer cell)-xenografted mice delayed tumor growth without obvious toxicity [210]. SRJ23 (**375**), another andrographanolide (**373**) analog, was synthesized to improve the antitumor activity of the parent compound (**373**) against prostate cancer cell lines (Figure 19). SRJ23 (**375**) selectively inhibited a prostate cancer cell (PCa) with a 50-fold improved GI_50_ (0.4 μM) than that of the parent compound andrographanolide (**373**) (GI_50_ = 19.95 μM) [211].

Colorectal cancer is a frequently diagnosed solid tumor. The main issue in this cancer is the high recurrence rate caused by acquired resistance to chemotherapies such as 5-fluorouracil (5-FU) and cisplatin [212,213]. In a study conducted by Wang et al. [212], andrographanolide (**373**), when treated to 5-FU-resistant colorectal cancer cell line (HCT116/5-FUR), synergistically enhanced 5-FU-induced apoptosis. This suggests that andrographanolide (**373**) could reverse chemotherapy resistance and act as a sensitizing agent in colorectal cancer Moreover, the clinical relevance of andrographanolide (**373**) in combination with capecitabine is being investigated, which initiated a clinical trial in 2014 [214].

Another type of cancer highly related to poor prognosis and high recurrence rate is non-small-cell lung cancer (NSCLC). Andrographanolide (**373**) showed synergistic antitumor activity with chemotherapeutic agents cisplatin and paclitaxel, although the molecular mechanism behind this synergism is still unclear [215,216]. Lim et al. reported an andrographanolide (**373**) analog, 3,14,19-tripropionylandrographolide (SRS06, **376**), which shows a distinct inhibitory activity against A549 NSCLC cell line. SRS06 (**376**) was able to suppress cancer cell proliferation by downregulating the levels of NF-*κ*B protein and inhibiting p65 DNA binding activity at a concentration of 5 μM [217].

Despite various antitumor activities of andrographanolide (**373**), poor solubility and relatively low potency still remained to be the main hurdles for further advancement into clinical development. To overcome this challenge, more efforts in modification and optimization of the chemical structures would be needed.

#### 2.4.2. Chemistry of Andrographolide (**373**)

**Scheme 71:**
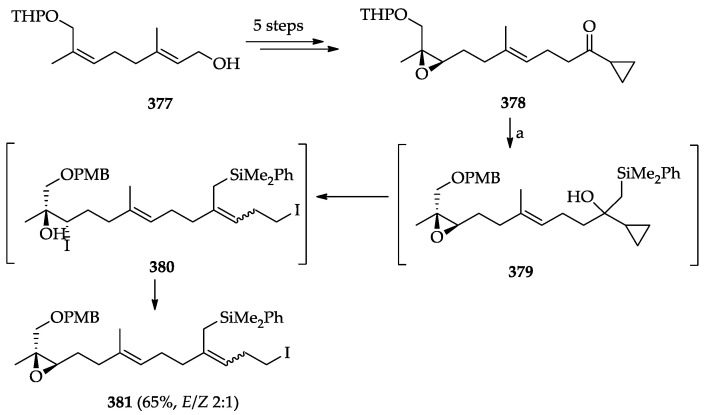
Synthesis of key intermediate **381** [218]. Reagents and conditions: (a). (i) PhMe_2_SiCH_2_MgCl, CeCl_3_, THF, 0 °C–23 °C; (ii) 2.5 equiv MgI_2_·(OEt_2_)n (0.25 M in Et_2_O/PhH (1:1)), PhH, 50 °C, 15 min; (iii) K_2_CO_3_, MeOH, 23 °C, 65%.

Gao et al. reported the first total synthesis of (−)-andrographolide (**373**) via biomimetic cyclization approach, in which an epoxy homoiodo allylsilane precursor (**381**) was utilized [218]. The synthesis commenced with geraniol **377**, which was readily converted to epoxide **378** in 79% yield over 5 steps (Scheme 71). For the efficient preparation of the key precursor **381**, authors optimized the protocol based on their previous protocol, which includes 3 reaction steps [219]. Cyclopropyl ketone (**378**) underwent chemoselective 1,2-addition with (phenyldimethylsilyl)methylcerium chloride to afford cyclopropyl carbinol (**379**), which, upon exposed to MgI_2_, provided intermediate **380** as a crude mixture. Next, the resulting crude intermediate **380** was briefly treated with K_2_CO_3_ in methanol to give the key intermediate **381**. Moreover, the authors found out that reaction temperature is critical for Julia-type cyclopropane ring-opening of **379** [218].

**Scheme 72:**
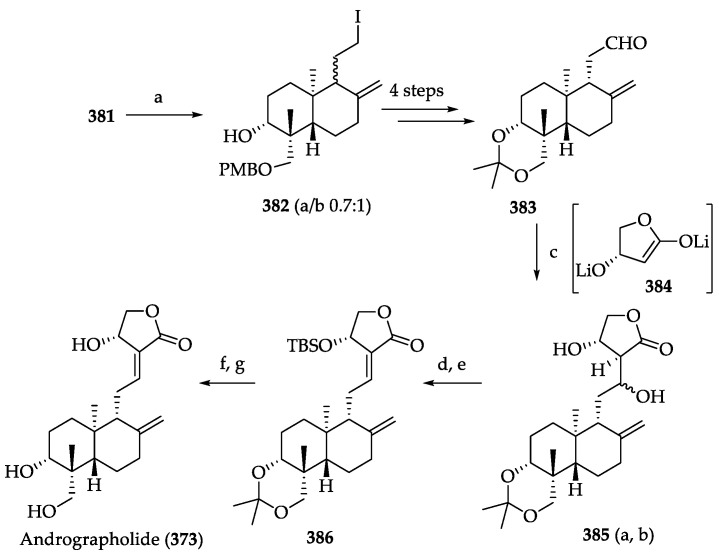
Synthesis of andrographolide (**373**). Reagents and conditions: (a) 2.0 equiv SnCl_4_, CH_2_Cl_2_, −40 °C, ca. 1 min.; (b) 1.6 equiv (*S*)-(−)-*β*-hydroxy-*γ*-butyrolactone, 3.2 equiv LDA, THF/HMPA (4:1), −78 °C–30 °C, 64% (80% brsm); (c) TBSCl, imidazole, DMF, 23 °C, 76%; (d) MsCl, Et_3_N, CH_2_Cl_2_, −78 °C–0 °C, 1 h; then *i*Pr_2_NEt, CH_2_Cl_2_, 23 °C, 55%; (e) TBAF, THF, 23 °C, 57%; (f) HOAc/H_2_O (7:3), 23 °C, 89% [218].

The key reaction step utilized in this study is the biomimetic cation-olefin annulation of epoxy homoiodo allylsilane precursor **381** to form bicyclic iodide **382** (Scheme 72). By using the optimized cyclization condition (SnCl_4_ in CH_2_Cl_2_ at −40 °C), bicyclic iodide **382** was furnished as a mixture of C_9_ epimers (*α*/*β* 0.7:1). Subsequently, bicyclic iodide **382** was converted to bicyclic aldehyde **383** over 4 steps, which underwent aldol condensation with the corresponding lithium enolate of (*S*)-(−)-*β*-hydroxy butyrolactone (**384**) to give dihydroxy lactone **385** as a mixture of C-12 epimers. Compound **385** was then selectively O-silyated via the mesylate intermediate to provide *E*-configurated lactone **386** in a regio- and stereoselective manner [218]. Lastly, desilylation and acetonide cleavage of **386** provided (−)-andrographolide (**373**), which was spectroscopically identical to the natural andrographolide [220].

**Scheme 73:**
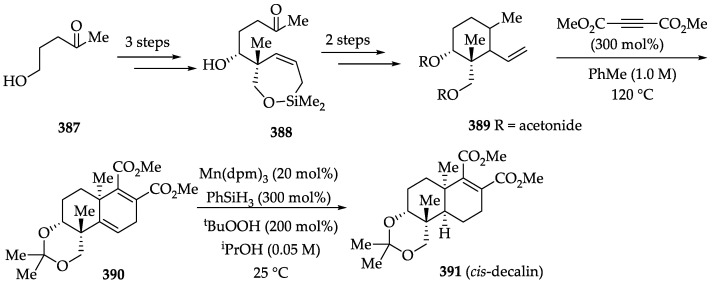
Synthesis of *cis*-Decalin **391** [221].

A more concise and convergent enantioselective total synthesis of andrographolide was recently reported by Yang et al. [221]. Key transformations utilized in the synthesis include: (1) formation of quaternary C4 stereocenter via iridium-catalyzed carbonyl reductive coupling, (2) establishment of the trans-decaline skeleton via diastereoselective alkene reduction, and (3) installation of the *α*-alkylidene-*β*-hydroxy-*γ*-butyrolactone via carbonylative lactonizaition. The synthesis began with preparation of acetonide **389**, which was achieved via a 6-step reaction (Scheme 73). The authors predicted that **389** would undergo cycloaddition diastereoselectively from the convex face of the bicycle. As expected, cycloadduct **390** was obtained as a single stereoisomer from Diels–Alder cycloaddition of **389** with dimethylacetylene dicarboxylate (DMAD) [221]. For the construction of the *trans*-decalin ring, manganese-catalyzed hydrogen atom transfer (HAT) was chosen because it enables diastereselective hydrogenation of the targeted alkene [222]. However, HAT reduction of cycloadduct **390** exclusively provided the *cis*-decalin **391** instead.

**Scheme 74:**
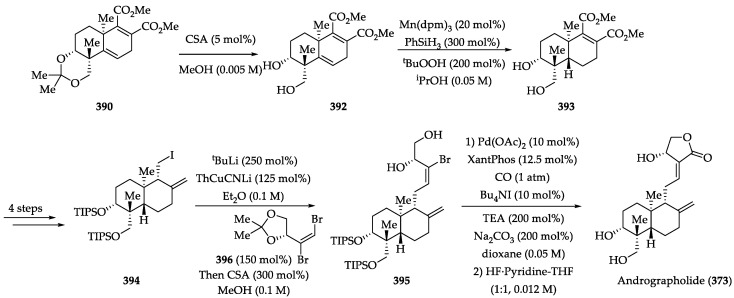
Synthesis of andrographolide (**373**) [221].

This result is presumably due to conformational constraint caused by the acetonide moiety, as diol **392** smoothly underwent HAT reduction to afford *trans*-decalin (**393**) as a single diastereomer [221], which subsequently converted to iodide **394** over 4 steps (Scheme 74). The authors found installation of the *α*-alkylidene-*β*-hydroxy-*γ*-butyrolactone especially challenging because of competing elimination to form diene by-products or halide reduction. Pleasingly, diol **395** was obtained in 54% yield via chemoselective cross-coupling at the terminal vinyl bromide upon treatment with 2-thienyl(cyano)copper lithium followed by exposure to vinyl bromide **396**. Lastly, bromoalcohol **395** underwent carbonylative lactonization to furnish *α*-methylene-*β*-hydroxy-*γ*-butyrolactone, which upon removal of the triisopropylsilyl ethers provided the target compound andrographolide (**373**). Moreover, it is noteworthy that the described route accomplished the synthesis of andrographolide (**373**) in 14 steps, which is 10 steps lesser than the previous report [218,221].

#### 2.4.3. Anti-Cancer Activities of Nagilactones

**Figure 20:**
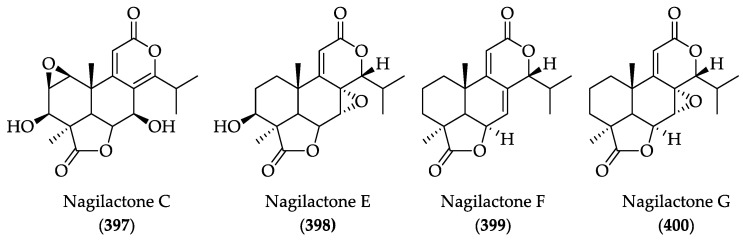
Structures of Nagilactone C, E-G (**397**–**400**).

Nagilactones belong to the group of bioactive terpenoids, which was first isolated from the evergreen tree, *Podocarpus nagi* (Tunb.) Zoll. et Moritz in the late 1960s [223]. To date, a number of nagilactones, assigned from A to L, have been isolated from various *Podocarpus species* [16]. Among those, only nagilactone C (**397**), E (**398**), F (**399**), and G (**400**) are known to exhibit anticancer activities (Figure 20).

In the study conducted by Qi et al., nagilactone C (**397**) showed potent antiproliferative activity against cancer cell lines such as MDA-MB-231, AGS (gastric cancer), and Hela cell lines, with a GI_50_ value of 2–5 mM, whereas nagilactone F (**399**) and G (**400**) displayed even more potent activity than nagilactone C (**397**) against the same cancer cell lines (IC_50_ ≈ 1 mM) [224]. In another study, nagilactones were reported to possess cytotoxic effects against P-388 leukemia cells in vitro (GI_50_ of nagilactone G (**400**) ≈ 0.25 mM and nagilactone E (**398**) = 0.25 mg/mL) [225]. Moreover, in a recent study, nagilactone E (**398**) was found to dose-dependently reduce the growth of human NSCLC cells A549 and NIC-H1975, with a GI_50_ value of 5.2 and 3.6 μM, respectively [226]. Although there is still scarce information regarding efficacy of nagilactones in vivo, Guo et al. lately demonstrated in vivo efficacy of nagilactone E (**398**) in an A549 cell lung cancer xenograft mouse model [227]. The intraperitoneal injection of 10 mg/kg/d nagilactone E (**398**) suppressed tumor growth by 62% and inhibited tumor metastasis, without obvious toxicity. Furthermore, the authors have suggested RIOK2 as a potential target of nagilactone E (**398**) after carefully reviewing their Kaplan–Meier analysis and molecular docking study results [227]. RIOK2 is an atypical serine/threonine protein kinase related to the biogenesis of ribosome. Moreover, the expression level of RIOK2 is reported to be correlated with clinical outcome in NSCLC, indicating its clinical significance [228]. However, low solubility of nagilactone E (**398**) might prevent it from further development despite the potent anticancer effects. Therefore, future works should focus on structural modification to increase drug-like properties of nagilactones and, more importantly, further validation of possible protein targets of nagilactones.

#### 2.4.4. Chemistry of Nagilactones

**Scheme 75:**
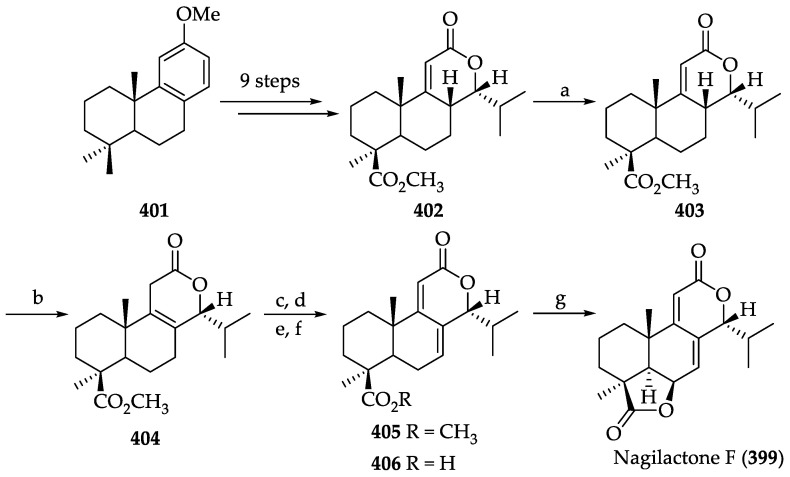
Total synthesis of nagilactone F by Hayashi et al. [229]. Reagents and conditions: (i) *t*-BuOK, DMSO; (j) hν; (k) NBS, CHCl_3_; (l) Zn, DMF; (m) H_2_SO_4_; (n) H_2_O; (O) Pb(OAc)_4_, hν.

Synthesis of nagilactone F (**399**) has been extensively studied in comparison to other nagilactones, being only one among nagilatone series with an available recent report. The first total synthesis of Nagilactone F (**399**) was reported by Hayashi et al. [229]. The synthetic approach by the group started with (+)-podocarpic acid, stereochemistry of which was already established, and structure of all intermediates formed during the course of reaction was fully characterized by the authors (Scheme 75). (+)-podocarpic acid methyl ether (**401**) was converted to the enolide (**402**) over 9 steps, which was treated with potassium *t*-butoxide in DMSO to give a diene-carboxylic acid (**403**). The authors presumed the configuration of the 8:14-double bond in **403** to be in more thermodynamically stable “*E*” form because **403** was produced under an equilibrium condition [229]. The diene-carboxylic acid (**403**) was irradiated with medium pressure mercury lamp in 95% ethanol to afford 8:9-enolide (**404**) as a single product. The enolide (**404**) underwent bromination with NBS and subsequent debromination with Zn in DMF to afford dienolide ester **405**, which was then hydrolyzed with conc. H_2_SO_4_ to furnish a dienolide acid **406**. The diene acid **406** was treated with excess Pb(OAc)_4_ in benzene to produce a *γ*-lactone in 50% yield, which was found out to be completely identical with natural nagilactone F (**399**) in IR and ^1^H-nmr comparisons [229].

**Figure 21:**
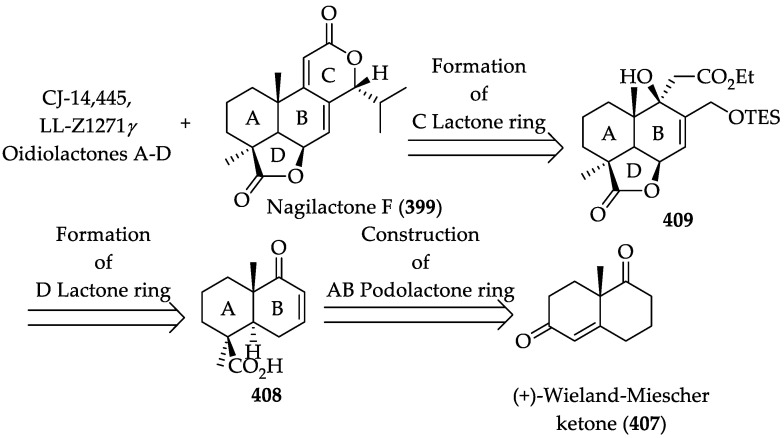
Retrosynthetic analysis of nagilactone F (**399**), CJ-14,445, LL-Z1271*γ*, Oidiolactones A-D according to Hanessian et al. [230].

Fascinatingly, Hanessian et al. reported the asymmetric total synthesis of nagilactone F from a common precursor, which also provided access to CJ-14,445, LL-Z1271*γ*, and oidilolactones A, B, C, and D (structures not shown) [230]. In this review, we will focus on the synthesis of nagilactone F from the intermediate **19**. The important synthetic strategies employed by the authors to obtain a tricyclic lactone key intermediate **19** include: (1) a Morita–Baylis–Hillman reaction, (2) a stereocontrolled bromolactonization reaction, and (3) a catalytic Reformatsky-type reaction. According to the retrosynthetic analysis provided by the group, the tricyclic lactone skeleton was sequentially prepared via construction of the AB podolactone ring, followed by the formation of D and C lactone ring (Figure 21).

**Scheme 76:**
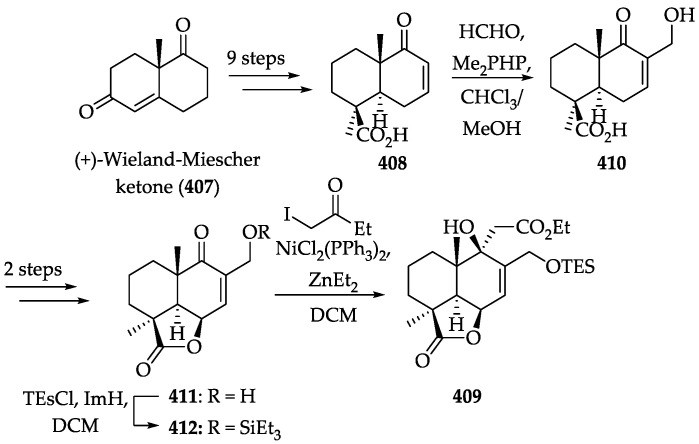
Synthesis of the common tricyclic precursor **409** [230].

The AB ring system was accomplished as a single enantiomer from the readily available Wieland-Miescher ketone (**407**) following the methods reported by Theodorakis and Danishefsky groups [231,232], which led to the fully functionalized AB ring of podolactone (**408**) over 9 steps (Scheme 76). Next, with the intermediate (**408**) in hand, construction of the D lactone ring across C-4 and C-6 was attempted. To achieve this, **408** underwent a Morita–Baylis–Hillman reaction, utilizing formaldehyde and dimethylphenylphosphine, to give **410** in excellent yield. Based on the reaction sequence developed by Welch and co-workers [233], obtained **410** was then subjected to bromolactonization, followed by elimination to give the corresponding tricyclic *γ*-lactone core **411**, which was isolated as the TES ether **412**. During the introduction of the *δ-*lactone moiety, *γ*-lactone ring opening, elimination, and poor yield occurred as major problems. This challenge was overcome by the use of intramolecular Reformatsky reaction, which had seldom been utilized in natural product synthesis [230]. The Reformatsky-type reaction between enone **412** and ethyliodoacetate in the presence of NiCl_2_(PPh_3_)_2_ and Et_3_Zn furnished the desired tertiary alcohol **409** as a single isomer.

**Scheme 77:**
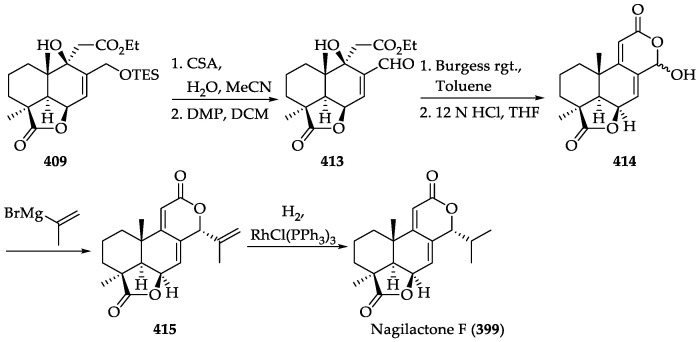
Synthesis of nagilactone F (**399**) from the intermediate **409** [230].

Toward the synthesis of nagilactone F, the common intermediate (**409**) was hydrolyzed under acidic conditions and subsequently oxidized with DMP to provide aldehyde **413**, which was further treated with the Burgess reagent followed by HCl in THF to give lactol **414** (Scheme 77). Thus, the obtained lactol **414** was treated with isopropylmagnesium bromide following the protocol reported by Barrero and co-workers [234], which, as opposed to expectations, led to low yield and moderate selectivity. Hence, the authors sought to circumvent this problem. Gratifyingly, treatment of **414** with isopropenylmagnesium bromide afforded dilactone **415** in good yield and high diasteroselectivity, which in turn underwent homogeneous hydrogenation in the presence of Wilkinsons’s catalyst to furnish nagilactone F (**399**).

Significance of this work is that it provided access to seven related norditerpenoid dilactones, including nagilactone F (**399**), from one common precursor. We envision that additional development of such convergent and divergent synthetic approaches would be necessary for the synthesis and derivatization of bioactive natural products.

## 3. Summary

The biological activities of RALs, SLs, DAGLs, and DLs, including available patent information, are summarized in Table 1. Based on the research described above, the lactones covered in this review have proved to be valuable compounds with promising bioactivities. For instance, L-783277 (**4**) exhibits a highly potent inhibitory activity against MEK (IC_50_ = 4 nM), however, it displayed low kinome-wide selectivity. This selectivity issue was overcome by rationally designed derivatives **99** and **100**. **99** is a selective ALK1 inhibitor (IC_50_ = 62 nM) and it is shown to effectively block BMP-9 induced ALK1 signaling in C2C12 cells. **100** is a dual VEGFR3 and VEGFR2 inhibitor (VEGFR3 IC_50_ = 1.15 nM, VEGFR2 IC_50_ = 3.56 nM) and its anti-lymphangiogenic and anti-angiogenic ability was demonstrated both in 3D microfluidic tumor lymphangiogenesis assay and in vivo corneal assay.

Among sesquiterpene lactones reviewed in this work, parthenolide (**183**) inhibited growth of SiHa and MCF-7 cells with an IC_50_ value of 8.42 and 9.54 μM, respectively. Furthermore, parthenolide (**183**), in combination with docetaxel, enhanced survival, and reduced metastases in a mouse xenograft model of breast cancer without any observed apparent toxicity [80]. In addition, Parthenolide (**183**), as a main component of feverfew, underwent a phase 1 clinical trial to assess its pharmacokinetics and toxicity [81,270]. Pre-existing poor solubility and bioavailability issues of parthenolide (**183**) led to development of DMAPT (**190**). Methods for preparation of parthenolide (**183**) derivatives are patented by Fusan R., et al. and Crooks, P., et al. [252,253]. DMAPT (**190**) has an increased PK profile compared to parthenolide (**183**) and significantly suppressed tumor growth in subcutaneous xenografts of A549 and UMUC-3 (transitional carcinoma cells) in athymic nude mice by 54% (100 mg/kg/day, oral) and 63% (100 mg/kg twice/day, oral), respectively. DMAPT (**190**) is reported to be in a phase 1 clinical trial in hematological malignancies in the United Kingdom [89,271,272]. DETD-35 (**232**), a derivative of deoxyelephantopin (**185**), inhibits growth of MDA-MB-231 cells with an IC_50_ value of 3.5 μM. Notably, it shows synergistic effects with vemurafenib to overcome BRAF^V600E^ mutant melanoma in a mouse xenograft model. Antrocin (**187**) inhibits growth of EGFR-harboring cell lines H441 (wt-EGFR) and H1975 (EGFR^T790M^) with an IC_50_ value of 0.75 and 0.83 μM, respectively. It suppressed tumorigenesis in lung cancer mouse xenograft in vivo. Moreover, it showed no apparent systematic in a 28-day rat study (at 37.5 mg/kg).

Among diterpene lactones, andrographolide (**373**) inhibited growth of PCa cells with an IC_50_ value of 19.95 μM and enhanced 5-FU induced apoptosis in 5-FU resistant cancer cells (HCT116/5-FUR). Use of andrographolide (**373**) derivatives in the manufacture of medicaments and structural modification of the natural product to increase biological activities are patented [205,269]. Andrographolide (**373**) is reported to be in a phase 2 clinical trial as treatment in colorectal neoplasms [273]. Nagilactone E (**398**) suppressed tumor growth by 62% in an A549 xenograft mouse model (10 mg/kg/day) and inhibited tumor metastasis without apparent toxicity.

Overall, it may be said that these natural and synthetic lactones of various classes, given the activities described in this review, possess suitable properties to initiate further preclinical and clinical studies leading to advancement into new drugs.

## 4. Conclusions and Future Prospects

The pharmacologically significant natural products have been providing inspiration and guidance to make a paradigm shift for innovative drug development strategies, which serve as a great starting point for initiation of drug discovery programs. Taking into account the researches elucidated above, the natural and synthetic lactones addressed in this review strongly suggest that they are promising therapeutic leads for oncology drug discovery program. Moreover, the synthetic studies of above-mentioned natural products and their useful analogs will open up a whole new research field, contributing to the foundation of intriguing new realm in the design and synthesis of natural products and their analogs as well.

In this review, we mainly focused on recognized natural and synthetic analogs of lactones in each classification (RALs, SLs, DAGLs, and DLs) with notable antitumor activities and we described their recent advancements made in the field of drug discovery. However, to reach a clinically viable drug from a potent natural product/-derived compound, there are still several remaining challenges to be overcome: (a) difficulties in sophisticated synthetic modification due to structural complexity, (b) relatively poor drug-like properties and target specificity, and (c) elusive exact biological targets and mechanism of action. In this light, our extensive study on structural modification of L-783277 (**4**) demonstrated that simple changes in chemical structure such as saturation or rigidification bring about significant improvements in terms of potency, target-selectivity, and pharmacokinetic property. In addition, the research mentioned above exemplified identification of possible cellular targets of DET (**185**) by using synthetic DET-related probes. We believe that ongoing studies on the synthesis and biological evaluation of such compounds result in the establishment of novel methods for innovative drug design strategies and target identification. Furthermore, we anticipate that these findings would provide biologists as well as chemists with valuable insights and resources to achieve the ultimate goal towards cancer drug discovery program.

## 5. Materials and Methods

A comprehensive search was accomplished by using the following databases to obtain the recent and relevant references in regards to natural and synthetic lactones: PubMed, Science-Direct, Springer, ACS, NIH, Google Scholar, MEDLINE, EBSCO, Web of Science, ClinicalTrials.gov, and Sci-Finder from 1946 to 2020. The keywords used include ‘natural lactones’, ‘synthetic lactones’, ‘macrocyclic lactones’, ‘resorcylic acid lactones’, ‘sesquiterpene lactones’, ‘diacylglycerol lactones’, and ‘diterpene lactones’ alone or combined with the keywords ‘derivatives’, ‘anticancer’, ‘signaling pathway’, ‘apoptosis’, ‘cell cycle’, ‘necrosis’, ‘mutation’, ‘angiogenesis’, ‘metastasis’, ‘kinase inhibition’, ‘PI3K’, ‘MAPK’, ‘ROS’, ‘target identification’, and ‘drug discovery’.

Exclusively, references in English were included in this review due to language barrier. Compound entries and references were selected according to the following criteria: availability of researches focused on (a) anticancer activities, (b) total synthesis, (c) synthetic derivatives, (d) in-vitro and in-vivo studies, and (e) clinical studies. On the other side, compound entries and references were ruled out according to the following criteria: (a) researches, which are not focused on anticancer activity, (b) researches without sufficient information on synthesis, and (c) researches without sufficient information on biological evaluation. In addition, only 4 resorcylic acid lactones have been selected as representative because biology and chemistry of resorcylic acid lactones are generally well summarized in previous reviews [12,21].

## Figures and Tables

**Table 1:** Biological activities of resorcylic acid lactones (RALs), sesquiterpene lactones (SLs), diacylglycerol lactones (DAGLs), and diterpene lactones (DLs).

Class	Compound	Summary of Biological Activities	Patents	References
RALs	Radicicol (**1**)	It selectively inhibits HSP90 function (IC_50_ = 20–23 nM). Poor in vivo activity, probably due to chemical instability in serum and its rapid conversion into inactive metabolites.	[235,236,237,238]	[24]
RALs	**32**	It selectively inhibits HSP90 function (IC_50_ = 160 nM).	[239]	[29]
RALs	KF25706 (**39**)	Significant growth-inhibitory activity against human breast carcinoma MX-1 cells transplanted into nude mice at a dose of 100 mg/kg twice daily for five consecutive iv injections.	[240]	[31]
RALs	Hypothemycin (**2**)	RAS-signaling pathway inhibitor. It also inhibits the production of several cytokines such as IL2, IL6, IFNγ, and TNFα. It inhibited the growth of HT29 and HCT116 cells in serum-free defined medium, IC_50_ = 0.078 mM and 0.90 mM, respectively.	[241,242,243]	[35]
RALs	LL-Z1640-2 (**3**)	It selectively inhibits (TGF)-β-activated kinase 1 (TAK1) with high potency TAK1, (IC_50_ = 8.1 nM). Strongly inhibition JNK/p38 pathway. It also inhibits MEK1 (IC_50_ = 411 nM) and three other MAPKKKs (IC_50_ ≥ 268 nM against MEKK1, ASK1, and MEKK4). It can be applied clinically to CNS autoimmune disorders.	[244,245,246]	[38]
RALs	**74a-d**	Its activity is comparable to **3**, but with improved solubility and pharmacokinetic properties.		[46]
RALs	ER803064 (**79**)	Increased metabolic stability and reduced potency than **3**, active in vivo, but the ED_50_ value (13.2 mg/kg, *iv*) was fairly high in regard to TNF-*α* suppression	[246,247]	[48]
RALs	**83** and **84**	A MEK1 and MEKK1 inhibitor. Similar in vitro potency to natural product **3** and improved in vivo potency by iv administration. TNF*α*-PLAP IC_50_s: 32 nM for **83**, 15 nM for **84**. ED_50_: 6.5 mg/kg for **84**.	[247,248]	[49]
RALs	**90**	Active against MNK2 kinase (IC_50_ = 7.2 μM).		[50]
RALs	L-783277 (**4**)	Highly potent inhibitory activity against MEK (IC_50_ of 4 nM). Potent inhibitory activities against several kinases including VEGFR2/3, FLT1/3/4, MEK1/2, KDR, and PDGFRα but with low kinome selectivity.	[241,243,249,250]	[47,52]
RALs	**99**	A selective and potent ALK1 inhibitor It inhibits ALK1 with an IC_50_ value of 62 nM and activates Smad4 by phosphorylating Smad1/5. It acts by selectively blocking BMP9-induced ALK1 signaling in C2C12 cells.		[56,57,58]
RALs	**100**	Potent dual VEGFR3 and VEGFR2 inhibitor (VEGFR3 IC_50_ = 1.15 nM, VEGFR2 IC_50_ = 3.56 nM). It effectively suppresses both lymphangiogenesis and angiogenesis in a 3D-microfluidic tumor lymphangiogenesis assay and in vivo corneal assay.	[251]	[54]
SLs	Parthenolide (**183**)	IC_50_ values against SiHa and MCF-7 cells (8.42 and 9.54 μM, respectively). It prevents resistance of MDA-MB-231 to doxorubicin and mitoxantrone. Cytotoxicity in a wide variety of human cancers, targeting IKK-*β*, and FAK 1 inhibition In a mouse xenograft model, it decreased tumor size in combination with docetaxel.	[252]	[80,81,82,83,253]
SLs	DMAPT (**190**)	It selectively eliminates AML stem cells. DMAPT (**190**) significantly suppressed PC-3 tumor growth until day 95 compared with control (P = 0.0007) and resulted in greater tumor control than that observed with docetaxel (P = 0.007). In A549 subcutaneous xenograft, it reduced tumor growth by 54% (100 mg/kg/day, po). In UMUC-3 (transitional carcinoma) xenograft, it suppressed tumor growth by 63% (100 mg/kg oral twice/day). It has an increased PK profile compared to **183**	[252,254]	[89,255,256]
SLs	Alantolactone (**184**)	IC_50_ values against MDA-MB 231 and HUVEC cells (40 μM and 14.2 μM, respectively).	[257]	[104]
SLs	Deoxyelephantopin (**185**)	IC_50_ values against HCT116 (colorectal), K562 (CML), KB (oral), and T47D (breast) cancer cell lines are 7.46, 4.02, 3.35, 1.86 μg/mL, respectively. IC_50_ values against PC-3, CNE, and HL-60 cells are 4.6, 2.6, and 0.9 μM, respectively. It is an inhibitor of NF-*κ*B and targets PPAR-*γ*.	[258,259]	[130,131,260,261]
SLs	DETD-35 (**232**)	IC_50_ value against MDA-MB-231 is 3.5 μM. In combination with paclitaxel, it shows synergistic effects on MDA-MB 231 cells. It also synergistic effects with vemurafenib to overcome BRAF^V600E^ mutant melanoma in a mouse model	[262]	[127,137]
SLs	Costunolide (**186**)	It shows inhibitory activities on TR-LE cells. IC_50_ value against SW-620 cells is 7.8 μM. IC_50_ value against BGC-823 cells at 24 and 48 h is 32.80 and 23.12 μM, respectively.	[263]	[162,163,264]
SLs	**264**	IC_50_ value against SW-620 cells is 3.3 μM.		[163]
SLs	Antrocin (**187**)	IC_50_ value against MDA-MB-231 cells is 0.6 μM. IC_50_ values against H441 (wt-EGFR) and H1975 (EGFR^T790M^) are 0.75 μM and 0.83 μM, respectively. It suppressed tumorigenesis in lung cancer mouse xenograft in vivo and enhanced tumor inhibitory response in treatment with JAK2 inhibitor. It showed no apparent systematic toxicity in a 28-day rat study (at 37.5 mg/kg).	[246,265,266,267]	[171,172,173]
SLs	EM23 (**188**)	IC_50_ values against Caski and SiHa cell lines are 5.8 and 6.6 μM, respectively.		[179]
SLs	Brevilin A (**189**)	It selectively inhibits growth of DU145 and MDA-MB-468. It inhibits JAK-STAT signaling pathway by attenuating JAKs activity and blocking STAT3 signaling (IC_50_ = 10.6 μM) in cancer cells.	[268]	[186]
DAGLs	AJH-836 (**315**)	It is a selective ligand for PKC*ε*. (K_i_ of PKC*α* = 46 nM, K_i_ of PKC*ε* = 1.43 nM)		[14,199]
DLs	Andrographolide (**373**)	IC_50_ value against PCa cells is 19.95 μM. IC_50_ value against A549 cells of PTX + 30 μM **373** is 0.5 nM, showing significant synergy.	[205,269]	[207,211,212,216]
DLs	19-triisopropyl andrographolide (**343**)	IC_50_ = 6.3 μM and 1.6 μM for MKN-45 and AGS cell lines, respectively.		[208]
DLs	SRJ23 (**375**)	50-fold improved IC_50_ for PCa cells (0.4 μM) than **373**.		[211]
DLs	Nagilactone C (**397**)	IC_50_ = 2–5 mM against MDA-MB-231, AGS, and Hela cell lines.		[224]
DLs	Nagilactone E (**398**)	IC_50_ = 5.2 and 3.6 μM against A549 and NIC-H1975, respectively. In an A549 xenograft mouse model (10 mg/kg/d, ip), it suppressed tumor growth by 62% and inhibited tumor metastasis without apparent toxicity		[226,227]
